# Sequencing and Comparative Genome Analysis of Two Pathogenic *Streptococcus gallolyticus* Subspecies: Genome Plasticity, Adaptation and Virulence

**DOI:** 10.1371/journal.pone.0020519

**Published:** 2011-05-25

**Authors:** I-Hsuan Lin, Tze-Tze Liu, Yu-Ting Teng, Hui-Lun Wu, Yen-Ming Liu, Keh-Ming Wu, Chuan-Hsiung Chang, Ming-Ta Hsu

**Affiliations:** 1 Institute of BioMedical Informatics, National Yang-Ming University, Taipei, Taiwan; 2 Taiwan International Graduate Program, Academia Sinica, Taipei, Taiwan; 3 VGH Yang-Ming Genome Research Center, National Yang-Ming University, Taipei, Taiwan; 4 Center for Systems and Synthetic Biology, National Yang-Ming University, Taipei, Taiwan; 5 Institute of Biochemistry and Molecular Biology, National Yang-Ming University, Taipei, Taiwan; University of Hyderabad, India

## Abstract

*Streptococcus gallolyticus* infections in humans are often associated with bacteremia, infective endocarditis and colon cancers. The disease manifestations are different depending on the subspecies of *S. gallolyticus* causing the infection. Here, we present the complete genomes of *S. gallolyticus* ATCC 43143 (biotype I) and *S.* p*asteurianus* ATCC 43144 (biotype II.2). The genomic differences between the two biotypes were characterized with comparative genomic analyses. The chromosome of ATCC 43143 and ATCC 43144 are 2,36 and 2,10 Mb in length and encode 2246 and 1869 CDS respectively. The organization and genomic contents of both genomes were most similar to the recently published *S. gallolyticus* UCN34, where 2073 (92%) and 1607 (86%) of the ATCC 43143 and ATCC 43144 CDS were conserved in UCN34 respectively. There are around 600 CDS conserved in all *Streptococcus* genomes, indicating the *Streptococcus* genus has a small core-genome (constitute around 30% of total CDS) and substantial evolutionary plasticity. We identified eight and five regions of genome plasticity in ATCC 43143 and ATCC 43144 respectively. Within these regions, several proteins were recognized to contribute to the fitness and virulence of each of the two subspecies. We have also predicted putative cell-surface associated proteins that could play a role in adherence to host tissues, leading to persistent infections causing sub-acute and chronic diseases in humans. This study showed evidence that the *S. gallolyticus* still possesses genes making it suitable in a rumen environment, whereas the ability for *S.* p*asteurianus* to live in rumen is reduced. The genome heterogeneity and genetic diversity among the two biotypes, especially membrane and lipoproteins, most likely contribute to the differences in the pathogenesis of the two *S. gallolyticus* biotypes and the type of disease an infected patient eventually develops.

## Introduction

*Streptococcus bovis*, a member of Lancefield group D streptococci, comprises a group of Gram–positive bacteria which are normal inhabitants of the gastrointestinal tract of human and animals, such as cattle, sheep, pigs, horses and dogs. In human, it is also the causative agent of bacteremia [1], [2], [3], neonatal sepsis [4], neonatal meningitis [5], adult meningitis [6] and has a well-known association with infective endocarditis (IE) [3], [7], [8], [9], colorectal carcinoma [10], [11], [12], [13], [14] and liver diseases [15], [16], [17].

In the late 1970s, the improvement in biochemical analytical methods allows the diversity among *S. bovis* strains to be recognized and this led to devising schemes to distinguish strains by biotype. Biotype I (classical *S. bovis* strains) strains can ferment mannitol and produce extracellular glucan from sucrose, whereas biotype II variants generally lack these traits. Biotype II *S. bovis* are further subdivided into biotype II.1 and biotype II.2 based on further biochemical characteristics [18], [19], [20]. In the past two decades, advancement in genotypic characterization and sequencing technology allows microbiologists to further revise the taxonomic classification of *S. bovis* [21], [22], [23], [24]. Many of the biotype I species have been reclassified as *S. gallolyticus* subsp. *gallolyticus* (here after refer to as *S. gallolyticus)*, biotype II.1 *S. bovis* as *S. infantarius* and *S. lutetiensis*, and biotype II.2 *S. bovis* as *S. gallolyticus* subsp. *pasteurianus* (here after refer to as *S. pasteurianus)*. Because of the clear association between *S. bovis* and several human diseases, it is vital to accurately distinguish these organisms and identify the differences between them in a genomic scale.

The purposes of this study were to (1) provide the first complete genomic sequence of the two subspecies *Streptococcus gallolyticus*: *S. gallolyticus* strain ATCC 43143 (biotype I) and *S. pasteurianus* strain ATCC 43144 (biotype II.2) and (2) perform comparative sequence analysis to investigate their genetic differences. Although both strains were clinical isolates originally obtained from human blood, through comparative analysis of the sequence information we found that ATCC 43143 appears to have a genome that is more adapted to ruminal environment, equipped with many enzymes for digesting plant materials. Being slightly larger in genome size than ATCC 43144, ATCC 43143 also has more genes that encode cell surface proteins and extracellular proteins that are potential virulence factors. In contrast, ATCC 43144 is more adapted to humans, losing many of the genes originally needed in the ruminal environment. Also, in some point of its existence, ATCC 43144 had accepted foreign genetic materials, specifically a 13.3-kb nisin U locus comprises 12 open reading frames, probably from the lantibiotic-producing bovine pathogen *S. uberis* [25]. These results indicate that the two closely related bacteria strains diverge in genomic structure probably through adapting to different host environment.

## Materials and Methods

### Bacterial Strains and DNA isolation

*S. gallolyticus* subsp. *gallolyticus* ATCC 43143 (F-1867, RG Knight) [26] and *S. gallolyticus* subsp. *pasteurianus* ATCC 43144 (CDC 1723-81, RG Knight) [26] were obtained from the American Type Culture Collection (ATCC). Both strains were grown in brain heart infusion broth (Becton, Dickinson and Company) at 37°C in an aerobic condition. Genomic DNAs were extracted using Wizard Genomic DNA Purification Kit (Promega) according to manufacturer's instructions.

### Genome Sequencing and Assembly

The genome of ATCC 43143 was sequenced to a 122-fold coverage using a Genome Sequencer 20 (GS 20) instruments (Roche) from one shotgun library and one paired-end library with insert size of 2- to 3-kb. The genome of ATCC 43144 was sequenced to a 34-fold coverage using GS 20 with one shotgun library. Fosmid libraries of these two strains were constructed using the CopyControl Fosmid Library Production kit (Epicentre) in the pCC1FOS vector with insert size of 30- to 40-kb. The fosmid libraries were sequenced from both ends by BigDye Terminator v3.1 chemistry and ABI 3730xl DNA analyzer (Applied Biosystems) giving around 10-fold coverage. The reads generated from the GS 20 and fosmid end sequencing were assembled by Newbler sequence assembler (version 1.1.03.24) bundled with GS 20. Gaps between the contigs were closed using fosmid end sequences as linking information and primer walking on fosmid clones and PCR from chromosomal DNA. Illumina/Solexa libraries were constructed and sequenced on a Genome Analyzer II (Illumina) with a single read module of 36 bases read-length. Low quality sequence regions of the assembled genome sequences were eliminated by aligned all Solexa reads with 73- and 190-fold of genome coverage for ATCC 43143 and ATCC43144, respectively.

### Bioinformatics Analysis

Protein coding sequences (CDS) were predicted with a combination of prokaryotic gene prediction programs, namely Glimmer v2.13 [27], Glimmer v3 [28] and GeneMarkHMM [29], with the prediction accuracy of the translation initiation site (TIS) improved by TiCo [30]. Automatic genome annotation was performed using an in-house annotation pipeline involving a collection of computational feature prediction tools. Protein function was assigned based on BLASTP similarity search against NCBI ‘nr’ (non-redundant protein) database, whereas protein similarity with KEGG protein database was used for KEGG orthology and pathway assignment [31]. Position-Specific Iterative BLAST (PSI-BLAST) search against STRING protein database [32] was used to define the clusters of orthologous group (COG) functional classification of predicted proteins. Protein domains were predicted by RPSBLAST and HMMER [33] using NCBI's Conserved Domain Database (CDD) [34] and Pfam [35] respectively. Protein subcellular localization prediction was performed by PSORTb [36]. Type I and Type II lipoprotein signal peptides were predicted using SignalP [37] and LipoP [38] respectively. Numbers of transmembrane helices in proteins were predicted using TMHMM [39]. The codon table was generated using CUSP program of EMBOSS [40], subsequently the tables was used to calculate the normalized codon adaptation index (CAI) using CAIcal [41]. Finally, CRISPRFinder was used to predict clustered regularly interspaced short palindromic repeats (CRISPRs) in the genome [42].

Transfer RNA (tRNA) and transfer-messenger RNA (tmRNA) genes were predicted using ARAGORN [43] and tRNAscan-SE [44], and RNAmmer was used to perform ribosomal RNA gene prediction [45].

### Comparative Genomic Analysis

Publically available streptococci sequences on NCBI were used for comparative analysis (http://www.ncbi.nlm.nih.gov/genomes/lproks.cgi). Artemis [46] was used for data management and DNAPlotter [47] was used for visualization of genomic features. Mauve alignment tool was used for multiple genomic sequence alignment and visualization [48]. Phylogenic analysis was performed using MEGA4 [49] with multiple sequence alignment by MAFFT [50] on streptococci 16S RNA sequences and the resulting tree visualized using Archaeopteryx (successor to ATV) [51].

### Accession Numbers

*S. gallolyticus* ATCC 43143 and *S. pasteurianus* ATCC 43144 have been deposited at **GenBank/DDBJ/EMBL** under accession numbers **AP012053** and **AP012054**, respectively.

## Results and Discussion

### Genome structure and general features of ATCC 43143 and ATCC 43144

**Figure 1:**
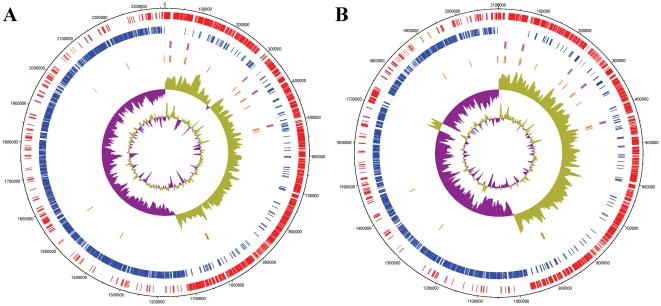
Circular representation of the *S. gallolyticus* ATCC 43143 and *S. pasteurianus* ATCC 43144 genomes. From the outside in, the outer two circles shows open reading frames oriented in the forward (red) and reverse (blue) direction, respectively. The third circle marks the rRNA gene operon (pink) and the fourth circle shows the tRNA genes (orange). The fifth circle shows GC skew, purple indicating negative values whereas olive for positive values. The inner-most circle shows the G+C% content plot.

The genome of *S. gallolyticus* ATCC 43143 and *S. pasteurianus* ATCC 43144 each comprises a single circular chromosome of 2,362,241 bp and 2,100,077 bp respectively (Figure 1). The general features are presented in Table 1. The average G+C contents of both genomes are 37% and neither contains any plasmids. There are 61 tRNA genes and five rRNA operons in each chromosomes, with most of the tRNA genes situated close to rRNA operons. A total of 2246 protein-coding genes are predicted in ATCC 43143, much similar to the recently sequenced *S. gallolyticus* UCN34 of the same biotype [52], [53], with 255 (11.4%) being either annotated as conserved hypothetical proteins or proteins with no database match. Out of the 1869 predicted CDS in the smaller ATCC 43144, 180 (9.6%) are hypothetical proteins without functional assignment.

**Table 1:** General features of the *S. gallolyticus* ATCC 43143 and *S. pasteurianus* ATCC 43144 genomes and comparison with *S. gallolyticus* UCN34.

Features	*S. gallolyticus* ATCC 43143	*S. pasteurianus* ATCC 43144	*S. gallolyticus* UCN34
**Biotype**	Type I	Type II.2	Type I
**Total Length (bp)**	2,362,241	2,100,077	2,350,911
**G+C Content (%)**	37.5%	37.4%	37.6%
**Predicted CDS**	2246	1869	2223
**Predicted Pseudogene**	49	156	37
**Coding Percentage**	87%	85%	87%
**Average Protein Length (aa)**	301	295	306
**Predicted rRNA**	15	15	18
**Predicted tRNA**	61	61	71

### Streptococci phylogeny

**Figure 2:**
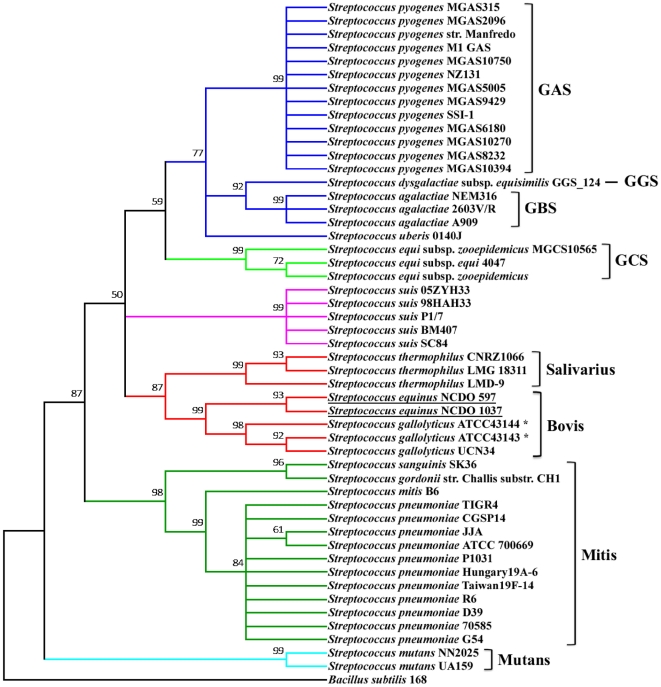
Phylogenetic relationship of *S. gallolyticus* to other sequenced streptococci. The multiple sequence alignment of 16S rRNA was constructed using MAFFT. The evolutionary history was inferred using the UPGMA method and the bootstrap consensus tree inferred from 1000 replicates. The percentage of replicate trees in which the associated taxa clustered together in the bootstrap test are shown next to the branches. The evolutionary distances were computed using the Jukes-Cantor method. All positions containing gaps and missing data were eliminated from the dataset, and a total of 1240 positions in the final dataset. *Bacillus subtilis* strain 168 was included as an outgroup. Phylogenetic analyses were conducted in MEGA4.

Streptococci phylogeny was constructed based on the 16S rRNA sequences of all sequenced bacteria of the *Streptococcus* genus. The result was shown in Figure 2. The traditional Lancefield grouping on streptococci matched the modern-day 16S rRNA analysis very well. The *S. gallolyticus* ATCC 43143, *S. gallolyticus* UCN34 and *S. pasteurianus* ATCC 43144 were of the Bovis group with ATCC 43143 phylogenetically more related to UCN34 (both biotype I) than to ATCC 43144 (biotype II.2) of the different subspecies.

### Proteomic homology analysis reveals streptococci core genome and regions of genomic plasticity

**Figure 3:**
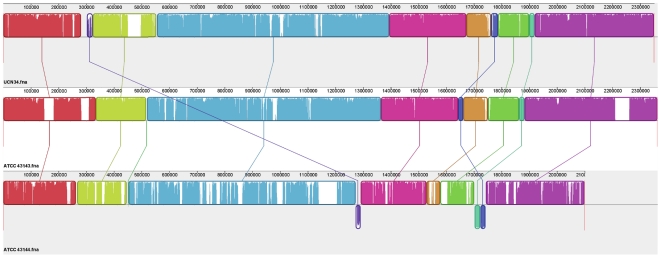
Local collinear blocks (LCBs) of the chromosomal sequences of the three strains of *S. gallolyticus*. Representation of chromosomal similarity of the three strains was generated by the Mauve alignment software. Nine local collinear blocks (LCBs) were identified with connecting lines joining the regions on the chromosomes that are homologous in the three genomes. LCBs drawn below the black horizontal line represent homology found in the reverse strand of the chromosome. Uncolored regions within the LCBs or in-between LCBs indicate the presence of strain-specific sequences.

**Figure 4:**
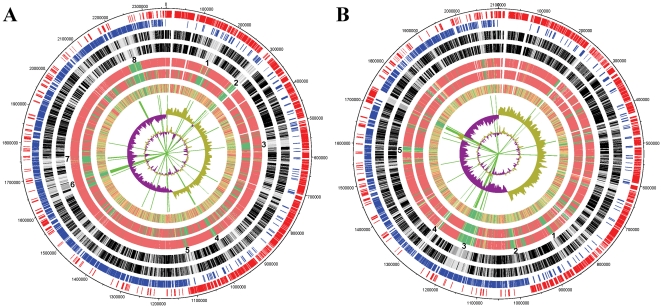
Circular representation of protein conservation of (a) *S. gallolyticus* ATCC 43143 and (b) *S. pasteurianus* ATCC 43144. From the outside in, the outer two circles showed the open reading frames (ORFs) oriented in the forward (red) and reverse (blue) directions respectively. The third and forth circles marked the homology with other sequenced bacteria and streptococci respectively, with darker the line denoting more genomes having the putative protein orthologs and whiter lines otherwise. The fifth and sixth circle shows the degree of protein sequence homology with UCN34 and the other *S. gallolyticus* strain (ATCC 43144 in figure 4a and ATCC 43143 in figure 4b) respectively, with a color-scale running from the most similar in red to least similarity in green. The seventh circle shows the normalized codon usage values of the ORFs, with a color-scale running from the higher values in red to lower values in green. The location of transposases, Tn elements and phage proteins are marked by lime colored lines. Strain-specific regions (regions of genomic plasticity) are marked by bold numbers.

Comparison in a genomic scale revealed high conservation in both the sequence and gene order of the ATCC 43143, ATCC 43144 and UCN34 genomes (Figure 3). At the same time, strain-specific regions, also known as regions of genomic plasticity (RGPs) were also identified (Figure 4). Sequence comparison against all of the other sequenced streptococcal genomes showed 91% of the CDS in ATCC 43413 and 80% in ATCC 43144 were orthologous to UCN34. Protein conservation is lower compared with other streptococci, with no more than 60% ATCC 43413 CDS and 70% ATCC 43144 CDS conserved in any single *Streptococcus* species (Table S1 and Figure S1). There are 600 ATCC 43143 CDS and 585 ATCC 43143 CDS that are conserved in all sequenced streptococci. A list of 108 conserved CDS that are completely identical in peptide sequence in ATCC 43143, ATCC 43144 and UCN34 is provided in Table S2. The average number of CDS in *Streptococcus* is roughly 2000 genes; hence the streptococci core-genome consists about 30% of the total predicted proteome. There are 99 (4%) ATCC 43143 CDS and 116 (6%) ATCC 43144 CDS not conserved in any sequenced streptococci (Table S3 and S4). The numbers rose to 410 (18%) in ATCC 43143 CDS and 217 (12%) in ATCC 43144 CDS when conservation in UCN34 was not considered, suggests the *S. gallolyticus* genomes contained more subspecies-specific genes than *S. pasteurianus*.

Eight RGPs (including 363 ORFs) and five RGPs (including 139 ORFs) were identified in ATCC 43143 and ATCC 43144 respectively (Figure 4, Table S5 and S6). These RGPs were also well-matched with regions that showed low level protein conservation compared with other streptococci. The corresponding normalized CAI (nCAI) values of these RGPs were generally lower than the rest of the genome. The CAI is a way to measure synonymous codon usage bias and expression level of a given gene [54]. Therefore high CAI value correlates with high levels of gene expression, whereas low CAI value suggests lower gene expression level and/or potential foreign origin from recent horizontal gene transfer events. Many of these regions have unusual high or low GC content as shown in Figure 4, which is an added sign of foreign origin.

In ATCC 43143, region 1, 2 and 7 consist of mainly hypothetical proteins and remnants of integrative elements. Most of the ORFs in region 1 and 7 are arranged in the directions where majority of the genes in that region resides. But, many of the ORFs in region 2 are encoded in the negative strand where genes surrounding region 2 are in positive strand. Together with low nCAI values of the genes in this region, it is a clear indication of foreign gene insertion. Region 3 includes a tryptophan operon and a WXG100 eSAT-6 secretion system that is common in Gram-positive bacteria. Most of the genes in this region were conserved between ATCC 43143 and UCN34, but absent in ATCC 43144. An ATCC 43143 strain-specific exopolysaccharide biosynthesis gene cluster was found to be located in region 4, and some of the proteins in this cluster have sequence similarity with peptides from *S. thermophilus*, *Bacteroides vulgatus* or *Clostridium botulinum*. Region 5 has several predicted transporters and enzymes of *Clostridium* and other Firmicutes origins. Also in this region, there are genes coding for the biofilm-associated proteins GtfA, GtfB and GbpC, which were also found in UCN34 but not ATCC 43144. One the other hand, the dihydroxyacetone (Dha) kinase gene cluster conserved in ATCC 43143 and ATCC 43144 in region 5 suggests they (but not UCN34) can utilize dihydroxyacetone via a PEP-dependent phosphotransferase system, hence using Dha as carbon and energy source. Or the Dha kinase would allow the utilization of glycerol for adaptation in host environment [55] or the synthesis of methylglyoxal for adaptation in certain environment [56]. A Tn916-like transposable element, composed of 16 genes with a low nCAI value, was found region 6. This region also contains one pili locus and a tannase gene that was present in UCN34 but lost in ATCC 43144. Region 8 in ATCC 43143 comprises mainly hypothetical proteins that have high sequence similarities with proteins from *S. agalactiae* that were not found in UCN34 and ATCC 43144 and the nCAI values of genes were also lower in the area.

Like region 4 in ATCC 43143, the corresponding location in ATCC 43144 (region 1) contains a strain-specific exopolysaccharide biosynthesis gene cluster with ORFs sharing similarity with proteins from *Bacillus cereus* and *Clostridium thermocellum*. A glucuronic acid utilization gene cluster was found in region 2, and a *S. uberis* nisin U-like gene locus responsible for the production and immunity of nisin-like lantibiotics was found in region 3. Both gene clusters were not found in ATCC 43143 and UCN34. Region 3 and 4 contains many ORFs coding for hypothetical proteins and Tn5252-like conjugative transposons. A large number of genes in region 3 have low nCAI value, which is a clear indication that many of the genes are of foreign origin. Region 5 contains several sugar uptake-related genes that were missing in both UCN34 and ATCC 43143, they includes endo-beta-N-acetylglucosaminidase, glucokinase, glucosidases, mannosidases and a sugar ABC transporter.

### Genome dynamics and host adaptation

Although *S. gallolyticus* ATCC 43143 and *S. pasteurianus* ATCC 43144 shares many homologous proteins, detailed comparison revealed a striking genome adaptation event occurring in the two subspecies of *S. gallolyticus*, presumably due to the different host microenvironments these two bacteria commonly resides.

It was found that ATCC 43143 retained many proteins that can transport, utilize and degrade various types of complex plant polysaccharides. The *mtlARFD* (SGGB_0982∼SGGB_0985) operon encodes the phosphoenolpyruvate (PEP)-dependent phosphotransferase system that can import and phosphorylate mannitol in the environment, where mannitol is a major photosynthetic product in plants and fungi [57], [58]. Celluloses and pectins are major carbohydrates making up the cell walls of plants, enzymes such as cellulase (encoded by SGGB_0358) and pectate lyase (SGGB_1576 and SGGB_1577) can digest these complex carbohydrates into simpler by-products. The pectinase gene in ATCC 43144 (SGPB_1461) is the truncated version of SGGB_1577. Degradation enzyme mannan endo-1,4-beta-mannosidase (encoded by SGGB_0206) in ATCC 43143 can trigger random hydrolysis of beta-1,4-mannosidic linkages in mannans, galactomannans and glucomannans, breaking up the major polymers of hemicellulose in the wall of higher plants. The extracellular fructan beta-fructosidase (also known as exo-inulinase) encoded by SGGB_0110 has a high nCAI value. The highly expressed exo-inulinase can hydrolyse fructans naturally found in many plants to take advantage of this abundant carbohydrate in rumen. Also, long-chain polysaccharides can be broken down by alpha-amylases. ATCC 43143 has four copies of the alpha-amylase genes (SGGB_0736, SGGB_0740, SGGB_1033 and SGGB_1646) whereas ATCC 43144 only has one (SGPB_0905), meaning ATCC 43143 may be more efficient in degrading complex carbohydrates. The presence of *cinA* gene (SGGB_0137) encoding the cinnamoyl ester hydrolase in ATCC 43143 that can release cinnamic acids from various plant materials (such as esterified arabinoxilan). This gene is known to be present in rumen microorganisms [59], and together with its ability to degrade a wide range of plant products, foster the hypothesis that ATCC 43143 is a rumen-adapted bacterium. Another important phenotype that differentiates the *S. gallolyticus* from *S. pasteurianus* is the ability of *S. gallolyticus* to tolerate tannic acid by producing tannase enzyme. Tannins are soluble secondary polyphenolic compounds produced by plants that pose a toxic effect to herbivores, tannin-sensitive fungi and bacteria. ATCC 43143 has two genes that encode the tannin degrading enzymes, SGGB_0917 encodes the extracellular tannase and SGGB_1624 encodes the cytoplasmic tannase, and gallic acid is produced as the major by-product. Upon oxidative breakdown, the gallic acid is converted to simple aliphatic acids and can enter citric acid cycle to be used as an alternative carbon supply [60]. Report from Noguchi et. al. showed an association between tannase-producing *Staphylococcus lugdunensis* with advanced-stage colon cancer, inspired by the association between *S. gallolyticus* and endocarditis and colon cancer [10], [14], [61], [62]. The extracellular tannase gene has an nCAI value of 1.132 (among the 10% of genes with high nCAI values) whereas the nCAI of the cytoplasmic counterpart is 1.038. The extracellular tannase of *S. gallolyticus* could be highly expressed to counteract with the high tannin rumen environment.

**Figure 5:**
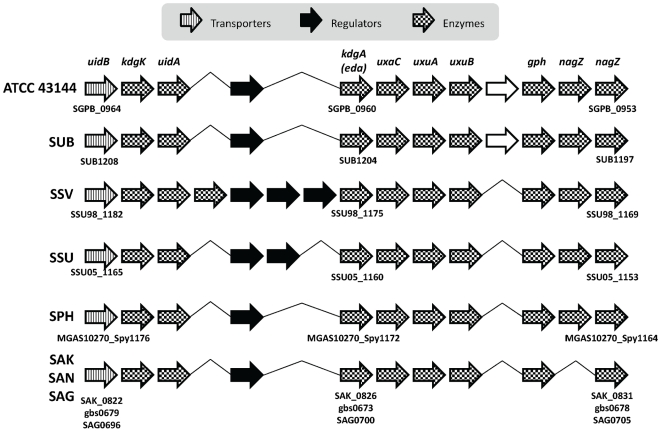
Conserved glucuronate and mannonate utilization gene cluster structure. Bacteria in the comparison includes: *S. pasteurianus* ATCC 43144, *S. uberis* 0140J (SUB), *S. suis* 98HAH33 (SSV), *S. suis* 05ZYH33 (SSU), *S. pyogenes* MGAS10270 (SPH), *S. agalactiae* A909 (SAK), *S. agalactiae* NEM316 (SAN) and *S. agalactiae* 2603 (SAG). The direction of the arrows represents the coding strand of the ORFs. The arrows were shaded to represent different functions of the ORF as shown in the legend. Hypothetical ORFs were outlined in black.

Unlike ATCC 43143, biotype II.2 ATCC 43144 has lost many of the degradation enzymes for plant materials and has sets of genes that are useful for using compounds in the nutrient-rich environment, suggesting it may have evolved to survive as human gastrointestinal bacteria. In plant and bacteria, L-rhamnose is a major constituent of the cell wall, biofilms, glycosides, and glycolipids and plays an important physiological role [63], [64], [65]. The alpha-L-rhamnosidase gene found in ATCC 43144 (SGPB_1760) allows the bacterium to utilize environmental L-rhamnose in the gut. ATCC 43144 also has enzymes that can digest short-chained and simple sugars that are abundant in the human intestine. The oligo-1,6-glucosidase encoded by *malL* (SGPB_0717) is responsible for the hydrolysis of oligosaccharide. ATCC 43144 also contains both beta-galactosidase isoenzymes: *lacZ* (SGPB_0344) and *lacG* (SGPB_0173 and SGPB_0910), which converts lactose in dairy products to galactose and glucose, whereas ATCC 43143 has the *lacG* version of the isoenzyme (SGGB_1039). Also, a conserved cluster of twelve genes (SGPB_0953∼SGPB_0964) known to be involved in beta-glucuronide and D-glucuronate degradation was identified in ATCC 43144 (Figure 5). Glucuronidation is an important detoxification pathway in vertebrates whereby glucuronic acid is linked with toxins, and proteins encoded by this gene cluster will allow the bacterium to use glucuronides as alternative carbon source. The external beta-glucuronide is exported into the cell via glucuronide transporter (UidB) and converted into D-glucuronate by beta-glucuronidase (UidA). The D-glucuronate is then converted into pyruvate and glyceraldehyde-3-phosphate by series reactions catalyzed by glucuronate isomerase (UxaC), mannonate dehydrogenase (UxuB), mannonate dehydratase (UxuA), 2-dehydro-3-deoxygluconokinase (KdgK) and keto-deoxy-phosphogluconate aldolase (KdgA) [66].

### Unique biosynthesis enzymes in ATCC 43143

Through genome comparison, several biosynthetic enzymes are found to be uniquely present in *S. gallolyticus* ATCC 43143. It was found that ATCC 43143 has the *panDCB* (SGGB_0203∼SGGB_0205) locus that is involved in pantothenate (vitamin B_5_) biosynthesis. Pantothenic acid is an essential nutrient required for the synthesis of coenzyme A and acyl carrier protein, which in turns play important roles in fatty-acid metabolism, citric acid cycle, biosynthesis of polyketides and several other reactions [67]. Without the ability to synthesize this essential compound, the survival of the bacteria could be greatly hindered. A *panC* and *panD* double-deletion *Mycobacterium tuberculosis* mutant had limited pathogenesis in mice model [67]. Like vitamin B_5_, vitamin B_6_ is an essential metabolite required as cofactor in numerous enzymatic and biochemical reactions. The deoxyxylulose 5-phosphate (DXP)-dependent biosynthesis pathway is the predominant methods where bacteria synthesize vitamin B_6_ [68], and the key enzyme in this pathway is the pyridoxal 5′-phosphate synthase consisting the synthase subunit PdxS and the glutaminase subunit PdxT [69]. In ATCC 43143, the *pdxST* gene is predicted to locate at SGGB_1182 and SGGB_1183, whereas ATCC 43144 lacks these two genes. Without the ability to catalyze the *de novo* biosynthesis of pantothenate and pyridoxal 5′-phosphate will likely confer a B_5_ and B_6_ auxotrophic phenotype in ATCC 43144.

In the genome of ATCC 43143, a region comprises the seven structural genes required for tryptophan biosynthesis was identified and it has a gene order of *trpEGDCFBA* (SGGB_0550∼SGGB_0556), a conserved organization found in many Gram-positive bacteria carrying this locus, such as *Bacillus subtilis* and other Firmicutes [70].Without the *trp* locus, ATCC 43144 will be required to uptake external tryptophan in order to have this essential amino acid for survival.

A five-gene *glg* locus (SGGB_0765∼SGGB_0770) involves in converting metabolized carbohydrates into intracellular glycogen storage polymers was found in ATCC 43143 and UCN34. The organization of the *glg* locus (*glgBCDAP*) is identical to many bacteria with the exception that the *glgD* gene in ATCC 43143 seems to have a frameshift mutation causing it to become a putative pseudogene. The ability to produce storage glycogen allows the bacteria to have a better chance of survival and prolonged the period of exposure to host tissue when sugars were depleted.

This absence of the *pan*, *dex*, *trp* and *glg* loci suggest ATCC 43144 seemed to reside in an environment generally much more nutrient-rich than ATCC 43143, and the ability of *de novo* biosynthesis of certain essential metabolites is not survival-critical and can be obtained readily from the environment, for example in human gut with nutrients from food ingestion and by-products from human gut microbiota.

### Resistance and defense mechanisms against other bacteria, bacteriophages and host's immune response

#### Antibiotic resistance

Early reports showed the *vex-vnc* locus plays a major role in autolysis and vancomycin tolerance in *S. pneumoniae* [71], [72], [73], [74]. The proposed mechanism of autolysin activation and vancomycin tolerance involves the *vex*/*pep^27^*/*vncSR* locus whereby upon binding of vancomycin onto the bacterial cell wall, it triggers the expression of the locus, the death signal peptide pep^27^ produced is transported into the extracellular space via Vex123 transporter system. The signal in turn activates VncS leading to dephosphorylation of VncR. Dephosporylated VncR causes the change in gene expression leading to the activation of major autolysin, LytA. Mutagenesis studies of this locus has shown increase tolerance to multiple antibiotics, including penicillin and vancomycin. In *S. pasteurianus* ATCC 43144, the locus encoded by SGPB_0613 to SGPB_0617 is lacking the *pep^27^* gene. The nCAI values of the genes are low and the locus is flanked by transposase and integrase, suggesting they originated possibly from *S. pneumoniae* in the past through horizontal gene transfer.

Bacteria often produce broad-spectrum antimicrobial peptides and proteins called bacteriocins to suppress surrounding bacteria to gain colonization advantageous over bacteria without immunity. In 2005, a gene locus termed *nsu*, responsible for the lantibiotic class bacteriocin nisin biosynthesis and resistance, was discover in the bovin pathogen *S. uberis* [25]. A locus similar to *nsu* was also identified in ATCC 43144 and has a low nCAI value (SGPB_1100∼SGPB_1111, Figure S2). The *nsuT* gene encodes the ABC transporter and is hypothesized to function as a lantibiotic translocator/transporter. However, in ATCC 43144, the *nsuT* gene has a point mutation. With it being a possibly pseudogene and being non-functional, it raise doubt on the ability of the bacterium to secrete nisin into the environment successfully.

**Figure 6:**
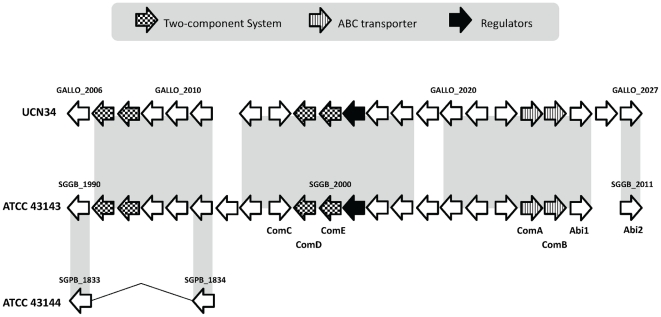
Organization of the bacteriocin *blp*-like locus in *S. gallolyticus* ATCC 43143, *S. pasteurianus* ATCC 43144 and *S. gallolyticus* UCN34. Regions with sequence similarity are shaded with light gray background. The direction of the arrows represents the coding strand of the ORFs. The arrows were shaded to represent different functions of the ORF as shown in the legend.

A bacteriocin locus containing 22 genes (SGGB_1990∼SGGB_2011) were identified in the *S. gallolyticus* ATCC 43143 genome (Figure 6), and the corresponding genomic region in ATCC 43144 is almost completely deleted. In this locus, there are five competence genes organized as two operons (*comAB* and *comCDE*), a two-component regulatory system, two bacteriocin-associated membrane-bound metalloproteases (Abi proteins, SGGB_2010∼SGGB_2011), regulatory protein BlpS, small molecular weight bacteriocins and immunity genes. Like the *pln* locus from *Lactobacillus plantarum* and *sag* locus from *S. pyogenes*, the Abi genes are located at the downstream of the bacteriocin locus in ATCC 43143, which involves in providing self-immunity against the effect of bacteriocins [75].

The coupling of competence and bacteriocin production is not an uncommon phenomenon, and even a beneficial topological arrangement. In *B. subtilis*, the *comS* gene was located within one of the operon (*srfA* operon) required for biosynthesis of a surfactin. The lysis of surrounding sensitive microorganisms makes DNA available during competence event [76], [77]. In *S. pneumoniae*, competence event is found to trigger the expression autolysins LytA and LytC [78]. In *S. mutans*, the competence-stimulating peptide (CSP) is found to induce the co-expression of genes involves in competence and bacteriocin/autolysin production, possibly an evolutionary adaption strategy, enhancing the dissemination of fitness-enhancing genes between microorganisms living in the same ecological niche. [79], [80], [81].

*beta*-Lactam antibiotics are the most widely used chemotherapeutic agents to treat bacterial infections [82]. Bacteria can gain resistance by several strategies, namely exportation of the antibiotics, decreased permeability of the bacterial outer membrane, expressing *beta*-lactamases that can hydrolyze the antibiotics, and modification of the target itself, i.e. the penicillin-binding protein (PBP). From genomic analysis, it was clear that both ATCC43143 and ATCC43144 harbor the genes for enzymatic degradation of *beta*-lactams and have a mosaic PBP gene pool.

Both *S. gallolyticus* strains possess multiple copies of beta-lactamase genes in their chromosomes, they are SGGB_0012, SGGB_0845, SGGB_1549, SGGB_1939, SGGB_1972 and SGGB_2084 in ATCC 43143, and SGPB_0014, SGPB_0724, SGPB_1447, SGPB_1788, SGPB_1815 and SGPB_1893 in ATCC 43144 respectively. Most *beta*-lactamases have a broad-spectrum profiles, together with the presence of multiple sets of *beta*-lactamases, the effectiveness of *beta*-lactam antibiotics could be greatly undermined.

Bacteria generally have three to more than eight PBPs, and their exact *in vivo* functions are mostly not known, but they are believed to function as transpeptidase, transglycosylase, and carboxypeptidase in cell wall cross-linking [83]. PBPs are divided into the high-molecular mass (HMW) and low-molecular mass (LMW) PBPs, the HMW PBPs are subdivided into class A and class B based on differences in the sequences of the N-terminal regions [83], [84]. In ATCC 43143 and ATCC 43144 six types of penicillin binding protein, including five HMW PBPs and one LMW PBP, were predicted. The penicillin-binding protein 1A (SGGB_0453 and SGPB_0380), penicillin-binding protein 1B (SGGB_0083 and SGPB_0082) and penicillin-binding protein 2A (SGGB_0128 and SGPB_0124) are considered as class A HMW PBPs, while penicillin-binding protein 2B (SGGB_0625 and SGPB_0523) and penicillin-binding protein 3 (SGGB_0442 and SGPB_0368) are class B HMW PBP. The only LMW PBP gene predicted in ATCC 43143 and ATCC 43144 is predicted to encode a D-alanyl-D-alanine carboxypeptidase (penicillin-binding protein 5/6) (SGGB_0351 and SGPB_0275).

#### CRISPR/Cas-mediated phage resistance

Clustered Regularly Interspaced Short Palindromic Repeats (CRISPR) is a common cellular defense mechanism employed by bacteria against phage infections. Based on the CRISPR classification, the CRISPR found in ATCC 43143 and ATCC 43144 belong to the “Nmeni” subtype (CRISPR/Cas Subtype Nmeni) [85]. Bacteria carrying this CRISPR subtype are vertebrate pathogens and commensals.

*S. gallolyticus* ATCC 43143 has seven CRISPR-associated genes forming two CRISPR loci, whereas *S. pasteurianus* ATCC 43144 has three CRISPR-associated genes forming a single CRISPR locus (Figure S3). The two CRISPR loci in ATCC 43143 have different repeat patterns (TGTTTTACGGTTACTTAAATCTTGAGAGTACAAAAAC and GTTTTGGAACCATTCGAAACAGCACAGCTCTAAAAC) containing 10 and 29 spacer sequences respectively. The CRISPR locus repeat pattern in ATCC 43144 is TGTTTTACGGTTACTTAAATCTTGAGAGTACAAAAAC, similar to the CRISPR1 repeat in ATCC 43143, and it contains 37 spacer sequences. Putative CRISPR leaders, defined as low-complexity and A/T-rich noncoding sequence, were found immediately upstream of the first repeat of all CRISPR loci. Sequence analysis of the leader sequences of ATCC 43143, ATCC 43144 and UCN34 revealed the leader of CRISPR1 in ATCC 43143 is identical to that in ATCC 43144, whereas the rest of the leaders share little similarity, although leader sequence conservation has previously been described [86]. The presence of TATA-like box within the leader sequence led to the speculation that leader might act as a promoter for the transcription of the CRISPR, and has been observed in archaeon *Sulfolobus acidocaldarius* [87] and *Pyrococcus furiosus* [88].

Location-wise, the two CRISPR loci in ATCC 43143 are found between 1,477,224∼1,486,444 bp and between 1,395,041∼1,397,515 bp in ATCC 43144. The genes flanking the 5′ end the CRISPR loci are found to be conserved in ATCC 43143 and ATCC 43144, whereas the genes flanking the 3′ end of the CRISPR locus in ATCC 43144 are found to be transposases, and this probably had resulted the excision of the second CRISPR locus from ATCC 43144.

Regarding spacer sequence diversity, of the total 76 spacer sequences, only three are identical in nucleotide sequence (TTGAACTCAAACAGACATTTGAAGAATGGT), and they are all located within the second CRISPR locus in ATCC 43143. There is one spacer sequence (TTAGGAGACAACGTTGTCGTTGGTGCTGGC) in ATCC 43144 was also found outside its CRISPR region. This 30-nt spacer sequence was also found in SGPB_0947 which encodes the maltose O-acetyltransferase. The first CRISPR locus in ATCC 43143 and CRISPR locus in ATCC 43144 has three CRISPR-associated proteins, Cas2, Cas1 and Csn1. The second CRISPR locus in ATCC 43143 has four CRISPR-associated proteins, Csn2, Cas2, Cas1 and Csn1. Cas1 appears to be a dsDNA endonuclease, and Cas2 may act as a sequence-specific endoribonuclease that cleaves ssRNAs. In the Nmeni subtype, the Csn1 is proposed to be a multi-domain protein, performing the functions of Cas3 and Cas4 that is missing in this subtype (i.e. helicase/exonuclease). Csn2 doesn't appear to present in all Nmeni CRISPR/cas loci and its function is unknown.

#### Polysaccharide capsule heterogeneity in ATCC 43143 and ATCC 43144

Bacterial capsule is the primary defense mechanism against host innate immune system during infection. It protects the organism from phagocytosis, and allows the bacteria to survive in the bloodstream and disseminate from the initial site of infection to other parts of the body. Therefore the capsule is often considered an important virulence factor for many human pathogens [89], [90].

**Figure 7:**
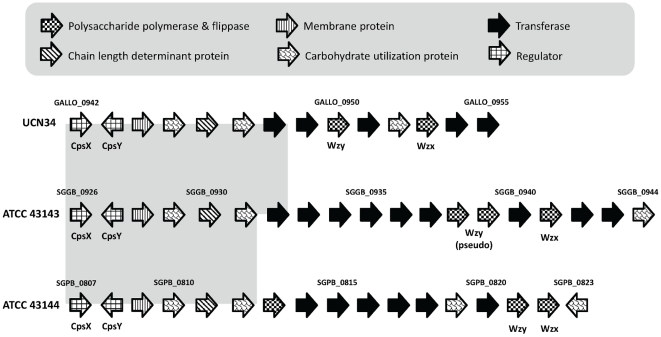
Comparison of the capsule biosynthesis loci in *S. gallolyticus* ATCC 43143, *S. pasteurianus* ATCC 43144 and *S. gallolyticus* UCN34. The 3′ regions with sequence similarity are shaded with light gray background. The direction of the arrows represents the coding strand of the ORFs. The arrows were shaded to represent different functions of the ORF as shown in the legend. Hypothetical ORFs were outlined in black.

The capsular polysaccharide (*cps*) biosynthesis locus in *S. gallolyticus* ATCC 43143 and *S. pasteurianus* ATCC 43144 is located downstream of the *deoD* gene which encodes the purine-nucleoside phosphorylase (not related to capsule biosynthesis). In ATCC 43143, the *cps* cluster consists of 19 genes (SGGB_0926 to SGGB_0944), whereas in ATCC 43144 has 17 genes (SGPB_0807 to SGPB_0823) and UCN34 has 14 genes (Figure 7). With comparative sequence analyses, it was found that the first six genes at the 5′ end of the *cps* locus were highly conserved, where the first two genes (*cpsX* and *cpsY*) encode the transcription regulators LytR and LysR respectively. The following four genes (*cpsA*, *cpsB*, *cpsC* and *cpsD*) encode the capsular biosynthesis transcriptional activator, two protein-tyrosine phosphatases and a capsular chain length determinant protein. One the other hand, the rest of the genes at the 3′ end of the *cps* locus have low nCAI values, consisting of several sugar transferases, polysaccharide polymerase and flippase, are species- or strain-specific (Table S7). In UCN 34, most of genes at the 3′ end shared high sequence and organizational similarities with *S. pneumonia*, especially *S. pneumonia* str. Him18, str. Dr. Melchior and ATCC 700669. On the other hand, the genes in ATCC 43143 and ATCC 43144 were derived from a collection of different bacteria. Considering the low sequenced similarity observed in these genes, it is possible that these capsular genes were acquired from an unknown donor bacterium that has not yet been sequenced.

The genetic organization of the *cps* locus is widely conserved in many bacteria species, the regulatory genes are often located at the 5′ end, and genes involves in sequential steps of oligosaccharide biosynthesis, modification and assembly in the 3′ end. The diversity observed in the make-up of genes in the 3′ end of the *cps* locus allows the assembly of different monosaccharids with different glycosidic linkage during capsule biosynthesis, thus introduce capsular heterogeneity and variety in antigenic properties. This phenomenon had been reported in several pneumococcal studies [91], [92], [93], [94].

### Bacterial cell-surface components and their roles in host-pathogen interactions

The interaction between bacteria and host cells involves the binding of cell surface proteins and polysaccharides to surface receptors on host tissue cells. This adherence event is the critical step in the pathogenesis of bacterial infection. *S. gallolyticus* cell surface, like many other Gram-positive bacteria, is decorated with a variety of proteins and polysaccharides that are either covalently or non-covalently bound to the bacterial cell wall. The cell-surface components of *S. gallolyticus* can be broadly divided into the following categories: (1) LPXTG-like proteins, (2) pseudopili, (3) surface lipoproteins and (4) capsule.

#### LPXTG surface proteins and characterization of multiple pilus loci

Proteins containing C-terminal cell wall sorting signal LPXTG-like motif are covalently attached to peptidoglycan by membrane-associated cysteine protease-transpeptidase sortases [95], [96], [97]. The functions of these LPXTG-containing proteins range from adhesins involving in host cell interaction and biofilm formation, antigens receptor, enzymes to virulence factors. Almost all Gram-positive bacteria have sortase-like proteins and so far, these sortases are classified into four families. In *S. gallolyticus*, two types of sortases were identified: sortase A (SGGB_0178, SGGB_1117, SGGB_1666 and SGGB_2153 in *S. gallolyticus* ATCC 43143 and SGPB_0986 in *S. pasteurianus* ATCC 43144) and family 3 sortase (SGGB_1566, SGGB_2020 and SGGB_2209 in ATCC 43143 and SGPB_1845 in ATCC 43144). In general, sortase A proteins are necessary for the anchoring of the majority of the LPXTG-containing proteins. Family 3 sortases anchor fewer proteins than class A sortases, and it recognizes a glycine residue after the LPXTG motif instead of an acidic residue.

A total of 29 LPXTG-containing proteins were identified in ATCC 43143 and 15 in ATCC 43144 (Table S8), of which 11 are conserved in both strains. Most of the shared proteins are transporter components and enzymes such as ribonucleases, lactocepin (proteinase), pullulanases and phospho-N-acetylmuramoyl-pentapeptide-transferase. In ATCC 43143, 18 LPXTG-containing proteins, including eight cell wall surface proteins and five collagen-binding proteins, are not found in ATCC 43144. The remaining five proteins are fructan beta-fructosidase (SGGB_0110), glucan-binding protein C (SGGB_1047), fimbrial subunit B protein (SGGB_1567), phosphotransferase system component protein (SGGB_1964), and bacteriocin (SGGB_2003). Four LPXTG-containing proteins uniquely found in ATCC 43144 includes a DHA2 family major facilitator superfamily (MFS) transporter (SGPB_0884), glucan-binding protein (SGPB_1131), collagen-binding Cna protein (SGPB_1661) and cell wall surface protein (SGPB_0680). Some of the LPXTG-containing proteins are highly conserved and commonly found in many other bacteria (e.g. transport system component proteins and PBP 1A), whereas some are uniquely found within some *Streptococcus* species. Two examples are SGGB_0110 and SGGB_0730. SGGB_0110 encoding the exo-inulinase is only found in *S. gallolyticus* UCN34, *S. uberis* 0140J, *S. mutans* UA159 and NN2025, *S. sanguinis* SK36 and *S. gordonii* CH1. SGGB_0730 encoding the lactocepin and it is conserved in *S. gallolyticus* UCN34, *S. mitis* B6, three stains of *S. agalactiae* (NEM316, A909 and 2603V/R), *S. thermophilus* LMD-9, *S. sanguinis* SK36, *S. gordonii* CH1 and three stains of *S. suis* (P1/7, 98HAH33 and 05ZYH33).

Bacterial pili are putative virulence factors and have been recognized as one of the mediators of initial host-pathogen interactions, by acting as an adhesin to a variety of host epithelia cells. Pili are an assembly of multimeric fibers of LPXTG-containing surface proteins.

**Figure 8:**
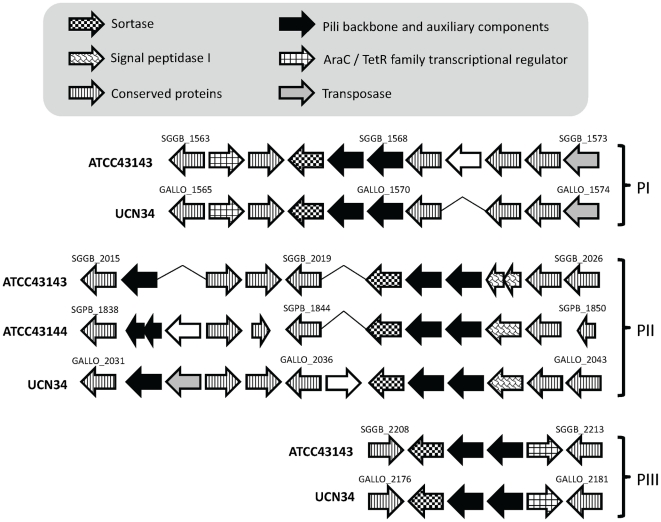
Putative pili loci in *S. gallolyticus* UCN34, *S. gallolyticus* ATCC 43143 and *S. pasteurianus* ATCC 43144. The pili loci were divided into three groups according to their genomic locations. The direction of the arrows represents the coding strand of the ORFs. The arrows were shaded to represent different functions of the ORF as shown in the legend. Hypothetical ORFs were outlined in black.

Both ATCC 43143 and UCN34 have three pili loci predicted in their genomes, whereas only one is predicted in ATCC 43144. Having multiple pili loci might mean that *S. gallolyticus* have a more complex cell surface structure than *S. pasteurianus* (Figure 8).

#### Common and unique surface lipoproteins

Lipoproteins are special class 2 signal peptide containing proteins covalently attached to membrane lipid after cleavage by signal peptidase II. Lipoproteins have been found to be involved in physiological functions such as adhesins, transporters, receptors, enzymes or virulence factors [98].

Using computational prediction methods, 52 genes in *S. gallolyticus* ATCC 43143 were predicted to encode for lipoproteins and 36 in *S. pasteurianus* ATCC 43144, and 26 are conserved in both strains (Table S9). Of the total 62 uniquely identified lipoproteins in two strains, 26 are proteins without known function and another 26 are substrate/solute-binding protein components of sugar, amino acid, iron, phosphate and other metal ion transport systems. Of the remaining 10 genes, four are conserved in both strains, they are: N-acetylmuramoyl-L-alanine amidase (SGGB_0721 and SGPB_0611), cyclophilin A (SGGB_1704 and SGPB_1517), and preprotein translocase YidC (SGGB_1801, SGGB_2060, SGPB_1631 and SGPB_1869). A L,D-transpeptidase lipoprotein (SGGB_0601) and extracellular tannase (SGGB_0917) are uniquely found in ATCC 43143, whereas carboxylesterase type B (SGPB_1074), nisin immunity protein (SGPB_1100), GNAT family acetyltransferase (SGPB_1197) and endo-beta-N-acetylglucosaminidase (SGPB_1523) appear only in ATCC 43144.

#### Adhesins associated with virulence

Based on sequence similarity comparison, several genes in *S. gallolyticus* ATCC 43143 and *S. pasteurianus* ATCC 43144 were found to be highly similar to adhesins known to be associated with virulence, endothelial cell adherence and IE in other bacteria (Table 2).

**Table 2:** List of known bacterial adhesins associated with virulence, adherence to human endothelial cells and infective endocarditis.

Genes	UCN34	ATCC 43143	ATCC 43144
*fimB*/*ssaB*/*scaA*/*psaA*/*mtsA*/*efaA*	GALLO_2047	SGGB_2030	SGPB_1854
*pilB*	GALLO_0087	SGGB_0087	SGPB_0086
*gtfbC*	GALLO_1055GALLO_1057	SGGB_1044SGGB_1046	NA
*atlA*	GALLO_1368	SGGB_1362	SGPB_1289

The substrate-binding lipoprotein MtsA is located within the *mts* operon (SGGB_2028∼SGGB_2030 and SGPB_1852∼SGPB_1854). In *S. agalactiae* and *S. pyogenes*, the *mts* operon encodes the components for the ATP-binding cassette (ABC) transport systems responsible for metal ion acquisition, such as iron, manganese, and zinc [99], [100]. Highly homologous proteins were identified in several species of streptococci and enterococci and this protein family had been designated LraI (lipoprotein receptor-associated antigen I). LraI proteins are often found to be associated with virulence. Besides MtsA, other well-studied LraI proteins include FimA from *S. parasanguis* [101], [102], FimB from *S. gallolyticus* [103], SsaB from *S. sanguis* [104], [105], ScaA from *S. gordonii* [106], PsaA of *S. pneumoniae* [107], [108], SloC from *S. mutans* [109], [110] and EfaA from *Enterococcus faecalis* [111], [112], [113].

The ATP-binding protein of the competence pseudopilus operon encoded by *comGA* (SGGB_0087 and SGPB_0086) shares significant sequence similarity with the *pilB* gene found in Group B Streptococcus (GBS). In GBS, the gene codes the major pilin subunit and has been found to facilitate the adherence and invasion of the human brain microvascular endothelial cells [114], contribute to innate immune resistance [115] and biofilm formation [116].

SGGB_1362 from ATCC 43143 and SGPB_1289 from ATCC 43144 encode the cell wall-associated autolysin and fibronectin-binding protein AtlA. The homologous protein was first identified in *S. mutans* [117], and since then this autolysin has been implicated in cellular processes such as cell separation, biofilm formation, competence and most recently a virulence factor associated with IE [118], [119].

Glucosyltransferases (GTFs) are part of the major surface protein antigens in streptococci and they are involved in cell adhesion and biofilm formation. In ATCC 43143, the proteins are encoded by the *gtfA* (SGGB_1044) and *gtfB* (SGGB_1046), whereas ATCC 43144 does not have these two genes. In *S. mutans*, GTFs are the major virulence factors in dental caries [120], [121], however when it comes to IE, the reports had been contradicting. Munro et. al. (1993) and Shun et. al. (2005) shows the Gtfs contribute to the development of infective endocarditis [122], [123], whereas Nomura at. al. (2006) and Nemoto et. al. (2008) found the *S. mutans* blood isolates were defective in Gtfs, and they had a lower susceptibility to phagocytosis and were different from the typical oral strains [124], [125].

#### Missing competence genes in ATCC 43144

In streptococci, the autoinducer peptide pheromone competence-stimulating peptide (CSP)-mediated quorum-sensing (QS) is known to involve in competence development for genetic transformation, biofilm formation, and autolysis. The gene encoding the CSP peptide is *comC*, it is often organized with *comD* and *comE* to form an operon. The peptide pheromone ComC is exported into the extracellular space by the CSP secretory apparatus ComAB [126], [127]. On the outside of the cell, the CSP signal interacts with the membrane-bound sensor kinase receptor ComD which autophosphorylates the ComE response regulator and triggers phosphorylation cascade which turns on late competence genes involved in DNA uptake, recombination and biofilm production.

In *S. gallolyticus* ATCC 43143, the competence genes *comAB* (SGGB_2008 and SGGB_2009) and *comCDE* (SGGB_1998, SGGB_1999 and SGGB_2000) are located within the bacteriocin locus. The corresponding region in *S. pasteurianus* ATCC 43144 is absent, meaning it lacks the necessary competence genes for genetic transformation through the Com system.

#### Competence pseudopilus

Beside the Com DNA binding-uptake machinery, *S. gallolyticus* ATCC 43143 and *S. pasteurianus* ATCC 43144 also have a seven-gene ComG operon (SGGB_0087∼SGGB_0093 and SGPB_0086∼SGPB_0092). The *comGA* and *comGB* are predicted to encode ABC transporter and products of *comGC*, *comGD*, *comGE*, *comGF* and *comGG* are similar to the major and minor pseudopilins. The function of the *comG* apparatus is likely to form a pilus-like structure, also called competence pseudopilus in *B. subtilis* [128], necessary to bring the exogenous DNA to a membrane DNA receptor ComEA (SGGB_0636 and SGPB_0541) during transformation. Type IV pilin and pseudopilins homologues in Gram-negative bacteria have been found to associate with virulence [129], [130], [131].

#### Lack of important biofilm formation genes in ATCC 43144

Biofilms are produced by many species of bacteria to create an extracellular matrix consisting of exopolysaccharides, proteins and DNA where mono- or multi-specific microorganisms can interact with each other and the environment [132], [133], [134]. Biofilm formation is often associated with bacterial infection and bacteria exist as biofilm are less susceptible to antimicrobial agents, inhibitors and host immune system, thereby adding to their survival and ultimately facilitate the dissemination of the pathogens to new tissues and organs [135], [136], [137].

The aggregated sticky glucan polymers are formed by the action of the GTFs that promote the cell-cell attachment and facilitate biofilm formation. As mentioned in the previous section, *S. gallolyticus* ATCC 43143 GTFs were encoded by *gtfA* (SGGB_1044) and *gtfB* (SGGB_1046), where the GtfA produces water-insoluble *alpha*-1,3- linked glucosidic polymers and GtfB makes both *alpha*-1,3- linked water-insoluble and *alpha*-1,6- linked glucosidic water-soluble polymers. In *S. pasteurianus* ATCC 43144, the corresponding region was replaced by a membrane protein and five hypothetical proteins that have no homology to any known or published protein sequences.

In close vicinity, the cell wall-anchoring glucan-binding protein C encoded by *gbpC* (SGGB_1046) is located downstream of *gtfC*. GbpC has been shown to be involved in plaque biofilm formation and infective endocarditis in several streptococci, including *S. mutans* [138].

The CovSR (also known as CsrSR) two-component system was predicted only in ATCC 43143 (SGGB_1812 and SGGB_1813) and UCN34 (GALLO_1825 and GALLO_1826) but not ATCC 43144. CovSR have been shown to regulate *gtf* genes in other *Streptococcus*. In *S. pyogenes*, Cho et. al. showed mutants lacking the *covR* gene failed to form biofilm [139]. In *S. mutans*, CovR exists as an orphan response regulator and it has been shown to be essential for biofilm development and cariogenesis [140]. With the absence of most of essential biofilm-associated genes such as *gtfA*, *gtfB*, *gbpC* and *covSR*, ATCC 43144 may not be able to form biofilm.

However, the VicRK two-component signal transduction system that are also known to regulate the transcriptional level of *gtfBC* in *S. mutans* [141] were found in both strains, encoded by SGGB_1550 and SGGB_1551 in ATCC 43143 and SGPB_1448 and SGPB_1449 in ATCC 43144. This could indicate that due to some gene deletion event in the past, the *gtf* genes previously existed in all *S. gallolyticus* species were lost in *S. pasteurianus*.

#### Identification of cell wall-anchored peptidoglycan hydrolase

Peptidoglycan hydrolases (PGHs), also referred to as autolysins, are enzymes that can cleave covalent bonds in the bacterial peptidoglycan and hence participate in daughter cell separation, peptidoglycan expansion and turnover. PGHs are exported by the dedicated system, holins, which are composed o homo-oligomeric complexes. Besides the housekeeping activities, holin-autolysin systems were also implicated in antibiotic-induced lysis [142], programmed cell death [143], biofilm formation [144] and bacterial pathogenesis by producing degraded cell wall components inflammatory components [145], [146], releasing of virulence factors and assisting bacterial adherence [147].

The Cid/Lrg operons are the well-studied holin-antiholin system encoded on the bacterial chromosome that may have a role in protein export. In streptococci, unlike *S. agalactiae* and *S. mutans* that have both *cidAB* and *lrgAB* genes, *S. gallolyticus* 43143, *S. gallolyticus* UCN34 and *S. pasteurianus* ATCC 43144 only has the *cidAB* holin homologues and the *cidA* gene in ATCC 43143 was predicted to be a pseudogene due to in-frame mutation (SGGB_0970 in ATCC 43143, SGPB_0848/SGPB_0847 in ATCC 43144 and GALLO_0983/GALLO_0982 in UCN34). Based on protein domain prediction, UCN34 has a single phage-encoded holin gene GALLO_0471 situated within a region inserted with prophage genome, whereas the corresponding chromosomal regions in ATCC 43143 and ATCC 43144 do not have prophage insertion, nor do they have any predicted phage-associated holin genes.

Table 3 showed a list of putative peptidoglycan hydrolases in ATCC 43143, ATCC 43144 and UCN34 based on computational prediction. Among the 16 autolysins, 13 proteins contain type I signal peptide residues and one has type II signal peptide residues, therefore they are exported via the general secretory (Sec) pathway. Of the reminding two autolysins, one is a phage-associated cell wall hydrolase encoded by GALLO_0472. Together with the holin gene GALLO_0471, they formed a holin-lysin lysis cassette (*lytPR*) found in bacteriophages. The other autolysin is a lysozyme (1,4-beta-N-acetylmuramidase) that was conserved in all three *S. gallolyticus* strains, and is likely to be dependent on holins for export. Based on nCAI calculation, the extracellular peptidoglycan hydrolases encoded by SGGB_0018 in ATCC 43143 and SGPB_0021 in ATCC 43144 both have high nCAI values of 1.197 and 1.181 and ranked 25^th^ and 30^th^ among all the genes respectively. This enzyme is likely the major peptidoglycan hydrolase produced in these bacteria.

**Table 3:** Predicted peptidoglycan hydrolases in *S. gallolyticus* ATCC 43143, *S. gallolyticus* UCN34 and *S. pasteurianus* ATCC 43144.

ATCC 43143	ATCC 43144	UCN34	Descriptions	Signal Peptide Prediction	PSORTb Prediction
SGGB_0018	SGPB_0021	GALLO_0019	extracellular peptidoglycan hydrolase	SpI	Extracellular
SGGB_0176	-	-	signal peptide containing protein	SpI	Unknown
SGGB_0281	-	-	CHAP domain containing protein	SpI	Unknown
SGGB_0313	SGPB_0234	GALLO_0240	N-acetylmuramoyl-L-alanine amidase	SpI	Extracellular
-	-	GALLO_0472	phage-associated cell wall hydrolase	CYT	Unknown
SGGB_0721	SGPB_0611	GALLO_0740	N-acetylmuramoyl-L-alanine amidase	SpII	Extracellular
SGGB_1191	-	GALLO_1197	signal peptide containing protein	SpI	Cellwall
SGGB_1301	SGPB_1211	GALLO_1307	extracellular mannosyl-glycoprotein endo-beta-N-acetylglucosaminidase	SpI	Extracellular
SGGB_1362	SGPB_1290	GALLO_1368	serotype determinant, cell wall hydrolase/autolysin	SpI	Unknown
SGGB_1513	SGPB_1412	GALLO_1518	lysozyme (1,4-beta-N-acetylmuramidase)	CYT	Unknown
SGGB_1667	-	GALLO_1652	signal peptide containing protein	SpI	Cytoplasmic Membrane
SGGB_1973	-	GALLO_1989	signal peptide containing protein	SpI	Unknown
SGGB_2159	-	-	putative glucosaminidase	SpI	Unknown
SGGB_2169	-	-	cell surface-associated protein autolysin AtlA	SpI	Unknown
SGGB_2276	SGPB_2002	GALLO_2243	transglycosylase-like extracellular protein	SpI	Extracellular
SGGB_2277	SGPB_2003	GALLO_2244	LysM domain containing extracellular protein	SpI	Extracellular

#### ESAT-6 secretion pathway in *S. gallolyticus*

The ESAT-6 secretion system (Ess) pathway was first identified in *Mycobacterium tuberculosis* where important etiological agents of human tuberculosis (TB), ESAT-6 and CFP-10, are secreted via this pathway [148]. The ESAT-6 homologues have been identified in various Gram-positive bacteria, including *Staphylococcus aureus*, *Bacillus subtilis*, *Bacillus anthracis*, *Listeria innocua*, *Listeria monocytogenes*, *Clostridium acetobutylicum*, *Corynebacterium diphtheriae* and *Streptomyces coelicolor*. Due to the presence of a central WXG motif in this family of 100-residue proteins, the pathway is also referred to as WXG100 secretion system (Wss) and most recently as the Type VII secretion system [149], [150]. The presence of streptococcal ESAT-6 homologue was first reported in *S. gordonii* (Challis) [151] and the crystal structure of EsxA from *S. agalactiae* was determined recently (PDB ID: 3O9O and 3GWK) [152]. In this study, a putative Ess gene cluster containing a 97-residue ESAT-6 homologue (*esxA*) and six other genes (*essA*, *esaB*, *essB*, *essC*, *esaA* and *esaC*) implicated in the translocation of EsxA were identified in ATCC 43143 (RGP 3) and UCN34. Unlike some Gram-positive bacteria that possess two WXG100 proteins, EsxA and EsxB, which can form heterodimers, *S. gallolyticus* only has one WXG100 protein. The *esxA* gene of ATCC 43143 has an unusual high nCAI value (1.193) and was ranked the 29^th^ among all the genes in the genome. This suggests that it can be highly expressed upon activation and could be an important factor contributing to *S. gallolyticus* pathogenesis. The *S. gallolyticus* EsxA proteins (SGGB_0519 in ATCC 43143 and GALLO_0553 in UCN34) share 35% amino acid sequence identity with other predicted streptococcal EsxA homologues, namely *S. sanguinis*, *S. agalactiae*, *S. gordonii* and *S. equi* subsp. *zooepidemicus*. With the discovery of increasing number of bacteria possessing the components for the Ess pathway, it is plausible to consider the WXG proteins might not be the only target of this secretion system and other yet-to-be-identified effector proteins might locate outside the Ess gene locus.

### Concluding remarks

In summary, we report the first complete genome of *S. pasteurianus* (reference strain ATCC 43144), the genomic sequence of *S. gallolyticus* reference strain ATCC 43144 and their in silico analyses. The *S. gallolyticus* (formerly known as *S. bovis* biotype I) is a known human pathogen which has been shown to be associated with serious illnesses such as IE and colorectal cancer, whereas *S. pasteurianus* (biotype II.2) causes neonatal sepsis and meningitis in infants and adults.

Through analyzing the RGPs, we provided evidence of an association between genome plasticity and genome adaptive evolution. Although both ATCC strains were isolated in human blood, the gene contents of the ATCC 43143 RGPs suggest it is still largely a ruminal strain, whereas ATCC 43144 has a streamlined genome, possibly evolved to adapt to a non-rumen environment. The additional biosynthesis gene clusters found in each strain could relate to their fitness under specific conditions. Considering both *S. gallolyticus* subspecies were highly similar in their genomic contents, the processes of genome reduction/expansion were a much recent event due to environmental and host adaptation, moving from a herbivore to man. With the completion of *S. gallolyticus* TX20005 (also known as biotype I strain 2703) genome sequencing around the corner [153], [154], we hope with this work, microbiologists and clinician scientists can gain further understanding of the *S. gallolyticus* core genome and the effect of genomic differences on their virulence and pathogenesis.

This study contributes to our understanding of the pathogenesis of this species by delineating not only the known, but novel putative virulence factors, and also genes and processes that would aid the bacteria to colonize, flourish and cause disease. Future studies will focus on elucidating the precise roles of the novel lipoproteins predicted in this work and gain insights into the *S. gallolyticus* pathogenesis.

## Supporting Information

Figure S1 **Radar plot showing protein conservation between 49 streptococci with** ***S. gallolyticus*** **ATCC 43143 and** ***S. pasteurianus*** **ATCC 43144.**
(TIF)

Figure S2 **Comparison of the nisin locus in** ***Lactococcus lactis*** **subsp.** ***lactis*** **,** ***S. uberis*** **strain 42 and** ***S. pasteurianus*** **ATCC 43144.** (a) The Nis/Nus locus gene order and (b) multiple sequence alignment of NisA/NusA peptide.
(TIF)

Figure S3 **Organization of CRISPR/** ***cas*** **systems present in (a)** ***S. gallolyticus*** **ATCC 43143 and (b)** ***S. pasteurianus*** **ATCC 43144.** For each organism, the gene organization is showed on the top, with CRISPR-associated genes in black and the repeat-spacer array in blue. Below, the CRISPR repeats are indicated by black boxes, spacers are indicated by white diamonds and leader in red. Bottom shows the consensus repeat sequence.
(TIF)

Table S1 **Protein conservation between 49 sequenced Streptococcal genomes with the** ***S. gallolyticus*** **ATCC 43143 and** ***S. pasteurianus*** **ATCC 43144.** A summary table showing the percentage of protein conserved in streptococci.
(DOC)

Table S2 **List of identical CDS in ATCC 43143, ATCC 43144 and UCN34.** A table listing the 108 proteins conserved in all three *S. gallolyticus* strains.
(DOC)

Table S3 **List of ATCC 43143 unique CDS not found in other sequenced Streptococci.** A table listing the 99 *S. gallolyticus* ATCC 43143-specific proteins.
(DOC)

Table S4 **List of ATCC 43144 unique CDS not found in other sequenced Streptococci.** A table listing the 116 *S. pasteurianus* ATCC 43144-specific proteins.
(DOC)

Table S5 **List of regions of genomic plasticity (RGPs) in the ATCC 43143 genome.** A table listing the identified RGPs in *S. gallolyticus* ATCC 43143.
(DOC)

Table S6 **List of regions of genomic plasticity (RGPs) in the ATCC 43144 genome.** A table listing the identified RGPs in *S. pasteurianus* ATCC 43144.
(DOC)

Table S7 **Protein conservation of the** ***cps*** **loci of** ***S. gallolyticus*** **ATCC 43143,** ***S. pasteurianus*** **ATCC 43144 and** ***S. gallolyticus*** **UCN34.** A summary table showing the BLASTP results of the genes in the *cps* loci.
(DOC)

Table S8 **List of proteins contain the LPXTG anchoring motifs in** ***S. gallolyticus*** **ATCC 43143 and** ***S. pasteurianus*** **ATCC 43144.**
(DOC)

Table S9 **List of predicted lipoproteins in** ***S. gallolyticus*** **ATCC 43143 and** ***S. pasteurianus*** **ATCC 43144.**
(DOC)

## References

[1] Genta PR, Carneiro L, Genta EN (1998). Streptococcus bovis bacteremia: unusual complications.. South Med J.

[2] Jean SS, Teng LJ, Hsueh PR, Ho SW, Luh KT (2004). Bacteremic Streptococcus bovis infections at a university hospital, 1992-2001.. J Formos Med Assoc.

[3] Tripodi MF, Fortunato R, Utili R, Triassi M, Zarrilli R (2005). Molecular epidemiology of Streptococcus bovis causing endocarditis and bacteraemia in Italian patients.. Clin Microbiol Infect.

[4] Gavin PJ, Thomson RB, Horng SJ, Yogev R (2003). Neonatal sepsis caused by Streptococcus bovis variant (biotype II/2): report of a case and review.. J Clin Microbiol.

[5] Onoyama S, Ogata R, Wada A, Saito M, Okada K (2009). Neonatal bacterial meningitis caused by Streptococcus gallolyticus subsp. pasteurianus.. J Med Microbiol.

[6] Sturt AS, Yang L, Sandhu K, Pei Z, Cassai N (2010). Streptococcus gallolyticus subspecies pasteurianus (biotype II/2), a newly reported cause of adult meningitis.. J Clin Microbiol.

[7] Duval X, Papastamopoulos V, Longuet P, Benoit C, Perronne C (2001). Definite streptococcus bovis endocarditis: characteristics in 20 patients.. Clin Microbiol Infect.

[8] Herrero IA, Rouse MS, Piper KE, Alyaseen SA, Steckelberg JM (2002). Reevaluation of Streptococcus bovis endocarditis cases from 1975 to 1985 by 16S ribosomal DNA sequence analysis.. J Clin Microbiol.

[9] Kupferwasser I, Darius H, Muller AM, Mohr-Kahaly S, Westermeier T (1998). Clinical and morphological characteristics in Streptococcus bovis endocarditis: a comparison with other causative microorganisms in 177 cases.. Heart.

[10] Klein RS, Recco RA, Catalano MT, Edberg SC, Casey JI (1977). Association of Streptococcus bovis with carcinoma of the colon.. N Engl J Med.

[11] Klein RS, Catalano MT, Edberg SC, Casey JI, Steigbigel NH (1979). Streptococcus bovis septicemia and carcinoma of the colon.. Ann Intern Med.

[12] Darjee R, Gibb AP (1993). Serological investigation into the association between Streptococcus bovis and colonic cancer.. J Clin Pathol.

[13] Gupta A, Madani R, Mukhtar H (2010). Streptococcus bovis endocarditis, a silent sign for colonic tumour.. Colorectal Dis.

[14] Waisberg J, Matheus Cde O, Pimenta J (2002). Infectious endocarditis from Streptococcus bovis associated with colonic carcinoma: case report and literature review.. Arq Gastroenterol.

[15] Gonzlez-Quintela A, Martinez-Rey C, Castroagudin JF, Rajo-Iglesias MC, Dominguez-Santalla MJ (2001). Prevalence of liver disease in patients with Streptococcus bovis bacteraemia.. J Infect.

[16] Zarkin BA, Lillemoe KD, Cameron JL, Effron PN, Magnuson TH (1990). The triad of Streptococcus bovis bacteremia, colonic pathology, and liver disease.. Ann Surg.

[17] Tripodi MF, Adinolfi LE, Ragone E, Durante Mangoni E, Fortunato R (2004). Streptococcus bovis endocarditis and its association with chronic liver disease: an underestimated risk factor.. Clin Infect Dis.

[18] Facklam RR (1972). Recognition of group D streptococcal species of human origin by biochemical and physiological tests.. Appl Microbiol.

[19] Farrow JAE, Kruse J, Phillips BA, Bramley AJ, Collins MD (1984). Taxonomic studies on Streptococcus bovis and Streptococcus equinus: Description of Streptococcus alactolyticus sp. nov. and Streptococcus saccharolyticus sp. nov.. Syst Appl Microbiol.

[20] Parker MT, Ball LC (1976). Streptococci and aerococci associated with systemic infection in man.. J Med Microbiol.

[21] Beck M, Frodl R, Funke G (2008). Comprehensive study of strains previously designated Streptococcus bovis consecutively isolated from human blood cultures and emended description of Streptococcus gallolyticus and Streptococcus infantarius subsp. coli.. J Clin Microbiol.

[22] Facklam R (2002). What happened to the streptococci: overview of taxonomic and nomenclature changes.. Clin Microbiol Rev.

[23] Schlegel L, Grimont F, Ageron E, Grimont PA, Bouvet A (2003). Reappraisal of the taxonomy of the Streptococcus bovis/Streptococcus equinus complex and related species: description of Streptococcus gallolyticus subsp. gallolyticus subsp. nov., S. gallolyticus subsp. macedonicus subsp. nov. and S. gallolyticus subsp. pasteurianus subsp. nov.. Int J Syst Evol Microbiol.

[24] Poyart C, Quesne G, Trieu-Cuot P (2002). Taxonomic dissection of the Streptococcus bovis group by analysis of manganese-dependent superoxide dismutase gene (sodA) sequences: reclassification of ‘Streptococcus infantarius subsp. coli’ as Streptococcus lutetiensis sp. nov. and of Streptococcus bovis biotype 11.2 as Streptococcus pasteurianus sp. nov.. Int J Syst Evol Microbiol.

[25] Wirawan RE, Klesse NA, Jack RW, Tagg JR (2006). Molecular and genetic characterization of a novel nisin variant produced by Streptococcus uberis.. Appl Environ Microbiol.

[26] Knight RG, Shlaes DM (1985). Physiological Characteristics and Deoxyribonucleic Acid Relatedness of Human Isolates of Streptococcus bovis and Streptococcus bovis (var.).. Int J Syst Bacteriol.

[27] Delcher AL, Harmon D, Kasif S, White O, Salzberg SL (1999). Improved microbial gene identification with GLIMMER.. Nucleic Acids Res.

[28] Delcher AL, Bratke KA, Powers EC, Salzberg SL (2007). Identifying bacterial genes and endosymbiont DNA with Glimmer.. Bioinformatics.

[29] Lukashin AV, Borodovsky M (1998). GeneMark.hmm: new solutions for gene finding.. Nucleic Acids Res.

[30] Tech M, Pfeifer N, Morgenstern B, Meinicke P (2005). TICO: a tool for improving predictions of prokaryotic translation initiation sites.. Bioinformatics.

[31] Aoki-Kinoshita KF, Kanehisa M (2007). Gene annotation and pathway mapping in KEGG.. Methods Mol Biol.

[32] Jensen LJ, Kuhn M, Stark M, Chaffron S, Creevey C (2009). STRING 8–a global view on proteins and their functional interactions in 630 organisms.. Nucleic Acids Res.

[33] Eddy SR (2008). A probabilistic model of local sequence alignment that simplifies statistical significance estimation.. PLoS Comput Biol.

[34] Marchler-Bauer A, Anderson JB, Chitsaz F, Derbyshire MK, DeWeese-Scott C (2009). CDD: specific functional annotation with the Conserved Domain Database.. Nucleic Acids Res.

[35] Finn RD, Mistry J, Tate J, Coggill P, Heger A (2010). The Pfam protein families database.. Nucleic Acids Res.

[36] Gardy JL, Laird MR, Chen F, Rey S, Walsh CJ (2005). PSORTb v.2.0: expanded prediction of bacterial protein subcellular localization and insights gained from comparative proteome analysis.. Bioinformatics.

[37] Bendtsen JD, Nielsen H, von Heijne G, Brunak S (2004). Improved prediction of signal peptides: SignalP 3.0.. J Mol Biol.

[38] Juncker AS, Willenbrock H, Von Heijne G, Brunak S, Nielsen H (2003). Prediction of lipoprotein signal peptides in Gram-negative bacteria.. Protein Sci.

[39] Krogh A, Larsson B, von Heijne G, Sonnhammer EL (2001). Predicting transmembrane protein topology with a hidden Markov model: application to complete genomes.. J Mol Biol.

[40] Rice P, Longden I, Bleasby A (2000). EMBOSS: the European Molecular Biology Open Software Suite.. Trends Genet.

[41] Puigbo P, Bravo IG, Garcia-Vallve S (2008). E-CAI: a novel server to estimate an expected value of Codon Adaptation Index (eCAI).. BMC Bioinformatics.

[42] Grissa I, Vergnaud G, Pourcel C (2007). CRISPRFinder: a web tool to identify clustered regularly interspaced short palindromic repeats.. Nucleic Acids Res.

[43] Laslett D, Canback B (2004). ARAGORN, a program to detect tRNA genes and tmRNA genes in nucleotide sequences.. Nucleic Acids Res.

[44] Lowe TM, Eddy SR (1997). tRNAscan-SE: a program for improved detection of transfer RNA genes in genomic sequence.. Nucleic Acids Res.

[45] Lagesen K, Hallin P, Rodland EA, Staerfeldt HH, Rognes T (2007). RNAmmer: consistent and rapid annotation of ribosomal RNA genes.. Nucleic Acids Res.

[46] Rutherford K, Parkhill J, Crook J, Horsnell T, Rice P (2000). Artemis: sequence visualization and annotation.. Bioinformatics.

[47] Carver T, Thomson N, Bleasby A, Berriman M, Parkhill J (2009). DNAPlotter: circular and linear interactive genome visualization.. Bioinformatics.

[48] Darling AC, Mau B, Blattner FR, Perna NT (2004). Mauve: multiple alignment of conserved genomic sequence with rearrangements.. Genome Res.

[49] Tamura K, Dudley J, Nei M, Kumar S (2007). MEGA4: Molecular Evolutionary Genetics Analysis (MEGA) software version 4.0.. Mol Biol Evol.

[50] Katoh K, Kuma K, Toh H, Miyata T (2005). MAFFT version 5: improvement in accuracy of multiple sequence alignment.. Nucleic Acids Res.

[51] Zmasek CM, Eddy SR (2001). ATV: display and manipulation of annotated phylogenetic trees.. Bioinformatics.

[52] Unite de Biologie des Bacteries Intracellulaires IP, France (2010). Streptococcus gallolyticus subsp. gallolyticus UCN34 Genome Project.

[53] Rusniok C, Couve E, Da Cunha V, El Gana R, Zidane N (2010). Genome sequence of Streptococcus gallolyticus: insights into its adaptation to the bovine rumen and its ability to cause endocarditis.. J Bacteriol.

[54] Sharp PM, Li WH (1987). The codon Adaptation Index–a measure of directional synonymous codon usage bias, and its potential applications.. Nucleic Acids Res.

[55] Bizzini A, Zhao C, Budin-Verneuil A, Sauvageot N, Giard JC (2010). Glycerol is metabolized in a complex and strain-dependent manner in Enterococcus faecalis.. J Bacteriol.

[56] Ferguson GP, Totemeyer S, MacLean MJ, Booth IR (1998). Methylglyoxal production in bacteria: suicide or survival?. Arch Microbiol.

[57] Henstra SA, Tolner B, ten Hoeve Duurkens RH, Konings WN, Robillard GT (1996). Cloning, expression, and isolation of the mannitol transport protein from the thermophilic bacterium Bacillus stearothermophilus.. J Bacteriol.

[58] Behrens S, Mitchell W, Bahl H (2001). Molecular analysis of the mannitol operon of Clostridium acetobutylicum encoding a phosphotransferase system and a putative PTS-modulated regulator.. Microbiology.

[59] Dalrymple BP, Swadling Y, Cybinski DH, Xue GP (1996). Cloning of a gene encoding cinnamoyl ester hydrolase from the ruminal bacterium Butyrivibrio fibrisolvens E14 by a novel method.. FEMS Microbiol Lett.

[60] Bhat TK, Singh B, Sharma OP (1998). Microbial degradation of tannins–a current perspective.. Biodegradation.

[61] Noguchi N, Ohashi T, Shiratori T, Narui K, Hagiwara T (2007). Association of tannase-producing Staphylococcus lugdunensis with colon cancer and characterization of a novel tannase gene.. J Gastroenterol.

[62] Ellmerich S, Scholler M, Duranton B, Gosse F, Galluser M (2000). Promotion of intestinal carcinogenesis by Streptococcus bovis.. Carcinogenesis.

[63] Avila M, Jaquet M, Moine D, Requena T, Pelaez C (2009). Physiological and biochemical characterization of the two alpha-L-rhamnosidases of Lactobacillus plantarum NCC245.. Microbiology.

[64] Abdel-Mawgoud AM, Lepine F, Deziel E (2010). Rhamnolipids: diversity of structures, microbial origins and roles.. Appl Microbiol Biotechnol.

[65] Cui Z, Maruyama Y, Mikami B, Hashimoto W, Murata K (2006). Crystallization and preliminary crystallographic analysis of the family GH78 alpha-L-rhamnosidase RhaB from Bacillus sp. GL1.. Acta Crystallogr Sect F Struct Biol Cryst Commun.

[66] Rivolta C, Soldo B, Lazarevic V, Joris B, Mauel C (1998). A 35.7 kb DNA fragment from the Bacillus subtilis chromosome containing a putative 12.3 kb operon involved in hexuronate catabolism and a perfectly symmetrical hypothetical catabolite-responsive element.. Microbiology.

[67] Sambandamurthy VK, Wang X, Chen B, Russell RG, Derrick S (2002). A pantothenate auxotroph of Mycobacterium tuberculosis is highly attenuated and protects mice against tuberculosis.. Nat Med.

[68] Tanaka T, Tateno Y, Gojobori T (2005). Evolution of vitamin B6 (pyridoxine) metabolism by gain and loss of genes.. Mol Biol Evol.

[69] Strohmeier M, Raschle T, Mazurkiewicz J, Rippe K, Sinning I (2006). Structure of a bacterial pyridoxal 5′-phosphate synthase complex.. Proc Natl Acad Sci U S A.

[70] Gutierrez-Preciado A, Yanofsky C, Merino E (2007). Comparison of tryptophan biosynthetic operon regulation in different Gram-positive bacterial species.. Trends Genet.

[71] Novak R, Charpentier E, Braun JS, Tuomanen E (2000). Signal transduction by a death signal peptide: uncovering the mechanism of bacterial killing by penicillin.. Mol Cell.

[72] Novak R, Henriques B, Charpentier E, Normark S, Tuomanen E (1999). Emergence of vancomycin tolerance in Streptococcus pneumoniae.. Nature.

[73] Mitchell LS, Tuomanen EI (2002). Molecular analysis of antibiotic tolerance in pneumococci.. Int J Med Microbiol.

[74] Haas W, Sublett J, Kaushal D, Tuomanen EI (2004). Revising the role of the pneumococcal vex-vncRS locus in vancomycin tolerance.. J Bacteriol.

[75] Kjos M, Snipen L, Salehian Z, Nes IF, Diep DB (2010). The abi proteins and their involvement in bacteriocin self-immunity.. J Bacteriol.

[76] D'Souza C, Nakano MM, Zuber P (1994). Identification of comS, a gene of the srfA operon that regulates the establishment of genetic competence in Bacillus subtilis.. Proc Natl Acad Sci U S A.

[77] Hamoen LW, Eshuis H, Jongbloed J, Venema G, van Sinderen D (1995). A small gene, designated comS, located within the coding region of the fourth amino acid-activation domain of srfA, is required for competence development in Bacillus subtilis.. Mol Microbiol.

[78] Moscoso M, Claverys JP (2004). Release of DNA into the medium by competent Streptococcus pneumoniae: kinetics, mechanism and stability of the liberated DNA.. Mol Microbiol.

[79] Kreth J, Merritt J, Shi W, Qi F (2005). Co-ordinated bacteriocin production and competence development: a possible mechanism for taking up DNA from neighbouring species.. Mol Microbiol.

[80] van der Ploeg JR (2005). Regulation of bacteriocin production in Streptococcus mutans by the quorum-sensing system required for development of genetic competence.. J Bacteriol.

[81] Perry JA, Jones MB, Peterson SN, Cvitkovitch DG, Levesque CM (2009). Peptide alarmone signalling triggers an auto-active bacteriocin necessary for genetic competence.. Mol Microbiol.

[82] Fisher JF, Meroueh SO, Mobashery S (2005). Bacterial resistance to beta-lactam antibiotics: compelling opportunism, compelling opportunity.. Chem Rev.

[83] Massova I, Mobashery S (1998). Kinship and diversification of bacterial penicillin-binding proteins and beta-lactamases.. Antimicrob Agents Chemother.

[84] Ghuysen JM (1994). Molecular structures of penicillin-binding proteins and beta-lactamases.. Trends Microbiol.

[85] Haft DH, Selengut J, Mongodin EF, Nelson KE (2005). A guild of 45 CRISPR-associated (Cas) protein families and multiple CRISPR/Cas subtypes exist in prokaryotic genomes.. PLoS Comput Biol.

[86] Lillestol RK, Redder P, Garrett RA, Brugger K (2006). A putative viral defence mechanism in archaeal cells.. Archaea.

[87] Lillestol RK, Shah SA, Brugger K, Redder P, Phan H (2009). CRISPR families of the crenarchaeal genus Sulfolobus: bidirectional transcription and dynamic properties.. Mol Microbiol.

[88] Hale C, Kleppe K, Terns RM, Terns MP (2008). Prokaryotic silencing (psi)RNAs in Pyrococcus furiosus.. RNA.

[89] Llull D, Lopez R, Garcia E (2001). Genetic bases and medical relevance of capsular polysaccharide biosynthesis in pathogenic streptococci.. Curr Mol Med.

[90] Lowe BA, Miller JD, Neely MN (2007). Analysis of the polysaccharide capsule of the systemic pathogen Streptococcus iniae and its implications in virulence.. Infect Immun.

[91] Aanensen DM, Mavroidi A, Bentley SD, Reeves PR, Spratt BG (2007). Predicted functions and linkage specificities of the products of the Streptococcus pneumoniae capsular biosynthetic loci.. J Bacteriol.

[92] Bentley SD, Aanensen DM, Mavroidi A, Saunders D, Rabbinowitsch E (2006). Genetic analysis of the capsular biosynthetic locus from all 90 pneumococcal serotypes.. PLoS Genet.

[93] Mavroidi A, Aanensen DM, Godoy D, Skovsted IC, Kaltoft MS (2007). Genetic relatedness of the Streptococcus pneumoniae capsular biosynthetic loci.. J Bacteriol.

[94] Varvio SL, Auranen K, Arjas E, Makela PH (2009). Evolution of the capsular regulatory genes in Streptococcus pneumoniae.. J Infect Dis.

[95] Navarre WW, Schneewind O (1999). Surface proteins of gram-positive bacteria and mechanisms of their targeting to the cell wall envelope.. Microbiol Mol Biol Rev.

[96] Mazmanian SK, Ton-That H, Schneewind O (2001). Sortase-catalysed anchoring of surface proteins to the cell wall of Staphylococcus aureus.. Mol Microbiol.

[97] Ton-That H, Marraffini LA, Schneewind O (2004). Protein sorting to the cell wall envelope of Gram-positive bacteria.. Biochim Biophys Acta.

[98] Sutcliffe IC, Russell RR (1995). Lipoproteins of gram-positive bacteria.. J Bacteriol.

[99] Sun X, Ge R, Chiu JF, Sun H, He QY (2008). Lipoprotein MtsA of MtsABC in Streptococcus pyogenes primarily binds ferrous ion with bicarbonate as a synergistic anion.. FEBS Lett.

[100] Bray BA, Sutcliffe IC, Harrington DJ (2009). Expression of the MtsA lipoprotein of Streptococcus agalactiae A909 is regulated by manganese and iron.. Antonie Van Leeuwenhoek.

[101] Burnette-Curley D, Wells V, Viscount H, Munro CL, Fenno JC (1995). FimA, a major virulence factor associated with Streptococcus parasanguis endocarditis.. Infect Immun.

[102] Viscount HB, Munro CL, Burnette-Curley D, Peterson DL, Macrina FL (1997). Immunization with FimA protects against Streptococcus parasanguis endocarditis in rats.. Infect Immun.

[103] Vollmer T, Hinse D, Kleesiek K, Dreier J (2010). Interactions between endocarditis-derived Streptococcus gallolyticus subsp. gallolyticus isolates and human endothelial cells.. BMC Microbiol.

[104] Ganeshkumar N, Hannam PM, Kolenbrander PE, McBride BC (1991). Nucleotide sequence of a gene coding for a saliva-binding protein (SsaB) from Streptococcus sanguis 12 and possible role of the protein in coaggregation with actinomyces.. Infect Immun.

[105] Das S, Kanamoto T, Ge X, Xu P, Unoki T (2009). Contribution of lipoproteins and lipoprotein processing to endocarditis virulence in Streptococcus sanguinis.. J Bacteriol.

[106] Kolenbrander PE, Andersen RN, Ganeshkumar N (1994). Nucleotide sequence of the Streptococcus gordonii PK488 coaggregation adhesin gene, scaA, and ATP-binding cassette.. Infect Immun.

[107] Berry AM, Paton JC (1996). Sequence heterogeneity of PsaA, a 37-kilodalton putative adhesin essential for virulence of Streptococcus pneumoniae.. Infect Immun.

[108] Rajam G, Anderton JM, Carlone GM, Sampson JS, Ades EW (2008). Pneumococcal surface adhesin A (PsaA): a review.. Crit Rev Microbiol.

[109] Kitten T, Munro CL, Michalek SM, Macrina FL (2000). Genetic characterization of a Streptococcus mutans LraI family operon and role in virulence.. Infect Immun.

[110] Paik S, Brown A, Munro CL, Cornelissen CN, Kitten T (2003). The sloABCR operon of Streptococcus mutans encodes an Mn and Fe transport system required for endocarditis virulence and its Mn-dependent repressor.. J Bacteriol.

[111] Lowe AM, Lambert PA, Smith AW (1995). Cloning of an Enterococcus faecalis endocarditis antigen: homology with adhesins from some oral streptococci.. Infect Immun.

[112] Singh KV, Coque TM, Weinstock GM, Murray BE (1998). In vivo testing of an Enterococcus faecalis efaA mutant and use of efaA homologs for species identification.. FEMS Immunol Med Microbiol.

[113] Semedo T, Santos MA, Lopes MF, Figueiredo Marques JJ, Barreto Crespo MT (2003). Virulence factors in food, clinical and reference Enterococci: A common trait in the genus?. Syst Appl Microbiol.

[114] Maisey HC, Hensler M, Nizet V, Doran KS (2007). Group B streptococcal pilus proteins contribute to adherence to and invasion of brain microvascular endothelial cells.. J Bacteriol.

[115] Maisey HC, Quach D, Hensler ME, Liu GY, Gallo RL (2008). A group B streptococcal pilus protein promotes phagocyte resistance and systemic virulence.. FASEB J.

[116] Konto-Ghiorghi Y, Mairey E, Mallet A, Dumenil G, Caliot E (2009). Dual role for pilus in adherence to epithelial cells and biofilm formation in Streptococcus agalactiae.. PLoS Pathog.

[117] Shibata Y, Kawada M, Nakano Y, Toyoshima K, Yamashita Y (2005). Identification and characterization of an autolysin-encoding gene of Streptococcus mutans.. Infect Immun.

[118] Ahn SJ, Burne RA (2006). The atlA operon of Streptococcus mutans: role in autolysin maturation and cell surface biogenesis.. J Bacteriol.

[119] Jung CJ, Zheng QH, Shieh YH, Lin CS, Chia JS (2009). Streptococcus mutans autolysin AtlA is a fibronectin-binding protein and contributes to bacterial survival in the bloodstream and virulence for infective endocarditis.. Mol Microbiol.

[120] Kuramitsu HK (1993). Virulence factors of mutans streptococci: role of molecular genetics.. Crit Rev Oral Biol Med.

[121] Yamashita Y, Bowen WH, Burne RA, Kuramitsu HK (1993). Role of the Streptococcus mutans gtf genes in caries induction in the specific-pathogen-free rat model.. Infect Immun.

[122] Shun CT, Lu SY, Yeh CY, Chiang CP, Chia JS (2005). Glucosyltransferases of viridans streptococci are modulins of interleukin-6 induction in infective endocarditis.. Infect Immun.

[123] Munro CL, Macrina FL (1993). Sucrose-derived exopolysaccharides of Streptococcus mutans V403 contribute to infectivity in endocarditis.. Mol Microbiol.

[124] Nomura R, Nakano K, Nemoto H, Fujita K, Inagaki S (2006). Isolation and characterization of Streptococcus mutans in heart valve and dental plaque specimens from a patient with infective endocarditis.. J Med Microbiol.

[125] Nemoto H, Nakano K, Nomura R, Ooshima T (2008). Molecular characterization of Streptococcus mutans strains isolated from the heart valve of an infective endocarditis patient.. J Med Microbiol.

[126] Havarstein LS, Hakenbeck R, Gaustad P (1997). Natural competence in the genus Streptococcus: evidence that streptococci can change pherotype by interspecies recombinational exchanges.. J Bacteriol.

[127] Kleerebezem M, Quadri LE, Kuipers OP, de Vos WM (1997). Quorum sensing by peptide pheromones and two-component signal-transduction systems in Gram-positive bacteria.. Mol Microbiol.

[128] Chen I, Provvedi R, Dubnau D (2006). A macromolecular complex formed by a pilin-like protein in competent Bacillus subtilis.. J Biol Chem.

[129] Lory S (1998). Secretion of proteins and assembly of bacterial surface organelles: shared pathways of extracellular protein targeting.. Curr Opin Microbiol.

[130] Collyn F, Lety MA, Nair S, Escuyer V, Ben Younes A (2002). Yersinia pseudotuberculosis harbors a type IV pilus gene cluster that contributes to pathogenicity.. Infect Immun.

[131] Kulkarni R, Dhakal BK, Slechta ES, Kurtz Z, Mulvey MA (2009). Roles of putative type II secretion and type IV pilus systems in the virulence of uropathogenic Escherichia coli.. PLoS One.

[132] Whitchurch CB, Tolker-Nielsen T, Ragas PC, Mattick JS (2002). Extracellular DNA required for bacterial biofilm formation.. Science.

[133] Hall-Stoodley L, Stoodley P (2009). Evolving concepts in biofilm infections.. Cell Microbiol.

[134] Moscoso M, Garcia E, Lopez R (2009). Pneumococcal biofilms.. Int Microbiol.

[135] Costerton JW, Stewart PS, Greenberg EP (1999). Bacterial biofilms: a common cause of persistent infections.. Science.

[136] Bogaert D, De Groot R, Hermans PW (2004). Streptococcus pneumoniae colonisation: the key to pneumococcal disease.. Lancet Infect Dis.

[137] Mah TF, O'Toole GA (2001). Mechanisms of biofilm resistance to antimicrobial agents.. Trends Microbiol.

[138] Nomura R, Nakano K, Ooshima T (2004). Contribution of glucan-binding protein C of Streptococcus mutans to bacteremia occurrence.. Arch Oral Biol.

[139] Cho KH, Caparon MG (2005). Patterns of virulence gene expression differ between biofilm and tissue communities of Streptococcus pyogenes.. Mol Microbiol.

[140] Idone V, Brendtro S, Gillespie R, Kocaj S, Peterson E (2003). Effect of an orphan response regulator on Streptococcus mutans sucrose-dependent adherence and cariogenesis.. Infect Immun.

[141] Senadheera MD, Guggenheim B, Spatafora GA, Huang YC, Choi J (2005). A VicRK signal transduction system in Streptococcus mutans affects gtfBCD, gbpB, and ftf expression, biofilm formation, and genetic competence development.. J Bacteriol.

[142] Kohanski MA, Dwyer DJ, Collins JJ (2010). How antibiotics kill bacteria: from targets to networks.. Nat Rev Microbiol.

[143] Lewis K (2000). Programmed death in bacteria.. Microbiol Mol Biol Rev.

[144] Bayles KW (2007). The biological role of death and lysis in biofilm development.. Nat Rev Microbiol.

[145] Majcherczyk PA, Langen H, Heumann D, Fountoulakis M, Glauser MP (1999). Digestion of Streptococcus pneumoniae cell walls with its major peptidoglycan hydrolase releases branched stem peptides carrying proinflammatory activity.. J Biol Chem.

[146] Ginsburg I (2002). The role of bacteriolysis in the pathophysiology of inflammation, infection and post-infectious sequelae.. APMIS.

[147] Ahn SJ, Rice KC, Oleas J, Bayles KW, Burne RA (2010). The Streptococcus mutans Cid and Lrg systems modulate virulence traits in response to multiple environmental signals.. Microbiology.

[148] Simeone R, Bottai D, Brosch R (2009). ESX/type VII secretion systems and their role in host-pathogen interaction.. Curr Opin Microbiol.

[149] Abdallah AM, Gey van Pittius NC, Champion PA, Cox J, Luirink J (2007). Type VII secretion–mycobacteria show the way.. Nat Rev Microbiol.

[150] Bitter W, Houben EN, Bottai D, Brodin P, Brown EJ (2009). Systematic genetic nomenclature for type VII secretion systems.. PLoS Pathog.

[151] Pallen MJ (2002). The ESAT-6/WXG100 superfamily – and a new Gram-positive secretion system?. Trends Microbiol.

[152] Shukla A, Pallen M, Anthony M, White SA (2010). The homodimeric GBS1074 from Streptococcus agalactiae.. Acta Crystallogr Sect F Struct Biol Cryst Commun.

[153] Baylor College of Medicine HGSC, USA (2010). Streptococcus gallolyticus subsp. gallolyticus TX20005 Genome Project..

[154] Sillanpaa J, Nallapareddy SR, Qin X, Singh KV, Muzny DM (2009). A collagen-binding adhesin, Acb, and ten other putative MSCRAMM and pilus family proteins of Streptococcus gallolyticus subsp. gallolyticus (Streptococcus bovis Group, biotype I).. J Bacteriol.

