# Supplementary figures and images for: Sequencing and Comparative Genome Analysis of Two Pathogenic Streptococcus gallolyticus Subspecies: Genome Plasticity, Adaptation and Virulence
Source: PLoS One. 2011 May 25;6(5):e20519. doi: 10.1371/journal.pone.0020519 (PMC3102119; doi:10.1371/journal.pone.0020519)

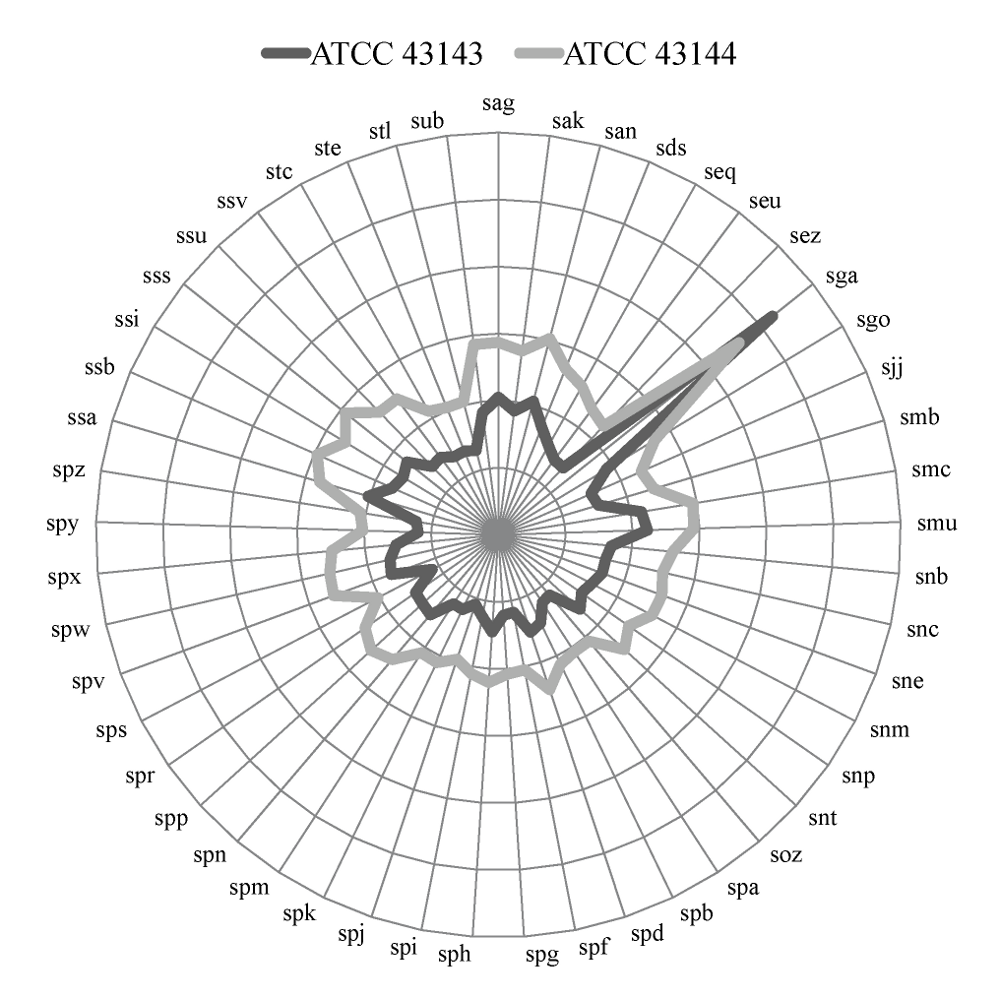

Supplement: Figure S1 — Radar plot showing protein conservation between 49 streptococci with S. gallolyticus ATCC 43143 and S. pasteurianus ATCC 43144. (TIF) [file pone.0020519.s001.tif]

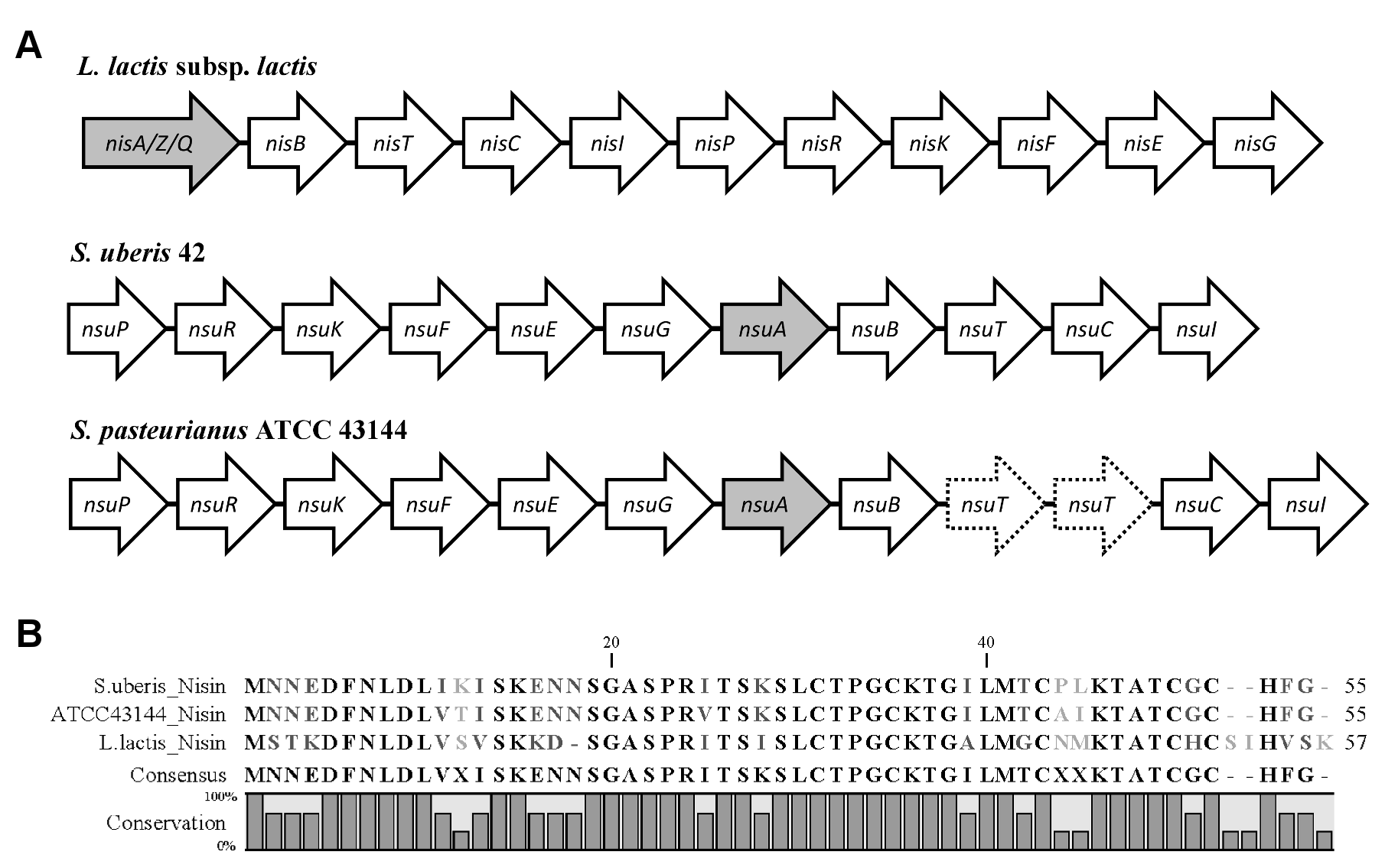

Supplement: Figure S2 — Comparison of the nisin locus in Lactococcus lactis subsp. lactis , S. uberis strain 42 and S. pasteurianus ATCC 43144. (a) The Nis/Nus locus gene order and (b) multiple sequence alignment of NisA/NusA peptide. (TIF) [file pone.0020519.s002.tif]

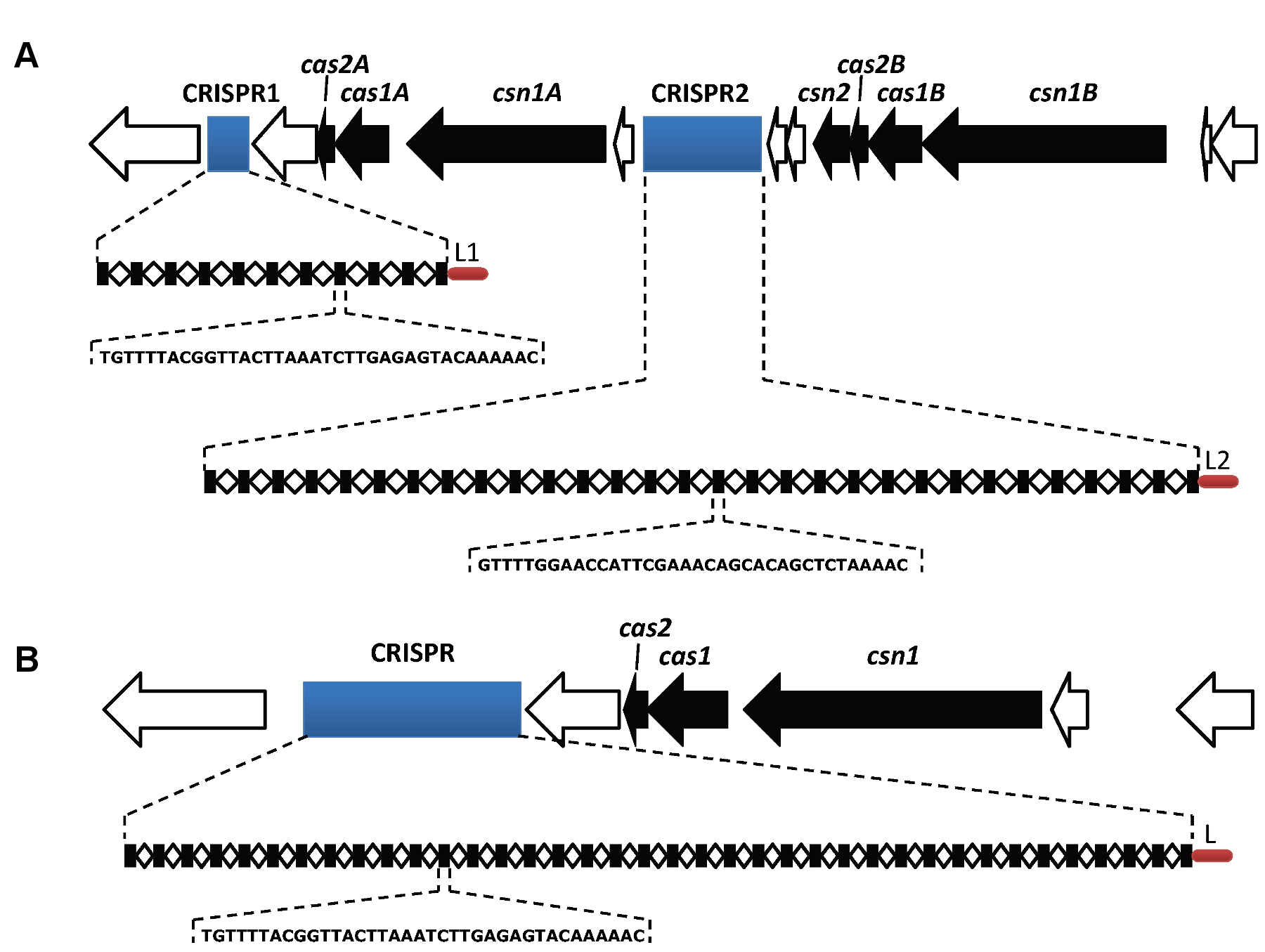

Supplement: Figure S3 — Organization of CRISPR/ cas systems present in (a) S. gallolyticus ATCC 43143 and (b) S. pasteurianus ATCC 43144. For each organism, the gene organization is showed on the top, with CRISPR-associated genes in black and the repeat-spacer array in blue. Below, the CRISPR repeats are indicated by black boxes, spacers are indicated by white diamonds and leader in red. Bottom shows the consensus repeat sequence. (TIF) [file pone.0020519.s003.tif]
