# Supplementary material for: Sequencing and Comparative Genome Analysis of Two Pathogenic Streptococcus gallolyticus Subspecies: Genome Plasticity, Adaptation and Virulence
Source: PLoS One. 2011 May 25;6(5):e20519. doi: 10.1371/journal.pone.0020519 (PMC3102119; doi:10.1371/journal.pone.0020519)
Supplement: Table S1 — Protein conservation between 49 sequenced Streptococcal genomes with the S. gallolyticus ATCC 43143 and S. pasteurianus ATCC 43144. A summary table showing the percentage of protein conserved in streptococci. (DOC) [file pone.0020519.s004.doc]

**Additional File 1. Protein conservation between 49 sequenced Streptococcal genomes with the *S. gallolyticus* ATCC 43143 and *S. pasteurianus* ATCC 43144.** A summary table showing the percentage of protein conserved in streptococci.

| **Names** | **Code** | **RefSeq** | **Released** | **ATCC 43143 ^a)^** | **ATCC 43144 ^b)^** | **ATCC 43143 (%) ^c)^** | **ATCC 43144 (%) ^d)^** |
| --- | --- | --- | --- | --- | --- | --- | --- |
| *Streptococcus agalactiae* 2603V/R | sag | NC_004116 | 2002 | 1359 | 1285 | 61% | 69% |
| *Streptococcus agalactiae* A909 | sak | NC_007432 | 2005 | 1317 | 1265 | 59% | 68% |
| *Streptococcus agalactiae* NEM316 | san | NC_004368 | 2002 | 1361 | 1314 | 61% | 70% |
| *Streptococcus dysgalactiae* subsp. *equisimilis* GGS_124 | sds | NC_012891 | 2009 | 1287 | 1248 | 57% | 67% |
| *Streptococcus equi* subsp. *equi* 4047 | seu | NC_012471 | 2009 | 1212 | 1185 | 54% | 63% |
| *Streptococcus equi* subsp. *zooepidemicus* | seq | NC_012470 | 2009 | 1241 | 1223 | 55% | 65% |
| *Streptococcus equi* subsp. *zooepidemicus* MGCS10565 | sez | NC_011134 | 2008 | 1211 | 1170 | 54% | 63% |
| *Streptococcus gallolyticus* UCN34 | sga | NC_013798 | 2010 | 2073 | 1607 | 92% | 86% |
| *Streptococcus gordonii* str. Challis substr. CH1 | sgo | NC_009785 | 2007 | 1321 | 1258 | 59% | 67% |
| *Streptococcus mitis* B6 | smb | NC_013853 | 2010 | 1248 | 1200 | 56% | 64% |
| *Streptococcus mutans* NN2025 | smc | NC_013928 | 2010 | 1383 | 1298 | 62% | 69% |
| *Streptococcus mutans* UA159 | smu | NC_004350 | 2002 | 1398 | 1293 | 62% | 69% |
| *Streptococcus pneumoniae* 670-6B | snb | NC_014498 | 2010 | 1279 | 1237 | 57% | 66% |
| *Streptococcus pneumoniae* 70585 | snm | NC_012468 | 2009 | 1251 | 1232 | 56% | 66% |
| *Streptococcus pneumoniae* AP200 | snp | NC_014494 | 2010 | 1238 | 1191 | 55% | 64% |
| *Streptococcus pneumoniae* ATCC 700669 | sne | NC_011900 | 2009 | 1269 | 1234 | 57% | 66% |
| *Streptococcus pneumoniae* CGSP14 | spw | NC_010582 | 2008 | 1270 | 1230 | 57% | 66% |
| *Streptococcus pneumoniae* D39 | spd | NC_008533 | 2006 | 1241 | 1202 | 55% | 64% |
| *Streptococcus pneumoniae* G54 | spx | NC_011072 | 2008 | 1242 | 1214 | 55% | 65% |
| *Streptococcus pneumoniae* Hungary19A-6 | spv | NC_010380 | 2008 | 1280 | 1237 | 57% | 66% |
| *Streptococcus pneumoniae* JJA | sjj | NC_012466 | 2009 | 1238 | 1184 | 55% | 63% |
| *Streptococcus pneumoniae* P1031 | spp | NC_012467 | 2009 | 1241 | 1227 | 55% | 66% |
| *Streptococcus pneumoniae* R6 | spr | NC_003098 | 2001 | 1236 | 1201 | 55% | 64% |
| *Streptococcus pneumoniae* Taiwan19F-14 | snt | NC_012469 | 2009 | 1268 | 1223 | 56% | 65% |
| *Streptococcus pneumoniae* TCH8431/19A | snc | NC_014251 | 2010 | 1267 | 1218 | 56% | 65% |
| *Streptococcus pneumoniae* TIGR4 | spn | NC_003028 | 2001 | 1249 | 1204 | 56% | 64% |
| *Streptococcus pyogenes* M1 GAS | spy | NC_002737 | 2001 | 1167 | 1126 | 52% | 60% |
| *Streptococcus pyogenes* MGAS10270 | sph | NC_008022 | 2006 | 1224 | 1160 | 54% | 62% |
| *Streptococcus pyogenes* MGAS10394 | spa | NC_006086 | 2004 | 1169 | 1136 | 52% | 61% |
| *Streptococcus pyogenes* MGAS10750 | spi | NC_008024 | 2006 | 1177 | 1142 | 52% | 61% |
| *Streptococcus pyogenes* MGAS2096 | spj | NC_008023 | 2006 | 1143 | 1113 | 51% | 60% |
| *Streptococcus pyogenes* MGAS315 | spg | NC_004070 | 2002 | 1168 | 1137 | 52% | 61% |
| *Streptococcus pyogenes* MGAS5005 | spz | NC_007297 | 2005 | 1176 | 1138 | 52% | 61% |
| *Streptococcus pyogenes* MGAS6180 | spb | NC_007296 | 2005 | 1225 | 1148 | 55% | 61% |
| *Streptococcus pyogenes* MGAS8232 | spm | NC_003485 | 2002 | 1174 | 1140 | 52% | 61% |
| *Streptococcus pyogenes* MGAS9429 | spk | NC_008021 | 2006 | 1175 | 1143 | 52% | 61% |
| *Streptococcus pyogenes* NZ131 | soz | NC_011375 | 2008 | 1163 | 1137 | 52% | 61% |
| *Streptococcus pyogenes* SSI-1 | sps | NC_004606 | 2003 | 1148 | 1127 | 51% | 60% |
| *Streptococcus pyogenes* str. Manfredo | spf | NC_009332 | 2007 | 1162 | 1131 | 52% | 60% |
| *Streptococcus sanguinis* SK36 | ssa | NC_009009 | 2007 | 1353 | 1267 | 60% | 68% |
| *Streptococcus suis* 05ZYH33 | ssu | NC_009442 | 2007 | 1217 | 1224 | 54% | 65% |
| *Streptococcus suis* 98HAH33 | ssv | NC_009443 | 2007 | 1222 | 1221 | 54% | 65% |
| *Streptococcus suis* BM407 | ssb | NC_012926 | 2009 | 1286 | 1300 | 57% | 70% |
| *Streptococcus suis* P1/7 | ssi | NC_012925 | 2009 | 1271 | 1246 | 57% | 67% |
| *Streptococcus suis* SC84 | sss | NC_012924 | 2009 | 1290 | 1293 | 57% | 69% |
| *Streptococcus thermophilus* CNRZ1066 | stc | NC_006449 | 2004 | 1199 | 1144 | 53% | 61% |
| *Streptococcus thermophilus* LMD-9 | ste | NC_008532 | 2006 | 1201 | 1131 | 53% | 60% |
| *Streptococcus thermophilus* LMG 18311 | stl | NC_006448 | 2004 | 1191 | 1132 | 53% | 61% |
| *Streptococcus uberis* 0140J | sub | NC_012004 | 2009 | 1311 | 1283 | 58% | 69% |

1. Number of ATCC 43143 ORFs that were homologous with the compared *Streptococcus* bacterium.
2. Number of ATCC 43144 ORFs that were homologous with the compared *Streptococcus* bacterium.
3. Percentage of ATCC 43143 ORFs that were homologous with the compared *Streptococcus* bacterium.
4. Percentage of ATCC 43144 ORFs that were homologous with the compared *Streptococcus* bacterium.
