# Supplementary material for: Sequencing and Comparative Genome Analysis of Two Pathogenic Streptococcus gallolyticus Subspecies: Genome Plasticity, Adaptation and Virulence
Source: PLoS One. 2011 May 25;6(5):e20519. doi: 10.1371/journal.pone.0020519 (PMC3102119; doi:10.1371/journal.pone.0020519)
Supplement: Table S2 — List of identical CDS in ATCC 43143, ATCC 43144 and UCN34. A table listing the 108 proteins conserved in all three S. gallolyticus strains. (DOC) [file pone.0020519.s005.doc]

**Table S2. List of identical CDS in ATCC 43143, ATCC 43144 and UCN34.** A table listing the 108 proteins conserved in all three S. gallolyticus strains.

| **ATCC 43143 ORF ID** | **ATCC 43144 ORF ID** | **UCN34 ORF ID** | **Descriptions** |
| --- | --- | --- | --- |
| SGGB_0019 | SGPB_0022 | sga:GALLO_0020 | prsA.1 ribose-phosphate pyrophosphokinase |
| SGGB_0052 | SGPB_0050 | sga:GALLO_0052 | rplW 50S ribosomal protein L23 |
| SGGB_0054 | SGPB_0052 | sga:GALLO_0054 | rpsS 30S ribosomal protein S19 |
| SGGB_0055 | SGPB_0053 | sga:GALLO_0055 | rplV 50S ribosomal protein L22 |
| SGGB_0057 | SGPB_0055 | sga:GALLO_0057 | rplP 50S ribosomal protein L16 |
| SGGB_0058 | SGPB_0056 | sga:GALLO_0058 | rpmC 50S ribosomal protein L29 |
| SGGB_0059 | SGPB_0057 | sga:GALLO_0059 | rpsQ 30S ribosomal protein S17 |
| SGGB_0060 | SGPB_0058 | sga:GALLO_0060 | rplN 50S ribosomal protein L14 |
| SGGB_0062 | SGPB_0060 | sga:GALLO_0062 | rplE 50S ribosomal protein L5 |
| SGGB_0073 | SGPB_0071 | sga:GALLO_0073 | rpmJ 50S ribosomal protein L36 |
| SGGB_0074 | SGPB_0072 | sga:GALLO_0074 | rpsM 30S ribosomal protein S13 |
| SGGB_0077 | SGPB_0075 | sga:GALLO_0077 | rplQ 50S ribosomal protein L17 |
| SGGB_0122 | SGPB_0118 | sga:GALLO_0124 | groES chaperonin GroES |
| SGGB_0135 | SGPB_0131 | sga:GALLO_0138 | conserved hypothetical protein |
| SGGB_0210 | SGPB_0153 | sga:GALLO_0166 | hypothetical protein |
| SGGB_0231 | SGPB_0174 | sga:GALLO_0187 | lacF PTS system, lactose-specific IIA component |
| SGGB_0247 | SGPB_0191 | sga:GALLO_0205 | uppS undecaprenyl pyrophosphate synthetase |
| SGGB_0261 | SGPB_0205 | sga:GALLO_0219 | rpsO 30S ribosomal protein S15 |
| SGGB_0275 | SGPB_0218 | sga:GALLO_0233 | rplM 50S ribosomal protein L13 |
| SGGB_0276 | SGPB_0219 | sga:GALLO_0234 | rpsI 30S ribosomal protein S9 |
| SGGB_0319 | SGPB_0239 | sga:GALLO_0246 | truA tRNA pseudouridine synthase A |
| SGGB_0329 | SGPB_0253 | sga:GALLO_0301 | rpmB 50S ribosomal protein L28 |
| SGGB_0346 | SGPB_0270 | sga:GALLO_0318 | sufC Fe-S cluster assembly ATP-binding protein |
| SGGB_0349 | SGPB_0273 | sga:GALLO_0321 | nifU nitrogen fixation protein NifU and related proteins |
| SGGB_0364 | SGPB_0289 | sga:GALLO_0336 | acpB acyl carrier protein |
| SGGB_0370 | SGPB_0295 | sga:GALLO_0342 | fabZ 3R-hydroxymyristoyl ACP dehydrase |
| SGGB_0391 | SGPB_0321 | sga:GALLO_0363 | tRNA (guanine-N7-)-methyltransferase |
| SGGB_0399 | SGPB_0324 | sga:GALLO_0370 | conserved hypothetical protein |
| SGGB_0422 | SGPB_0352 | sga:GALLO_0393 | conserved hypothetical protein |
| SGGB_0462 | SGPB_0389 | sga:GALLO_0488 | gmk guanylate kinase |
| SGGB_0473 | SGPB_0400 | sga:GALLO_0499 | general stress protein 13 |
| SGGB_0480 | SGPB_0408 | sga:GALLO_0506 | conserved hypothetical protein |
| SGGB_0563 | SGPB_0468 | sga:GALLO_0595 | rhodanese-like domain-containing protein |
| SGGB_0571 | SGPB_0471 | sga:GALLO_0598 | conserved hypothetical protein |
| SGGB_0588 | SGPB_0488 | sga:GALLO_0616 | conserved hypothetical protein |
| SGGB_0594 | SGPB_0494 | sga:GALLO_0622 | folD bifunctional methylenetetrahydrofolate dehydrogenase (NADP+) / methenyltetrahydrofolate cyclohydrolase |
| SGGB_0603 | SGPB_0497 | sga:GALLO_0628 | xseB exodeoxyribonuclease VII small subunit |
| SGGB_0616 | SGPB_0510 | sga:GALLO_0641 | ArsR family transcriptional regulator |
| SGGB_0722 | SGPB_0619 | sga:GALLO_0741 | rpmE 50S ribosomal protein L31 |
| SGGB_0726 | SGPB_0622 | sga:GALLO_0744 | PTS-HPR phosphocarrier protein |
| SGGB_0727 | SGPB_0623 | sga:GALLO_0745 | ptsI phosphotransferase system, enzyme I, PtsI |
| SGGB_0733 | SGPB_0628 | sga:GALLO_0750 | udk uridine kinase |
| SGGB_0772 | SGPB_0649 | sga:GALLO_0787 | atpE F-type H+-transporting ATPase subunit c |
| SGGB_0774 | SGPB_0651 | sga:GALLO_0789 | atpF F-type H+-transporting ATPase subunit b |
| SGGB_0792 | SGPB_0669 | sga:GALLO_0807 | signal peptide containing protein |
| SGGB_0864 | SGPB_0752 | sga:GALLO_0879 | predicted membrane protein |
| SGGB_0959 | SGPB_0836 | sga:GALLO_0970 | pyrE orotate phosphoribosyltransferase |
| SGGB_0993 | SGPB_0864 | sga:GALLO_1003 | bglA.1 6-phospho-beta-glucosidase |
| SGGB_1001 | SGPB_0872 | sga:GALLO_1011 | conserved hypothetical protein |
| SGGB_1015 | SGPB_0888 | sga:GALLO_1025 | guaC GMP reductase |
| SGGB_1022 | SGPB_0894 | sga:GALLO_1032 | 4-oxalocrotonate tautomerase |
| SGGB_1151 | SGPB_1018 | sga:GALLO_1159 | spxA.1 transcriptional regulator Spx |
| SGGB_1250 | SGPB_1166 | sga:GALLO_1256 | predicted membrane protein |
| SGGB_1255 | SGPB_1171 | sga:GALLO_1261 | conserved hypothetical protein |
| SGGB_1317 | SGPB_1226 | sga:GALLO_1323 | rimM 16S rRNA processing protein RimM |
| SGGB_1318 | SGPB_1227 | sga:GALLO_1324 | conserved hypothetical protein |
| SGGB_1319 | SGPB_1228 | sga:GALLO_1325 | rpsP 30S ribosomal protein S16 |
| SGGB_1336 | SGPB_1263 | sga:GALLO_1341 | rpmA 50S ribosomal protein L27 |
| SGGB_1338 | SGPB_1265 | sga:GALLO_1343 | rplU LSU ribosomal protein L21 |
| SGGB_1354 | SGPB_1281 | sga:GALLO_1359 | rplT 50S ribosomal protein L20 |
| SGGB_1355 | SGPB_1282 | sga:GALLO_1360 | rpmI 50S ribosomal protein L35 |
| SGGB_1381 | SGPB_1306 | sga:GALLO_1387 | rpsU 30S ribosomal protein S21 |
| SGGB_1430 | SGPB_1341 | sga:GALLO_1438 | cas1 CRISPR-associated endonuclease Cas1 |
| SGGB_1450 | SGPB_1355 | sga:GALLO_1455 | predicted membrane protein |
| SGGB_1463 | SGPB_1367 | sga:GALLO_1467 | hisH glutamine amidotransferase |
| SGGB_1465 | SGPB_1369 | sga:GALLO_1469 | serB phosphoserine phosphatase |
| SGGB_1533 | SGPB_1432 | sga:GALLO_1537 | PspC domain-containing protein |
| SGGB_1536 | SGPB_1435 | sga:GALLO_1540 | conserved hypothetical protein |
| SGGB_1578 | SGPB_1462 | sga:GALLO_1579 | thrS threonyl-tRNA synthetase |
| SGGB_1645 | SGPB_1504 | sga:GALLO_1631 | putative metalloprotease |
| SGGB_1651 | SGPB_1508 | sga:GALLO_1637 | conserved hypothetical protein |
| SGGB_1653 | SGPB_1510 | sga:GALLO_1639 | conserved hypothetical protein |
| SGGB_1654 | SGPB_1511 | sga:GALLO_1640 | frr ribosome recycling factor |
| SGGB_1657 | SGPB_1514 | sga:GALLO_1643 | rplK 50S robosomal protein L11 |
| SGGB_1594 | SGPB_1670 | sga:GALLO_1692 | Tn916 ORF16 ATP/GTP-binding protein |
| SGGB_1597 | SGPB_1667 | sga:GALLO_1695 | Tn916 ORF19 protein |
| SGGB_1707 | SGPB_1535 | sga:GALLO_1720 | predicted membrane protein |
| SGGB_1713 | SGPB_1541 | sga:GALLO_1726 | conserved hypothetical protein |
| SGGB_1720 | SGPB_1548 | sga:GALLO_1733 | xthA exodeoxyribonuclease III |
| SGGB_1728 | SGPB_1551 | sga:GALLO_1741 | ArsC family transcription regulator |
| SGGB_1735 | SGPB_1558 | sga:GALLO_1748 | nrgB nitrogen regulatory protein P-II 1 |
| SGGB_1750 | SGPB_1573 | sga:GALLO_1763 | clpP ATP-dependent Clp protease, protease subunit |
| SGGB_1753 | SGPB_1574 | sga:GALLO_1766 | upp uracil phosphoribosyltransferase |
| SGGB_1785 | SGPB_1602 | sga:GALLO_1798 | trkH trk system potassium uptake protein TrkH |
| SGGB_1802 | SGPB_1632 | sga:GALLO_1815 | greA transcription elongation factor GreA |
| SGGB_1827 | SGPB_1647 | sga:GALLO_1840 | Rrf2 family transcriptional regulators |
| SGGB_1828 | SGPB_1646 | sga:GALLO_1841 | metI D-methionine transport system permease protein |
| SGGB_1860 | SGPB_1706 | sga:GALLO_1876 | codY transcriptional pleiotropic repressor |
| SGGB_1880 | SGPB_1727 | sga:GALLO_1896 | conserved hypothetical protein |
| SGGB_1884 | SGPB_1731 | sga:GALLO_1900 | comEB dCMP deaminase comEB |
| SGGB_1889 | SGPB_1734 | sga:GALLO_1905 | metal ion transporter, MIT family |
| SGGB_1894 | SGPB_1739 | sga:GALLO_1910 | rpsR 30S ribosomal protein S18 |
| SGGB_1900 | SGPB_1745 | sga:GALLO_1916 | bioB biotin synthetase |
| SGGB_1955 | SGPB_1804 | sga:GALLO_1972 | rpsN2 30S ribosomal protein S14 |
| SGGB_1982 | SGPB_1825 | sga:GALLO_1998 | rpsG 30S ribosomal protein S7 |
| SGGB_1984 | SGPB_1827 | sga:GALLO_2000 | purR purine operon repressor |
| SGGB_1995 | SGPB_1834 | sga:GALLO_2011 | enterocin A Immunity protein |
| SGGB_2025 | SGPB_1849 | sga:GALLO_2042 | hypothetical protein |
| SGGB_2036 | SGPB_1860 | sga:GALLO_2053 | dltC D-alanine-poly(phosphoribitol) ligase |
| SGGB_2064 | SGPB_1872 | sga:GALLO_2081 | argG argininosuccinate synthase |
| SGGB_2070 | SGPB_1876 | sga:GALLO_2086 | cynT carbonic anhydrase |
| SGGB_2086 | SGPB_1896 | sga:GALLO_2103 | tgt queuine tRNA-ribosyltransferase |
| SGGB_2214 | SGPB_1940 | sga:GALLO_2182 | nrdG anaerobic ribonucleoside-triphosphate reductase activating protein |
| SGGB_2221 | SGPB_1948 | sga:GALLO_2189 | conserved hypothetical protein |
| SGGB_2234 | SGPB_1961 | sga:GALLO_2201 | argR.2 transcriptional regulator of arginine metabolism |
| SGGB_2243 | SGPB_1971 | sga:GALLO_2210 | rpmF 50S ribosomal protein L32 |
| SGGB_2244 | SGPB_1972 | sga:GALLO_2211 | rpmG.2 50S ribosomal protein L33 |
| SGGB_2266 | SGPB_1992 | sga:GALLO_2233 | conserved hypothetical protein |
