# Supplementary material for: Sequencing and Comparative Genome Analysis of Two Pathogenic Streptococcus gallolyticus Subspecies: Genome Plasticity, Adaptation and Virulence
Source: PLoS One. 2011 May 25;6(5):e20519. doi: 10.1371/journal.pone.0020519 (PMC3102119; doi:10.1371/journal.pone.0020519)
Supplement: Table S3 — List of ATCC 43143 unique CDS not found in other sequenced Streptococci. A table listing the 99 S. gallolyticus ATCC 43143-specific proteins. (DOC) [file pone.0020519.s006.doc]

**Table S3. List of ATCC 43143 unique CDS not found in other sequenced Streptococci.** A table listing the 99 *S. gallolyticus* ATCC 43143-specific proteins.

| **Gene** | **Strand** | **Left** | **Right** | **Descriptions** |
| --- | --- | --- | --- | --- |
| SGGB_0048 | - | 61806 | 61931 | putative extracellular protein |
| SGGB_0142 | - | 151276 | 151620 | hypothetical protein |
| SGGB_0143 | + | 151031 | 151279 | hypothetical protein |
| SGGB_0144 | + | 151723 | 151974 | hypothetical protein |
| SGGB_0145 | - | 152348 | 152749 | hypothetical protein |
| SGGB_0146 | + | 152093 | 152368 | hypothetical protein |
| SGGB_0148 | + | 153161 | 153406 | hypothetical protein |
| SGGB_0149 | - | 153411 | 153524 | putative extracellular protein |
| SGGB_0160 | + | 161935 | 162090 | hypothetical protein |
| SGGB_0165 | + | 163597 | 163845 | YefM family antitoxin |
| SGGB_0166 | + | 163839 | 164117 | YoeB family toxin |
| SGGB_0167 | - | 164571 | 165380 | hypothetical protein |
| SGGB_0177 | - | 174806 | 175459 | hypothetical protein |
| SGGB_0181 | + | 178173 | 178457 | hypothetical protein |
| SGGB_0182 | - | 178821 | 179654 | hypothetical protein |
| SGGB_0183 | + | 178511 | 178837 | hypothetical protein |
| SGGB_0280 | - | 281975 | 282280 | dinJ2 addiction module antitoxin, RelB/DinJ family |
| SGGB_0282 | - | 283401 | 283628 | ICESt1 ORFB |
| SGGB_0286 | - | 288517 | 288744 | ICESt1 ORFF |
| SGGB_0287 | - | 288761 | 289753 | ICESt1 ORFG |
| SGGB_0288 | - | 289763 | 290317 | ICESt1 ORFH |
| SGGB_0289 | - | 290314 | 290550 | ICESt1 ORFI |
| SGGB_0295 | - | 295139 | 295375 | hypothetical protein |
| SGGB_0297 | - | 295783 | 296055 | dinJ3 addiction module antitoxin, RelB/DinJ family |
| SGGB_0303 | - | 301809 | 303359 | dcm1 DNA (cytosine-5-)-methyltransferase |
| SGGB_0304 | + | 303553 | 304014 | putative phage transcriptional regulator |
| SGGB_0305 | + | 304014 | 305234 | conserved hypothetical protein |
| SGGB_0306 | + | 305251 | 306186 | hypothetical protein |
| SGGB_0307 | + | 306179 | 307843 | conserved hypothetical protein |
| SGGB_0484 | - | 504806 | 506023 | AraC family transcriptional regulator |
| SGGB_0502 | - | 525076 | 525867 | signal peptide containing protein |
| SGGB_0530 | + | 554583 | 555041 | hypothetical protein |
| SGGB_0538 | + | 559763 | 560251 | conserved hypothetical protein |
| SGGB_0539 | - | 561059 | 561196 | hypothetical protein |
| SGGB_0541 | - | 561233 | 561382 | hypothetical protein |
| SGGB_0544 | + | 562368 | 565460 | Cna protein B-type domain-containing protein (LPXTG motif) |
| SGGB_0567 | - | 584409 | 584849 | hypothetical protein |
| SGGB_0598 | + | 618070 | 620151 | Bsp-like repeat containing hypothetical protein |
| SGGB_0641 | + | 662466 | 662993 | hypothetical protein |
| SGGB_0642 | - | 663081 | 663203 | hypothetical protein |
| SGGB_0671 | + | 691149 | 691514 | putative transcriptional regulator |
| SGGB_0724 | - | 749880 | 750008 | hypothetical protein |
| SGGB_0725 | + | 750112 | 750249 | putative extracellular protein |
| SGGB_0771 | - | 806579 | 806704 | putative extracellular protein |
| SGGB_0822 | + | 857815 | 857991 | predicted membrane protein |
| SGGB_0829 | - | 866381 | 867238 | LysR family transcriptional regulator |
| SGGB_0863 | - | 903755 | 904333 | signal peptide containing protein |
| SGGB_0903 | + | 938970 | 940403 | Lanthionine synthetase C-like protein |
| SGGB_0911 | + | 948973 | 950598 | conserved hypothetical protein |
| SGGB_0935 | + | 973866 | 975839 | cpsH glycosyl transferase |
| SGGB_0942 | + | 981807 | 982949 | cpsO polysaccharide pyruvyl transferase |
| SGGB_0954 | + | 990502 | 990615 | hypothetical protein |
| SGGB_1061 | + | 1110198 | 1110377 | hypothetical protein |
| SGGB_1078 | - | 1126662 | 1126781 | predicted membrane protein |
| SGGB_1091 | - | 1137634 | 1137822 | predicted membrane protein |
| SGGB_1092 | - | 1137827 | 1137976 | predicted membrane protein |
| SGGB_1128 | - | 1176911 | 1177027 | hypothetical protein |
| SGGB_1238 | - | 1284841 | 1285020 | putative extracellular protein |
| SGGB_1263 | - | 1308396 | 1308542 | hypothetical protein |
| SGGB_1327 | - | 1369000 | 1369137 | hypothetical protein |
| SGGB_1363 | - | 1405283 | 1406380 | conserved hypothetical protein |
| SGGB_1364 | - | 1406346 | 1406624 | signal peptide containing protein |
| SGGB_1376 | - | 1421200 | 1421328 | endA deoxyribonuclease I |
| SGGB_1404 | - | 1452696 | 1452824 | predicted membrane protein |
| SGGB_1417 | - | 1466336 | 1466929 | transporter protein |
| SGGB_1418 | - | 1466914 | 1467267 | predicted membrane protein |
| SGGB_1433 | - | 1484755 | 1484904 | putative extracellular protein |
| SGGB_1518 | - | 1576389 | 1576580 | hypothetical protein |
| SGGB_1548 | - | 1603851 | 1603979 | hypothetical protein |
| SGGB_1557 | - | 1610968 | 1611084 | predicted membrane protein |
| SGGB_1662 | - | 1728431 | 1728727 | hypothetical protein |
| SGGB_1676 | - | 1740611 | 1740910 | hypothetical protein |
| SGGB_1677 | - | 1741027 | 1741311 | hypothetical protein |
| SGGB_1694 | - | 1752685 | 1752819 | hypothetical protein |
| SGGB_1779 | - | 1831342 | 1831455 | putative extracellular protein |
| SGGB_1910 | + | 1961883 | 1962125 | hypothetical protein |
| SGGB_1965 | - | 2016249 | 2016767 | rpiB ribose 5-phosphate isomerase B |
| SGGB_1967 | - | 2017978 | 2018097 | putative extracellular protein |
| SGGB_1996 | - | 2049644 | 2049793 | putative bacteriocin |
| SGGB_2004 | - | 2055302 | 2055430 | putative bacteriocin |
| SGGB_2040 | - | 2098511 | 2098762 | predicted membrane protein |
| SGGB_2063 | - | 2119136 | 2119261 | hypothetical protein |
| SGGB_2066 | - | 2122099 | 2122278 | predicted membrane protein |
| SGGB_2146 | + | 2203655 | 2203804 | hypothetical protein |
| SGGB_2154 | - | 2211727 | 2212092 | conserved hypothetical protein |
| SGGB_2169 | - | 2226298 | 2229714 | cell surface-associated protein autolysin AtlA |
| SGGB_2170 | - | 2229912 | 2232347 | cell wall surface protein (LPXTG motif) |
| SGGB_2176 | - | 2238233 | 2238364 | putative extracellular protein |
| SGGB_2177 | - | 2238427 | 2244702 | conserved hypothetical protein |
| SGGB_2187 | - | 2252536 | 2252811 | hypothetical protein |
| SGGB_2188 | - | 2252768 | 2253100 | hypothetical protein |
| SGGB_2189 | - | 2253116 | 2253406 | hypothetical protein |
| SGGB_2190 | - | 2253430 | 2253660 | hypothetical protein |
| SGGB_2193 | - | 2254702 | 2254857 | putative extracellular protein |
| SGGB_2195 | - | 2255083 | 2255256 | hypothetical protein |
| SGGB_2197 | - | 2256904 | 2257302 | hypothetical protein |
| SGGB_2198 | - | 2257469 | 2258218 | hypothetical protein |
| SGGB_2201 | - | 2259967 | 2260191 | hypothetical protein |
| SGGB_2226 | - | 2290880 | 2291053 | predicted membrane protein |
