# Supplementary material for: Sequencing and Comparative Genome Analysis of Two Pathogenic Streptococcus gallolyticus Subspecies: Genome Plasticity, Adaptation and Virulence
Source: PLoS One. 2011 May 25;6(5):e20519. doi: 10.1371/journal.pone.0020519 (PMC3102119; doi:10.1371/journal.pone.0020519)
Supplement: Table S4 — List of ATCC 43144 unique CDS not found in other sequenced Streptococci. A table listing the 116 S. pasteurianus ATCC 43144-specific proteins. (DOC) [file pone.0020519.s007.doc]

**Table S4. List of ATCC 43144 unique CDS not found in other sequenced Streptococci.** A table listing the 116 S. pasteurianus ATCC 43144-specific proteins.

| **Gene** | **Strand** | **Left** | **Right** | **Descriptions** |
| --- | --- | --- | --- | --- |
| SGPB_0101 | - | 104132 | 104641 | GNAT family acetyltransferase |
| SGPB_0127 | - | 129832 | 130059 | conserved hypothetical protein |
| SGPB_0140 | + | 144814 | 144984 | hypothetical protein |
| SGPB_0231 | - | 244626 | 244745 | putative extracellular protein |
| SGPB_0250 | + | 263051 | 264046 | scrR.1 LacI family transcriptional regulator, sucrose operon repressor |
| SGPB_0282 | - | 305341 | 305661 | signal peptide containing protein |
| SGPB_0283 | - | 305852 | 305992 | hypothetical protein |
| SGPB_0315 | + | 335164 | 335511 | haloacid dehalogenase-like hydrolase |
| SGPB_0406 | - | 426785 | 426901 | predicted membrane protein |
| SGPB_0451 | + | 474340 | 474555 | hypothetical protein |
| SGPB_0454 | + | 476048 | 476425 | ytrA GntR family transcriptional regulator |
| SGPB_0456 | + | 477290 | 477958 | predicted membrane protein |
| SGPB_0460 | - | 481747 | 482496 | predicted membrane protein |
| SGPB_0515 | - | 536391 | 536987 | phage transcriptional regulator |
| SGPB_0516 | + | 537139 | 537324 | conserved hypothetical protein |
| SGPB_0536 | + | 557766 | 558626 | AraC family transcriptional regulator |
| SGPB_0574 | + | 594715 | 595104 | hypothetical protein |
| SGPB_0621 | - | 646946 | 647086 | hypothetical protein |
| SGPB_0680 | + | 715383 | 718106 | cell wall surface protein (LPXTG motif) |
| SGPB_0681 | + | 718275 | 718562 | predicted membrane protein |
| SGPB_0682 | + | 718595 | 718789 | csbD.1 CsbD-like protein |
| SGPB_0699 | + | 733164 | 733340 | predicted membrane protein |
| SGPB_0700 | + | 733678 | 735483 | kdpA high affinity K+-transporting ATPase ATPase A chain |
| SGPB_0702 | + | 737612 | 738214 | kdpC high affinity K+-transporting ATPase ATPase C chain |
| SGPB_0703 | + | 738665 | 741364 | kdpD two-component system, OmpR family, sensor histidine kinase KdpD |
| SGPB_0705 | + | 742439 | 742588 | hypothetical protein |
| SGPB_0706 | + | 742980 | 743156 | hypothetical protein |
| SGPB_0718 | + | 753318 | 754082 | RpiR family transcriptional regulator, glv operon transcriptional regulator |
| SGPB_0734 | + | 772175 | 773563 | kdgT 2-keto-3-deoxygluconate permease |
| SGPB_0737 | - | 776098 | 777078 | amidohydrolases |
| SGPB_0749 | + | 790120 | 790236 | putative extracellular protein |
| SGPB_0751 | - | 791422 | 791964 | predicted lipoprotein |
| SGPB_0787 | + | 820635 | 820964 | two-membrane-helix-type ion channel |
| SGPB_0814 | + | 849393 | 850619 | cpsF aminotransferase family protein |
| SGPB_0816 | + | 851327 | 852097 | cpsH glycosyl transferase family 2 |
| SGPB_0817 | + | 852098 | 853000 | cpsI glycosyltransferase |
| SGPB_0818 | + | 853006 | 854037 | cpsJ glycosyl transferase family 1 |
| SGPB_0819 | + | 854043 | 854675 | cpsK hypothetical protein |
| SGPB_0822 | + | 856852 | 858285 | wzx polysaccharide flippase transporter |
| SGPB_0890 | + | 928984 | 929103 | hypothetical protein |
| SGPB_0895 | - | 933173 | 933358 | conserved hypothetical protein |
| SGPB_0912 | + | 953209 | 953469 | hypothetical protein |
| SGPB_0913 | + | 953575 | 953898 | hypothetical protein |
| SGPB_0914 | + | 954004 | 955281 | predicted membrane protein |
| SGPB_0915 | + | 955370 | 955759 | hypothetical protein |
| SGPB_0916 | + | 955900 | 956364 | hypothetical protein |
| SGPB_0925 | + | 965855 | 966688 | hypothetical protein |
| SGPB_0937 | - | 978157 | 978276 | predicted membrane protein |
| SGPB_0942 | - | 981060 | 981587 | satE secretion and acid tolerance protein SatE |
| SGPB_0951 | - | 988750 | 990369 | lantibiotic ABC transporter permease protein |
| SGPB_0974 | - | 1019542 | 1020282 | predicted membrane protein |
| SGPB_0975 | - | 1020275 | 1020823 | ECF subfamily RNA polymerase sigma-24 factor |
| SGPB_0977 | - | 1023056 | 1023199 | signal peptide containing protein |
| SGPB_1046 | - | 1090891 | 1091013 | putative extracellular protein |
| SGPB_1073 | - | 1115358 | 1115657 | predicted lipoprotein |
| SGPB_1074 | - | 1115664 | 1117340 | carboxylesterase type B |
| SGPB_1095 | - | 1137689 | 1137868 | putative extracellular protein |
| SGPB_1100 | - | 1141111 | 1141827 | nsuI nisin immunity protein |
| SGPB_1101 | - | 1141829 | 1143106 | nsuC lanthionine synthetase C-like protein |
| SGPB_1104 | - | 1144887 | 1147862 | nsuB lantibiotic dehydratase |
| SGPB_1105 | - | 1148103 | 1148270 | nsuA nisin U lantibiotic |
| SGPB_1115 | - | 1157377 | 1158519 | ICESt1 ORFD ATP/GTP-binding protein (Tn916 ORF16 related) |
| SGPB_1116 | - | 1158520 | 1159308 | ICESt1 ORFV2 |
| SGPB_1123 | - | 1163972 | 1164547 | TetR family transcriptional regulator |
| SGPB_1124 | + | 1164645 | 1165499 | conserved hypothetical protein |
| SGPB_1148 | - | 1197373 | 1197534 | conserved hypothetical protein |
| SGPB_1156 | - | 1202959 | 1204065 | predicted lipoprotein |
| SGPB_1215 | - | 1258928 | 1259044 | predicted membrane protein |
| SGPB_1217 | - | 1259393 | 1259617 | predicted membrane protein |
| SGPB_1230 | - | 1273865 | 1274689 | filamentation induced by cAMP protein Fic |
| SGPB_1231 | - | 1274808 | 1275032 | conserved hypothetical protein |
| SGPB_1233 | - | 1275924 | 1276061 | hypothetical protein |
| SGPB_1235 | - | 1276890 | 1277318 | predicted membrane protein |
| SGPB_1240 | - | 1284649 | 1284792 | hypothetical protein |
| SGPB_1249 | - | 1291851 | 1291967 | predicted membrane protein |
| SGPB_1290 | - | 1332875 | 1334215 | signal peptide containing protein |
| SGPB_1331 | - | 1384087 | 1384914 | SIS (Sugar ISomerase) domain containing transcriptional regulator |
| SGPB_1426 | + | 1488516 | 1488653 | putative extracellular protein |
| SGPB_1456 | - | 1516443 | 1516559 | predicted membrane protein |
| SGPB_1483 | - | 1543600 | 1546173 | levR PRD domain/Sigma-54 interaction domain containing transcriptional regulator |
| SGPB_1488 | - | 1548958 | 1549095 | putative bacteriocin |
| SGPB_1523 | - | 1585247 | 1588006 | ypcD endo-beta-N-acetylglucosaminidase |
| SGPB_1533 | - | 1602287 | 1602403 | hypothetical protein |
| SGPB_1586 | - | 1648544 | 1648681 | predicted membrane protein |
| SGPB_1613 | - | 1673226 | 1673441 | hypothetical protein |
| SGPB_1614 | - | 1673510 | 1673995 | conserved hypothetical protein |
| SGPB_1616 | - | 1674294 | 1674428 | predicted membrane protein |
| SGPB_1618 | - | 1675066 | 1675536 | conserved hypothetical protein |
| SGPB_1619 | - | 1675533 | 1675775 | conserved hypothetical protein |
| SGPB_1627 | - | 1682819 | 1684093 | lacY galactoside permease |
| SGPB_1661 | + | 1720101 | 1723145 | Cna protein B-type domain-containing protein (LPXTG motif) |
| SGPB_1664 | + | 1724202 | 1724636 | predicted membrane protein |
| SGPB_1690 | - | 1750881 | 1752914 | conserved hypothetical protein |
| SGPB_1722 | + | 1782408 | 1782593 | hypothetical protein |
| SGPB_1730 | - | 1790101 | 1790280 | hypothetical protein |
| SGPB_1742 | - | 1799959 | 1800216 | putative extracellular protein |
| SGPB_1751 | + | 1809275 | 1809517 | hypothetical protein |
| SGPB_1758 | - | 1816215 | 1816664 | predicted membrane protein |
| SGPB_1760 | - | 1817718 | 1819298 | ramA alpha-L-rhamnosidase |
| SGPB_1761 | - | 1819303 | 1820562 | major facilitator superfamily protein |
| SGPB_1762 | - | 1820559 | 1821920 | major facilitator superfamily protein |
| SGPB_1763 | + | 1822094 | 1823140 | AraC family transcriptional regulator |
| SGPB_1787 | + | 1848635 | 1850545 | transcriptional antiterminator |
| SGPB_1821 | - | 1883366 | 1883482 | hypothetical protein |
| SGPB_1833 | - | 1895371 | 1895598 | putative bacteriocin |
| SGPB_1841 | - | 1901392 | 1901511 | putative extracellular protein |
| SGPB_1864 | - | 1928805 | 1929056 | predicted membrane protein |
| SGPB_1882 | - | 1944899 | 1945030 | putative extracellular protein |
| SGPB_1895 | + | 1956172 | 1956336 | putative extracellular protein |
| SGPB_1969 | - | 2039799 | 2040347 | conserved hypothetical protein |
| SGPB_1973 | - | 2043052 | 2044026 | SEFIR domain-containing protein |
| SGPB_1976 | - | 2047023 | 2047976 | hypothetical protein |
| SGPB_1977 | + | 2048269 | 2048703 | conserved hypothetical protein |
| SGPB_2011 | - | 2081361 | 2081684 | Phage regulatory protein Rha |
| SGPB_2013 | + | 2082092 | 2082724 | putative transcriptional regulator |
| SGPB_2014 | + | 2083154 | 2083366 | predicted membrane protein |
