# Supplementary material for: Sequencing and Comparative Genome Analysis of Two Pathogenic Streptococcus gallolyticus Subspecies: Genome Plasticity, Adaptation and Virulence
Source: PLoS One. 2011 May 25;6(5):e20519. doi: 10.1371/journal.pone.0020519 (PMC3102119; doi:10.1371/journal.pone.0020519)
Supplement: Table S5 — List of regions of genomic plasticity (RGPs) in the ATCC 43143 genome. A table listing the identified RGPs in S. gallolyticus ATCC 43143. (DOC) [file pone.0020519.s008.doc]

**Table S5. List of regions of genomic plasticity (RGPs) in the ATCC 43143 genome.** A table listing the identified RGPs in *S. gallolyticus* ATCC 43143.

| **RGP No.** | **ATCC 43143 Locus ID** | **UCN34**  **% Homology ^a)^** | **ATCC 43144**  **% Homology ^b)^** | **Descriptions** | **No. of TMD** | **Signal Peptide Prediction** | **Localization Prediction** |
| --- | --- | --- | --- | --- | --- | --- | --- |
| 1 | SGGB_0136 | 100 | 0 | NADPH-quinone reductase | 0 |  | Unknown |
| 1 | SGGB_0137 | 100 | 0 | cinnamoyl ester hydrolase | 0 |  | Unknown |
| 1 | SGGB_0138 | 90 | 0 | DNA-damage-inducible protein J | 0 |  | Unknown |
| 1 | SGGB_0139 | 51 | 0 | predicted lipoprotein | 0 | Lipoprotein signal peptide | Unknown |
| 1 | SGGB_0140 | 56 | 0 | ICESt1 ORFQ | 0 |  | Cytoplasmic |
| 1 | SGGB_0141 | 59 | 0 | ICESt1 APR2 Cro/CI family transcriptional regulator | 0 |  | Cytoplasmic |
| 1 | SGGB_0142 | 0 | 0 | hypothetical protein | 0 |  | Cytoplasmic |
| 1 | SGGB_0143 | 0 | 0 | hypothetical protein | 0 |  | Cytoplasmic |
| 1 | SGGB_0144 | 0 | 0 | hypothetical protein | 0 |  | Unknown |
| 1 | SGGB_0145 | 0 | 0 | hypothetical protein | 0 |  | Unknown |
| 1 | SGGB_0146 | 0 | 0 | hypothetical protein | 0 |  | Cytoplasmic |
| 1 | SGGB_0147 | 0 | 0 | conserved hypothetical protein | 0 |  | Cytoplasmic |
| 1 | SGGB_0148 | 0 | 0 | hypothetical protein | 0 |  | Cytoplasmic |
| 1 | SGGB_0149 | 0 | 0 | putative extracellular protein | 0 |  | Extracellular |
| 1 | SGGB_0150 | 97 | 0 | hypothetical protein | 0 |  | Unknown |
| 1 | SGGB_0151 | 75 | 0 | conserved hypothetical protein | 0 |  | Cytoplasmic |
| 1 | SGGB_0152 | 90 | 0 | thioredoxin/glutaredoxin-like signal peptide containing protein | 1 |  | Unknown |
| 1 | SGGB_0153 | 94 | 0 | conserved hypothetical protein | 0 |  | Unknown |
| 1 | SGGB_0154 | 97 | 0 | cell wall surface protein (LPXTG motif) | 1 |  | Cellwall |
| 1 | SGGB_0155 | 100 | 0 | ICESt1 ORFM | 0 |  | Cytoplasmic |
| 1 | SGGB_0156 | 99 | 0 | ICESt1 ORFL | 0 |  | Unknown |
| 1 | SGGB_0157 | 100 | 0 | ICESt1 ORFK, FtsK/SpoIIIE family protein | 2 | Signal peptide | Unknown |
| 1 | SGGB_0158 | 100 | 0 | ICESt1 ORFJ phage replication initiation factor | 0 |  | Cytoplasmic |
| 1 | SGGB_0159 | 98 | 0 | conserved hypothetical protein | 0 |  | Unknown |
| 1 | SGGB_0160 | 0 | 0 | hypothetical protein | 0 |  | Unknown |
| 1 | SGGB_0161 | 100 | 0 | ICESt1 ORFH | 0 |  | Unknown |
| 1 | SGGB_0162 | 78 | 0 | antirestriction (ArdA) proteins | 0 |  | Cytoplasmic |
| 1 | SGGB_0163 | 75 | 0 | hypothetical protein | 0 |  | Cytoplasmic |
| 1 | SGGB_0164 | 90 | 0 | hypothetical protein | 0 |  | Cytoplasmic |
| 1 | SGGB_0165 | 0 | 0 | YefM family antitoxin | 0 |  | Unknown |
| 1 | SGGB_0166 | 0 | 0 | YoeB family toxin | 0 |  | Unknown |
| 1 | SGGB_0167 | 0 | 0 | hypothetical protein | 6 |  | CytoplasmicMembrane |
| 1 | SGGB_0168 | 96 | 0 | conserved hypothetical protein | 0 |  | Cytoplasmic |
| 1 | SGGB_0169 | 97 | 0 | ICESt1 ORFG | 1 |  | Unknown |
| 1 | SGGB_0170 | 74 | 0 | ICESt1 ORFF | 2 |  | CytoplasmicMembrane |
| 1 | SGGB_0171 | 85 | 0 | ICESt1 ORFE | 2 |  | Cytoplasmic |
| 1 | SGGB_0172 | 97 | 0 | predicted membrane protein | 3 | Signal peptide | CytoplasmicMembrane |
| 1 | SGGB_0173 | 99 | 0 | ICESt1 ORFD ATP/GTP-binding protein | 0 |  | Cytoplasmic |
| 1 | SGGB_0176 | 88 | 0 | signal peptide containing protein | 1 | Signal peptide | Unknown |
| 1 | SGGB_0177 | 0 | 0 | hypothetical protein | 4 |  | CytoplasmicMembrane |
| 1 | SGGB_0178 | 94 | 61 | sortase A | 1 | Signal peptide | Unknown |
| 1 | SGGB_0179 | 93 | 0 | predicted lipoprotein | 1 | Lipoprotein signal peptide | Unknown |
| 1 | SGGB_0180 | 94 | 0 | cell wall surface protein (LPXTG motif) | 1 | Signal peptide | Cellwall |
| 1 | SGGB_0181 | 0 | 0 | hypothetical protein | 0 |  | Cytoplasmic |
| 1 | SGGB_0182 | 0 | 0 | hypothetical protein | 0 |  | Unknown |
| 1 | SGGB_0183 | 0 | 0 | hypothetical protein | 0 |  | Cytoplasmic |
| 1 | SGGB_0184 | 0 | 0 | conserved hypothetical protein | 0 |  | Cytoplasmic |
| 1 | SGGB_0185 | 0 | 0 | phage integrase family recombinase | 0 |  | Unknown |
| 2 | SGGB_0277 | 0 | 73 | phage integrase family recombinase | 0 |  | Unknown |
| 2 | SGGB_0278 | 0 | 63 | phage DNA packaging protein Nu1 | 0 |  | Cytoplasmic |
| 2 | SGGB_0279 | 53 | 0 | addiction module toxin, PemK family | 0 |  | Cytoplasmic |
| 2 | SGGB_0280 | 0 | 0 | addiction module antitoxin, RelB/DinJ family | 0 |  | Cytoplasmic |
| 2 | SGGB_0281 | 0 | 0 | CHAP domain containing protein | 1 | Signal peptide | Unknown |
| 2 | SGGB_0282 | 0 | 0 | ICESt1 ORFB | 0 |  | Unknown |
| 2 | SGGB_0283 | 36 | 0 | ICESt1 ORFC | 9 |  | CytoplasmicMembrane |
| 2 | SGGB_0284 | 61 | 0 | ICESt1 ORFD ATP/GTP-binding protein | 0 |  | Cytoplasmic |
| 2 | SGGB_0285 | 60 | 0 | ICESt1 ORFE | 1 |  | CytoplasmicMembrane |
| 2 | SGGB_0286 | 0 | 0 | ICESt1 ORFF | 2 |  | Unknown |
| 2 | SGGB_0287 | 0 | 0 | ICESt1 ORFG | 1 | Signal peptide | Unknown |
| 2 | SGGB_0288 | 0 | 0 | ICESt1 ORFH | 0 |  | Cytoplasmic |
| 2 | SGGB_0289 | 0 | 0 | ICESt1 ORFI | 0 |  | Cytoplasmic |
| 2 | SGGB_0290 | 0 | 0 | conserved hypothetical protein | 0 |  | Unknown |
| 2 | SGGB_0291 | 0 | 0 | ICESt1 ORFJ phage replication initiation factor | 0 |  | Cytoplasmic |
| 2 | SGGB_0292 | 35 | 0 | ICESt1 ORFK, FtsK/SpoIIIE family protein | 3 |  | Cytoplasmic |
| 2 | SGGB_0293 | 0 | 0 | conserved hypothetical protein | 0 |  | Unknown |
| 2 | SGGB_0294 | 0 | 0 | conserved hypothetical protein | 0 |  | Cytoplasmic |
| 2 | SGGB_0295 | 0 | 0 | hypothetical protein | 0 |  | Unknown |
| 2 | SGGB_0296 | 53 | 0 | addiction module toxin, RelE/StbE family | 0 |  | Cytoplasmic |
| 2 | SGGB_0297 | 0 | 0 | addiction module antitoxin, RelB/DinJ family | 0 |  | Unknown |
| 2 | SGGB_0298 | 0 | 0 | phosphatidylethanolamine-binding protein | 0 |  | Cytoplasmic |
| 2 | SGGB_0299 | 47 | 0 | ICESt1 APR2 Cro/CI family transcriptional regulator | 0 |  | Cytoplasmic |
| 2 | SGGB_0300 | 38 | 0 | ICESt1 ORFQ | 0 |  | Unknown |
| 2 | SGGB_0301 | 0 | 0 | KAP family NTPases | 2 |  | Cytoplasmic |
| 2 | SGGB_0302 | 39 | 0 | predicted lipoprotein | 0 | Lipoprotein signal peptide | Unknown |
| 2 | SGGB_0303 | 0 | 0 | DNA (cytosine-5-)-methyltransferase | 0 |  | Cytoplasmic |
| 2 | SGGB_0304 | 0 | 0 | putative phage transcriptional regulator | 0 |  | Unknown |
| 2 | SGGB_0305 | 0 | 0 | conserved hypothetical protein | 0 |  | Cytoplasmic |
| 2 | SGGB_0306 | 0 | 0 | hypothetical protein | 0 |  | Unknown |
| 2 | SGGB_0307 | 0 | 0 | conserved hypothetical protein | 0 |  | Unknown |
| 3 | SGGB_0519 | 100 | 0 | WXG100 eSAT-6 secretion system EsxA | 0 |  | Unknown |
| 3 | SGGB_0520 | 99 | 0 | WXG100 eSAT-6 protein secretion system EssA, signal peptide containing | 2 | Signal peptide | Unknown |
| 3 | SGGB_0521 | 99 | 0 | Ubiquitin-binding YukD-like protein EsaB | 0 |  | Cytoplasmic |
| 3 | SGGB_0522 | 100 | 0 | WXG100 eSAT-6 protein secretion system EssB | 1 |  | Unknown |
| 3 | SGGB_0523 | 100 | 0 | DNA segregation ATPase FtsK/SpoIIIE, S-DNA-T family | 1 |  | Cytoplasmic |
| 3 | SGGB_0524 | 100 | 0 | WXG100 eSAT-6 secretion system EsaA, signal peptide containing | 2 |  | Cellwall |
| 3 | SGGB_0525 | 99 | 0 | WXG100 eSAT-6 secretion system EsaC | 0 |  | Unknown |
| 3 | SGGB_0526 | 100 | 0 | hypothetical protein | 0 |  | Unknown |
| 3 | SGGB_0527 | 100 | 0 | hypothetical protein | 0 |  | Cytoplasmic |
| 3 | SGGB_0528 | 96 | 0 | transposase, Gram-positive bacteria | 0 |  | Unknown |
| 3 | SGGB_0529 | 81 | 0 | predicted membrane protein | 6 |  | CytoplasmicMembrane |
| 3 | SGGB_0530 | 0 | 0 | hypothetical protein | 0 |  | Cytoplasmic |
| 3 | SGGB_0531 | 99 | 0 | conserved hypothetical protein | 0 |  | Cytoplasmic |
| 3 | SGGB_0532 | 99 | 0 | predicted membrane protein | 1 |  | Unknown |
| 3 | SGGB_0533 | 100 | 0 | predicted membrane protein | 6 |  | CytoplasmicMembrane |
| 3 | SGGB_0534 | 99 | 0 | predicted membrane protein | 6 |  | CytoplasmicMembrane |
| 3 | SGGB_0535 | 99 | 0 | hypothetical protein | 0 |  | Unknown |
| 3 | SGGB_0536 | 99 | 0 | hypothetical protein | 0 |  | Unknown |
| 3 | SGGB_0537 | 99 | 0 | predicted lipoprotein | 6 |  | CytoplasmicMembrane |
| 3 | SGGB_0538 | 0 | 0 | conserved hypothetical protein | 3 |  | CytoplasmicMembrane |
| 3 | SGGB_0539 | 0 | 0 | hypothetical protein | 0 |  | Unknown |
| 3 | SGGB_0540 | 98 | 0 | CAAX amino terminal protease family protein | 7 |  | CytoplasmicMembrane |
| 3 | SGGB_0541 | 0 | 0 | hypothetical protein | 0 |  | Unknown |
| 3 | SGGB_0542 | 97 | 0 | hypothetical protein | 0 |  | Cytoplasmic |
| 3 | SGGB_0543 | 98 | 0 | TetR family transcriptional regulator | 0 |  | Cytoplasmic |
| 3 | SGGB_0544 | 0 | 0 | Cna protein B-type domain-containing protein (LPXTG motif) | 2 | Signal peptide | Cellwall |
| 3 | SGGB_0545 | 99 | 0 | predicted membrane protein | 3 |  | CytoplasmicMembrane |
| 3 | SGGB_0546 | 100 | 0 | conserved hypothetical protein | 0 |  | Unknown |
| 3 | SGGB_0547 | 99 | 0 | TraX family protein | 8 |  | CytoplasmicMembrane |
| 3 | SGGB_0548 | 99 | 97 | conserved hypothetical protein | 10 |  | CytoplasmicMembrane |
| 3 | SGGB_0549 | 98 | 0 | isochorismate pyruvate-lyase | 0 |  | Cytoplasmic |
| 3 | SGGB_0550 | 100 | 0 | anthranilate synthase component I | 0 |  | Cytoplasmic |
| 3 | SGGB_0551 | 100 | 0 | anthranilate synthase component II | 0 |  | Cytoplasmic |
| 3 | SGGB_0552 | 97 | 0 | anthranilate phosphoribosyltransferase | 0 |  | Unknown |
| 3 | SGGB_0553 | 98 | 0 | indole-3-glycerol phosphate synthase | 0 |  | Cytoplasmic |
| 3 | SGGB_0554 | 99 | 0 | phosphoribosylanthranilate isomerase | 0 |  | Unknown |
| 3 | SGGB_0555 | 100 | 0 | tryptophan synthase beta chain | 0 |  | Cytoplasmic |
| 3 | SGGB_0556 | 100 | 0 | tryptophan synthase alpha chain | 0 |  | Unknown |
| 3 | SGGB_0557 | 99 | 99 | manganese transport protein | 10 |  | CytoplasmicMembrane |
| 3 | SGGB_0560 | 100 | 99 | Dps-like peroxide resistance protein Dpr | 0 |  | Unknown |
| 3 | SGGB_0561 | 100 | 100 | conserved hypothetical protein | 0 |  | Cytoplasmic |
| 3 | SGGB_0562 | 100 | 100 | glucokinase | 0 |  | Cytoplasmic |
| 3 | SGGB_0563 | 100 | 100 | rhodanese-like domain-containing protein | 1 |  | Cytoplasmic |
| 3 | SGGB_0564 | 99 | 96 | conserved hypothetical protein | 1 |  | Unknown |
| 3 | SGGB_0567 | 0 | 0 | hypothetical protein | 0 |  | Unknown |
| 4 | SGGB_0932 | 99 | 0 | glycosyltransferase in exopolysaccharide biosynthesis | 5 |  | CytoplasmicMembrane |
| 4 | SGGB_0933 | 0 | 0 | glycosyltransferase in exopolysaccharide biosynthesis | 0 |  | Unknown |
| 4 | SGGB_0934 | 0 | 0 | glycosyltransferase in exopolysaccharide biosynthesis | 0 |  | Cytoplasmic |
| 4 | SGGB_0935 | 0 | 0 | glycosyl transferase | 0 |  | Unknown |
| 4 | SGGB_0936 | 0 | 0 | glycosyl transferase family 1 | 0 |  | Unknown |
| 4 | SGGB_0937 | 0 | 0 | glycosyl transferase family 2 | 0 |  | Unknown |
| 4 | SGGB_0940 | 0 | 0 | glycosyltransferase in exopolysaccharide biosynthesis | 0 |  | Cytoplasmic |
| 4 | SGGB_0941 | 40 | 0 | polysaccharide flippase transporter | 10 |  | CytoplasmicMembrane |
| 4 | SGGB_0942 | 0 | 0 | polysaccharide pyruvyl transferase | 0 |  | Cytoplasmic |
| 4 | SGGB_0943 | 0 | 0 | glycosyl transferase family 2 | 0 |  | Unknown |
| 4 | SGGB_0944 | 31 | 31 | short-chain dehydrogenase/reductase SDR | 0 |  | Cytoplasmic |
| 4 | SGGB_0945 | 0 | 0 | IS4 family transposase | 0 |  | Cytoplasmic |
| 4 | SGGB_0948 | 0 | 0 | PemK family toxin | 0 |  | Unknown |
| 4 | SGGB_0949 | 0 | 0 | MazE/ChpS family antitoxin | 0 |  | Cytoplasmic |
| 4 | SGGB_0950 | 98 | 96 | conserved hypothetical protein | 0 |  | Cytoplasmic |
| 4 | SGGB_0953 | 100 | 0 | IS861, transposase OrfB | 0 |  | Unknown |
| 4 | SGGB_0954 | 0 | 0 | hypothetical protein | 0 |  | Unknown |
| 5 | SGGB_1041 | 100 | 0 | SAM-dependent methyltransferase | 0 |  | Cytoplasmic |
| 5 | SGGB_1042 | 98 | 0 | UDP-glucoronosyl and UDP-glucosyl transferase | 2 |  | Cytoplasmic |
| 5 | SGGB_1043 | 99 | 0 | transcriptional regulator | 0 |  | Cytoplasmic |
| 5 | SGGB_1044 | 99 | 0 | glucosyltransferase (glycosyl hydrolase family 70) | 1 |  | Extracellular |
| 5 | SGGB_1045 | 100 | 32 | transcriptional regulator | 0 |  | Cytoplasmic |
| 5 | SGGB_1046 | 95 | 0 | glucosyltransferase (glycosyl hydrolase family 70) | 0 |  | Extracellular |
| 5 | SGGB_1047 | 96 | 0 | glucan-binding protein C, GbpC (LPXTG motif) | 1 | Signal peptide | Cellwall |
| 5 | SGGB_1048 | 99 | 0 | MATE family multidrug efflux pumps | 11 |  | CytoplasmicMembrane |
| 5 | SGGB_1049 | 0 | 0 | conserved hypothetical protein | 0 |  | Cytoplasmic |
| 5 | SGGB_1050 | 0 | 97 | dihydroxyacetone kinase family protein | 0 |  | Cytoplasmic |
| 5 | SGGB_1051 | 0 | 100 | dihydroxyacetone kinase regulator, TetR family | 0 |  | Cytoplasmic |
| 5 | SGGB_1052 | 0 | 97 | dihydroxyacetone kinase, N-terminal domain | 0 |  | Cytoplasmic |
| 5 | SGGB_1053 | 0 | 93 | dihydroxyacetone kinase, C-terminal domain | 0 |  | Cytoplasmic |
| 5 | SGGB_1054 | 0 | 86 | dihydroxyacetone kinase, phosphotransfer subunit | 0 |  | Cytoplasmic |
| 5 | SGGB_1055 | 37 | 98 | glycerol uptake facilitator protein | 6 |  | CytoplasmicMembrane |
| 5 | SGGB_1056 | 0 | 0 | zeta toxin family protein | 0 |  | Unknown |
| 5 | SGGB_1057 | 81 | 0 | conserved hypothetical protein | 0 |  | Unknown |
| 5 | SGGB_1058 | 98 | 98 | conserved hypothetical protein | 0 |  | Cytoplasmic |
| 5 | SGGB_1059 | 0 | 0 | conserved hypothetical protein | 2 |  | Cytoplasmic |
| 5 | SGGB_1060 | 97 | 97 | GntR family transcriptional regulator | 0 |  | Cytoplasmic |
| 5 | SGGB_1061 | 0 | 0 | hypothetical protein | 0 |  | Cytoplasmic |
| 5 | SGGB_1063 | 99 | 43 | NAD-dependent oxidoreductase | 0 |  | Cytoplasmic |
| 5 | SGGB_1064 | 97 | 0 | hypothetical protein | 0 |  | Unknown |
| 5 | SGGB_1065 | 99 | 0 | oxidoreductase | 0 |  | Unknown |
| 5 | SGGB_1066 | 99 | 0 | YoeB family toxin | 0 |  | Unknown |
| 5 | SGGB_1067 | 100 | 0 | YefM family antitoxin | 0 |  | Unknown |
| 5 | SGGB_1068 | 100 | 98 | signal recognition particle, DNA-binding protein YlxM | 0 |  | Cytoplasmic |
| 5 | SGGB_1069 | 99 | 99 | signal recognition particle, subunit SRP54/Ffh | 0 |  | Cytoplasmic |
| 5 | SGGB_1070 | 99 | 0 | AraC family transcriptional regulator | 0 |  | Cytoplasmic |
| 5 | SGGB_1071 | 90 | 0 | ATP-binding cassette, subfamily B, bacterial | 5 |  | CytoplasmicMembrane |
| 6 | SGGB_1562 | 100 | 0 | addiction module toxin, RelE/StbE family | 0 |  | Cytoplasmic |
| 6 | SGGB_1563 | 100 | 0 | addiction module antitoxin, RelB/DinJ family | 0 |  | Unknown |
| 6 | SGGB_1564 | 100 | 0 | TetR family transcriptional regulator | 0 |  | Unknown |
| 6 | SGGB_1565 | 100 | 0 | myosin-crossreactive antigen | 0 |  | CytoplasmicMembrane |
| 6 | SGGB_1566 | 100 | 50 | Family 3 Sortases | 2 |  | Unknown |
| 6 | SGGB_1567 | 100 | 0 | fimbrial subunit B protein FszB (LPXTG motif) | 1 | Signal peptide | Cellwall |
| 6 | SGGB_1568 | 100 | 0 | Cna protein B-type domain-containing protein (LPXTG motif) | 1 | Signal peptide | Cellwall |
| 6 | SGGB_1569 | 100 | 0 | signal peptide containing protein | 1 | Signal peptide | Unknown |
| 6 | SGGB_1570 | 78 | 0 | signal peptide containing protein | 1 | Signal peptide | Unknown |
| 6 | SGGB_1571 | 97 | 0 | signal peptide containing protein | 1 | Signal peptide | Unknown |
| 6 | SGGB_1572 | 100 | 0 | signal peptide containing protein | 1 |  | Cytoplasmic |
| 6 | SGGB_1573 | 100 | 0 | transposase, Gram-positive bacteria | 0 |  | Unknown |
| 6 | SGGB_1574 | 100 | 0 | hypothetical protein | 0 |  | Cytoplasmic |
| 6 | SGGB_1575 | 100 | 0 | hypothetical protein | 0 |  | Cytoplasmic |
| 6 | SGGB_1576 | 100 | 0 | extracellular pectate lyase | 1 | Signal peptide | Extracellular |
| 6 | SGGB_1577 | 100 | 0 | extracellular pectate lyase | 1 | Signal peptide | Extracellular |
| 6 | SGGB_1578 | 100 | 100 | threonyl-tRNA synthetase | 0 |  | Cytoplasmic |
| 6 | SGGB_1579 | 100 | 100 | 1,2-diacylglycerol 3-glucosyltransferase | 0 |  | Cytoplasmic |
| 6 | SGGB_1580 | 100 | 97 | glycosyl transferases group 1 | 0 |  | Cytoplasmic |
| 6 | SGGB_1581 | 100 | 99 | catabolite control protein A | 0 |  | Cytoplasmic |
| 6 | SGGB_1582 | 100 | 99 | X-Pro dipeptidase | 0 |  | Cytoplasmic |
| 6 | SGGB_1583 | 100 | 0 | small multidrug resistance protein, SMR family | 3 |  | CytoplasmicMembrane |
| 6 | SGGB_1584 | 100 | 0 | NAD+ diphosphatase | 0 |  | Cytoplasmic |
| 6 | SGGB_1585 | 100 | 0 | predicted lipoprotein | 0 | Lipoprotein signal peptide | Unknown |
| 6 | SGGB_1586 | 100 | 0 | Tn916 ORF2 integrase | 0 |  | Unknown |
| 6 | SGGB_1587 | 100 | 0 | Tn916 ORF1 excisionase | 0 |  | Cytoplasmic |
| 6 | SGGB_1588 | 100 | 0 | Tn916 ORF7 DNA-binding protein protein | 0 |  | Cytoplasmic |
| 6 | SGGB_1589 | 100 | 0 | Tn916 ORF9 transcriptional regulator, putative | 0 |  | Cytoplasmic |
| 6 | SGGB_1590 | 95 | 0 | Tn916 ORF11 tetracycline resistance protein | 0 |  | Cytoplasmic |
| 6 | SGGB_1591 | 99 | 100 | Tn916 ORF13 protein | 1 |  | Unknown |
| 6 | SGGB_1592 | 100 | 100 | Tn916 ORF14 extracellular hydrolase/peptidase | 1 | Signal peptide | Extracellular |
| 6 | SGGB_1593 | 93 | 100 | Tn916 ORF15 signal peptide containing protein | 8 | Signal peptide | CytoplasmicMembrane |
| 6 | SGGB_1594 | 100 | 100 | Tn916 ORF16 ATP/GTP-binding protein | 0 |  | Cytoplasmic |
| 6 | SGGB_1595 | 100 | 100 | Tn916 ORF17 signal peptide containing protein | 3 | Signal peptide | CytoplasmicMembrane |
| 6 | SGGB_1596 | 99 | 100 | Tn916 ORF18 antirestriction (ArdA) protein | 0 |  | Cytoplasmic |
| 6 | SGGB_1597 | 100 | 100 | Tn916 ORF19 protein | 2 |  | CytoplasmicMembrane |
| 6 | SGGB_1598 | 100 | 100 | Tn916 ORF20 phage replication initiation factor PEP | 0 |  | Cytoplasmic |
| 6 | SGGB_1599 | 100 | 73 | Tn916 ORF21 FtsK/SpoIIIE family protein | 2 |  | Unknown |
| 6 | SGGB_1600 | 100 | 57 | Tn916 ORF22 protein | 0 |  | Cytoplasmic |
| 6 | SGGB_1601 | 100 | 53 | Tn916 ORF23 protein | 0 |  | Cytoplasmic |
| 6 | SGGB_1602 | 100 | 0 | cell wall surface protein (LPXTG motif) | 2 |  | Extracellular |
| 6 | SGGB_1603 | 100 | 37 | PTS system, beta-glucosides-specific IIABC component | 10 |  | CytoplasmicMembrane |
| 6 | SGGB_1604 | 100 | 0 | neutral/alkaline non-lysosomal ceramidase | 0 |  | Cytoplasmic |
| 6 | SGGB_1605 | 100 | 0 | neutral/alkaline non-lysosomal ceramidase | 0 |  | Cytoplasmic |
| 6 | SGGB_1606 | 100 | 0 | amidohydrolases | 0 |  | Unknown |
| 6 | SGGB_1607 | 100 | 31 | LysR family transcriptional regulator | 0 |  | Cytoplasmic |
| 6 | SGGB_1608 | 100 | 0 | MATE family multidrug efflux pumps | 12 |  | CytoplasmicMembrane |
| 6 | SGGB_1609 | 100 | 0 | short-chain dehydrogenase/reductase SDR | 0 |  | Cytoplasmic |
| 6 | SGGB_1610 | 100 | 0 | beta-phosphoglucomutase | 0 |  | Unknown |
| 6 | SGGB_1611 | 100 | 35 | beta-N-acetylhexosaminidase | 0 |  | Cytoplasmic |
| 6 | SGGB_1612 | 100 | 34 | beta-N-acetylhexosaminidase | 0 |  | Cytoplasmic |
| 6 | SGGB_1613 | 100 | 0 | hypothetical protein | 0 |  | Cytoplasmic |
| 6 | SGGB_1614 | 100 | 0 | hypothetical protein | 0 |  | Cytoplasmic |
| 6 | SGGB_1615 | 100 | 0 | aldo/keto reductase family protein | 0 |  | Cytoplasmic |
| 6 | SGGB_1616 | 100 | 0 | predicted lipoprotein | 1 | Lipoprotein signal peptide | Unknown |
| 6 | SGGB_1617 | 100 | 0 | sugar (glycoside-pentoside-hexuronide) transporter | 11 |  | CytoplasmicMembrane |
| 6 | SGGB_1618 | 100 | 0 | amidohydrolase | 0 |  | Cytoplasmic |
| 7 | SGGB_1660 | 100 | 0 | Tn5276 integrase | 0 |  | Cytoplasmic |
| 7 | SGGB_1661 | 100 | 0 | hypothetical protein | 0 |  | Unknown |
| 7 | SGGB_1662 | 0 | 0 | hypothetical protein | 1 |  | Cytoplasmic |
| 7 | SGGB_1663 | 100 | 0 | conserved hypothetical protein | 0 |  | Cytoplasmic |
| 7 | SGGB_1664 | 92 | 0 | cell wall surface protein (LPXTG motif) | 1 | Signal peptide | Cellwall |
| 7 | SGGB_1665 | 95 | 0 | predicted lipoprotein | 1 | Lipoprotein signal peptide | Unknown |
| 7 | SGGB_1666 | 94 | 64 | sortase A | 1 | Signal peptide | CytoplasmicMembrane |
| 7 | SGGB_1667 | 92 | 0 | signal peptide containing protein | 1 | Signal peptide | CytoplasmicMembrane |
| 7 | SGGB_1668 | 86 | 0 | ICESt1 ORFC | 7 |  | CytoplasmicMembrane |
| 7 | SGGB_1669 | 99 | 0 | ICESt1 ORFD ATP/GTP-binding protein | 0 |  | Cytoplasmic |
| 7 | SGGB_1670 | 85 | 0 | ICESt1 ORFE | 1 |  | Cytoplasmic |
| 7 | SGGB_1671 | 73 | 0 | ICESt1 ORFF | 2 |  | CytoplasmicMembrane |
| 7 | SGGB_1672 | 97 | 0 | ICESt1 ORFG | 1 |  | Unknown |
| 7 | SGGB_1673 | 99 | 0 | conserved hypothetical protein | 0 |  | Cytoplasmic |
| 7 | SGGB_1674 | 73 | 0 | hypothetical protein | 0 |  | Cytoplasmic |
| 7 | SGGB_1675 | 98 | 0 | Tn916 ORF18 antirestriction (ArdA) protein | 0 |  | Cytoplasmic |
| 7 | SGGB_1676 | 0 | 0 | hypothetical protein | 0 |  | Unknown |
| 7 | SGGB_1677 | 0 | 0 | hypothetical protein | 0 |  | Unknown |
| 7 | SGGB_1678 | 100 | 0 | IS861 ORF2 | 0 |  | Unknown |
| 7 | SGGB_1679 | 100 | 0 | IS861 ORF1 | 0 |  | Cytoplasmic |
| 7 | SGGB_1680 | 96 | 0 | ICESt1 APR2 Cro/CI family transcriptional regulator | 0 |  | Cytoplasmic |
| 7 | SGGB_1681 | 94 | 0 | predicted membrane protein | 1 |  | Unknown |
| 7 | SGGB_1682 | 56 | 0 | conserved hypothetical protein | 0 |  | Cytoplasmic |
| 7 | SGGB_1683 | 73 | 0 | ICESt1 ORFJ phage replication initiation factor PEP | 0 |  | Cytoplasmic |
| 7 | SGGB_1684 | 100 | 0 | ICESt1 ORFK FtsK/SpoIIIE family protein | 2 | Signal peptide | Unknown |
| 7 | SGGB_1685 | 99 | 0 | ICESt1 ORFL | 0 |  | Unknown |
| 7 | SGGB_1686 | 100 | 0 | ICESt1 ORFM | 0 |  | Cytoplasmic |
| 7 | SGGB_1687 | 100 | 0 | cell wall surface protein (LPXTG motif) | 1 |  | Cellwall |
| 7 | SGGB_1688 | 99 | 0 | conserved hypothetical protein | 0 |  | Unknown |
| 7 | SGGB_1689 | 99 | 0 | thioredoxin, signal peptide containing | 1 |  | Unknown |
| 7 | SGGB_1690 | 99 | 0 | conserved hypothetical protein | 0 |  | Cytoplasmic |
| 7 | SGGB_1691 | 100 | 0 | hypothetical protein | 0 |  | Unknown |
| 7 | SGGB_1692 | 100 | 0 | conserved hypothetical protein | 0 |  | Cytoplasmic |
| 7 | SGGB_1693 | 100 | 0 | nucleotidyltransferase domain-containing protein | 0 |  | Cytoplasmic |
| 7 | SGGB_1694 | 0 | 0 | hypothetical protein | 0 |  | Unknown |
| 7 | SGGB_1695 | 100 | 0 | predicted membrane protein | 1 |  | Cytoplasmic |
| 7 | SGGB_1696 | 0 | 0 | ICESt1 APR2 Cro/CI family transcriptional regulator | 0 |  | Cytoplasmic |
| 7 | SGGB_1697 | 100 | 0 | IS1272 ORF1 and ORF2 | 1 |  | Unknown |
| 7 | SGGB_1698 | 93 | 0 | predicted lipoprotein | 1 | Lipoprotein signal peptide | Unknown |
| 7 | SGGB_1699 | 95 | 0 | addiction module antitoxin, RelB/DinJ family | 0 |  | Unknown |
| 7 | SGGB_1700 | 100 | 0 | pyridine nucleotide-disulphide oxidoreductase | 0 |  | Cytoplasmic |
| 7 | SGGB_1701 | 100 | 100 | conserved hypothetical protein | 0 |  | Unknown |
| 7 | SGGB_1702 | 100 | 31 | major facilitator superfamily protein | 12 |  | CytoplasmicMembrane |
| 7 | SGGB_1703 | 100 | 0 | signal peptide containing protein | 1 | Signal peptide | Unknown |
| 8 | SGGB_2111 | 100 | 0 | conserved hypothetical protein | 0 |  | Cytoplasmic |
| 8 | SGGB_2112 | 100 | 0 | iron-containing alcohol dehydrogenase | 0 |  | Cytoplasmic |
| 8 | SGGB_2113 | 100 | 36 | D-methionine transport system substrate-binding protein | 1 | Lipoprotein signal peptide | Unknown |
| 8 | SGGB_2114 | 100 | 38 | D-methionine transport system permease protein | 6 |  | CytoplasmicMembrane |
| 8 | SGGB_2115 | 100 | 40 | D-methionine transport system ATP-binding protein | 0 |  | CytoplasmicMembrane |
| 8 | SGGB_2116 | 100 | 0 | Rrf2 family transcriptional regulators | 0 |  | Cytoplasmic |
| 8 | SGGB_2117 | 100 | 100 | adenylosuccinate synthase | 0 |  | Cytoplasmic |
| 8 | SGGB_2118 | 100 | 98 | bifunctional glutamate--cysteine ligase/glutathione synthase | 0 |  | Cytoplasmic |
| 8 | SGGB_2119 | 100 | 0 | putative phage protein | 0 |  | Cytoplasmic |
| 8 | SGGB_2120 | 100 | 0 | putative phage protein | 0 |  | Cytoplasmic |
| 8 | SGGB_2121 | 100 | 0 | putative phage protein | 0 |  | Cytoplasmic |
| 8 | SGGB_2122 | 100 | 0 | putative phage protein | 0 |  | Unknown |
| 8 | SGGB_2123 | 100 | 0 | putative phage protein | 0 |  | Cytoplasmic |
| 8 | SGGB_2124 | 100 | 0 | putative phage protein | 0 |  | Unknown |
| 8 | SGGB_2125 | 99 | 0 | putative phage protein | 0 |  | Cytoplasmic |
| 8 | SGGB_2126 | 100 | 0 | predicted phage membrane protein | 1 |  | Cytoplasmic |
| 8 | SGGB_2127 | 100 | 0 | putative phage protein | 0 |  | Cytoplasmic |
| 8 | SGGB_2128 | 100 | 0 | phage transcriptional regulator | 0 |  | Unknown |
| 8 | SGGB_2129 | 100 | 0 | phage transcriptional regulator | 0 |  | Unknown |
| 8 | SGGB_2130 | 99 | 0 | putative phage protein | 0 |  | Unknown |
| 8 | SGGB_2131 | 100 | 0 | predicted membrane protein | 2 |  | Unknown |
| 8 | SGGB_2132 | 100 | 73 | phage integrase family recombinase | 0 |  | Unknown |
| 8 | SGGB_2133 | 100 | 100 | predicted membrane protein | 2 |  | Cytoplasmic |
| 8 | SGGB_2134 | 100 | 98 | toxic anion resistance protein | 0 |  | Cytoplasmic |
| 8 | SGGB_2135 | 100 | 0 | LytTr family transcriptional regulator | 0 |  | Cytoplasmic |
| 8 | SGGB_2136 | 100 | 0 | antibiotic transport system permease protein | 6 |  | CytoplasmicMembrane |
| 8 | SGGB_2137 | 100 | 0 | ABC-2 type transport system ATP-binding protein | 0 |  | CytoplasmicMembrane |
| 8 | SGGB_2138 | 100 | 0 | hypothetical protein | 0 |  | Cytoplasmic |
| 8 | SGGB_2139 | 100 | 0 | nitroreductase family protein | 0 |  | Unknown |
| 8 | SGGB_2140 | 100 | 0 | YcaO-like family protein | 0 |  | Cytoplasmic |
| 8 | SGGB_2141 | 100 | 0 | hypothetical protein | 0 |  | Cytoplasmic |
| 8 | SGGB_2142 | 100 | 99 | molecular chaperone Hsp33 | 0 |  | Unknown |
| 8 | SGGB_2143 | 100 | 99 | tRNA-dihydrouridine synthase | 0 |  | Cytoplasmic |
| 8 | SGGB_2144 | 100 | 99 | ATP-dependent Clp protease, ATP-binding subunit ClpC | 0 |  | Cytoplasmic |
| 8 | SGGB_2145 | 100 | 99 | transcriptional regulator CtsR | 0 |  | Cytoplasmic |
| 8 | SGGB_2146 | 0 | 0 | hypothetical protein | 0 |  | Unknown |
| 8 | SGGB_2147 | 100 | 94 | putative small multi-drug export proteins | 4 |  | CytoplasmicMembrane |
| 8 | SGGB_2148 | 100 | 0 | transcriptional regulator | 0 |  | Cytoplasmic |
| 8 | SGGB_2149 | 100 | 0 | macrolide-efflux protein | 11 |  | CytoplasmicMembrane |
| 8 | SGGB_2150 | 100 | 99 | elongation factor EF-Ts | 0 |  | Cytoplasmic |
| 8 | SGGB_2151 | 100 | 100 | 30S ribosomal protein S2 | 0 |  | Cytoplasmic |
| 8 | SGGB_2152 | 48 | 45 | conserved hypothetical protein | 0 |  | Cytoplasmic |
| 8 | SGGB_2153 | 79 | 78 | sortase A | 1 | Signal peptide | Unknown |
| 8 | SGGB_2154 | 0 | 0 | conserved hypothetical protein | 3 |  | CytoplasmicMembrane |
| 8 | SGGB_2155 | 0 | 0 | Replication initiator protein A | 0 |  | Cytoplasmic |
| 8 | SGGB_2156 | 0 | 0 | conserved hypothetical protein | 0 |  | Cytoplasmic |
| 8 | SGGB_2157 | 0 | 37 | conserved hypothetical protein | 0 |  | Cytoplasmic |
| 8 | SGGB_2158 | 0 | 0 | signal peptide containing protein | 1 | Signal peptide | Unknown |
| 8 | SGGB_2159 | 0 | 0 | putative glucosaminidase, signal peptide containing | 1 | Signal peptide | Unknown |
| 8 | SGGB_2160 | 0 | 0 | conserved hypothetical protein | 1 |  | Unknown |
| 8 | SGGB_2161 | 0 | 0 | conserved hypothetical protein | 0 |  | Unknown |
| 8 | SGGB_2162 | 0 | 0 | conserved hypothetical protein | 0 |  | Cytoplasmic |
| 8 | SGGB_2163 | 0 | 0 | conserved hypothetical protein | 2 |  | CytoplasmicMembrane |
| 8 | SGGB_2164 | 0 | 0 | conserved hypothetical protein | 0 |  | Cytoplasmic |
| 8 | SGGB_2165 | 0 | 0 | conserved hypothetical protein | 6 |  | CytoplasmicMembrane |
| 8 | SGGB_2166 | 0 | 0 | predicted lipoprotein | 0 | Lipoprotein signal peptide | Unknown |
| 8 | SGGB_2167 | 0 | 0 | Type IV secretion-system coupling protein DNA-binding domain | 4 |  | CytoplasmicMembrane |
| 8 | SGGB_2168 | 0 | 0 | conserved hypothetical protein | 0 |  | Unknown |
| 8 | SGGB_2169 | 0 | 0 | cell surface-associated protein autolysin AtlA | 0 |  | Unknown |
| 8 | SGGB_2170 | 0 | 0 | cell wall surface protein (LPXTG motif) | 1 |  | Cellwall |
| 8 | SGGB_2171 | 83 | 0 | cell wall surface protein (LPXTG motif) | 0 | Signal peptide | Cellwall |
| 8 | SGGB_2172 | 0 | 0 | conserved hypothetical protein | 1 |  | Unknown |
| 8 | SGGB_2173 | 0 | 0 | TrbC/VIRB2 family membrane protein, signal peptide containing | 3 | Signal peptide | CytoplasmicMembrane |
| 8 | SGGB_2174 | 43 | 42 | ATP-dependent chaperone protein | 0 |  | Cytoplasmic |
| 8 | SGGB_2175 | 46 | 46 | DNA topoisomerase III | 0 |  | Cytoplasmic |
| 8 | SGGB_2176 | 0 | 0 | putative extracellular protein | 0 |  | Extracellular |
| 8 | SGGB_2177 | 0 | 0 | conserved hypothetical protein | 0 |  | Cytoplasmic |
| 8 | SGGB_2178 | 0 | 0 | conserved hypothetical protein | 0 |  | Unknown |
| 8 | SGGB_2179 | 0 | 0 | conserved hypothetical protein | 0 |  | Cytoplasmic |
| 8 | SGGB_2180 | 0 | 0 | conserved hypothetical protein | 0 |  | Unknown |
| 8 | SGGB_2181 | 0 | 0 | DNA-entry nuclease, signal peptide containing | 1 | Signal peptide | Unknown |
| 8 | SGGB_2182 | 0 | 0 | conserved hypothetical protein | 0 |  | Cytoplasmic |
| 8 | SGGB_2183 | 0 | 0 | conserved hypothetical protein | 0 |  | Cytoplasmic |
| 8 | SGGB_2184 | 0 | 0 | conserved hypothetical protein | 0 |  | Cytoplasmic |
| 8 | SGGB_2185 | 0 | 0 | conserved hypothetical protein | 2 |  | Cytoplasmic |
| 8 | SGGB_2186 | 0 | 0 | conserved hypothetical protein | 1 |  | Unknown |
| 8 | SGGB_2187 | 0 | 0 | hypothetical protein | 0 |  | Unknown |
| 8 | SGGB_2188 | 0 | 0 | hypothetical protein | 2 |  | Unknown |
| 8 | SGGB_2189 | 0 | 0 | hypothetical protein | 2 |  | Unknown |
| 8 | SGGB_2190 | 0 | 0 | hypothetical protein | 2 |  | Unknown |
| 8 | SGGB_2191 | 42 | 0 | thioredoxin/glutaredoxin, signal peptide containing | 1 |  | Unknown |
| 8 | SGGB_2192 | 0 | 0 | conserved hypothetical protein | 0 |  | Cytoplasmic |
| 8 | SGGB_2193 | 0 | 0 | putative extracellular protein | 2 | Signal peptide | Extracellular |
| 8 | SGGB_2194 | 0 | 0 | conserved hypothetical protein | 0 |  | Unknown |
| 8 | SGGB_2195 | 0 | 0 | hypothetical protein | 1 | Signal peptide | Unknown |
| 8 | SGGB_2196 | 0 | 0 | RepE/RepS primase family protein | 0 |  | Unknown |
| 8 | SGGB_2197 | 0 | 0 | hypothetical protein | 0 |  | Unknown |
| 8 | SGGB_2198 | 0 | 0 | hypothetical protein | 0 |  | Cytoplasmic |
| 8 | SGGB_2199 | 0 | 0 | conserved hypothetical protein | 0 |  | Cytoplasmic |
| 8 | SGGB_2200 | 60 | 60 | DNA (cytosine-5-)-methyltransferase | 0 |  | Cytoplasmic |
| 8 | SGGB_2201 | 0 | 0 | hypothetical protein | 0 |  | Cytoplasmic |

1. Denotes the percentage identity between aligned ATCC 43143 and UCN34 proteins. Alignments with percentage identity below 30 were considered highly dissimilar and were regarded as proteins with no BLAST hits and given a value of zero.
2. Denotes the percentage identity between aligned ATCC 43143 and ATCC 43144 proteins. Alignments with percentage identity below 30 were considered highly dissimilar and were regarded as proteins with no BLAST hits and given a value of zero.
