# Supplementary material for: Sequencing and Comparative Genome Analysis of Two Pathogenic Streptococcus gallolyticus Subspecies: Genome Plasticity, Adaptation and Virulence
Source: PLoS One. 2011 May 25;6(5):e20519. doi: 10.1371/journal.pone.0020519 (PMC3102119; doi:10.1371/journal.pone.0020519)
Supplement: Table S6 — List of regions of genomic plasticity (RGPs) in the ATCC 43144 genome. A table listing the identified RGPs in S. pasteurianus ATCC 43144. (DOC) [file pone.0020519.s009.doc]

**Table S6. List of regions of genomic plasticity (RGPs) in the ATCC 43144 genome.** A table listing the identified RGPs in *S. pasteurianus* ATCC 43144.

| **RGP No.** | **ATCC 43144 Locus ID** | **UCN34**  **% Homology ^a)^** | **ATCC 43143**  **% Homology ^b)^** | **Descriptions** | **No. of TMD** | **Signal Peptide Prediction** | **Localization Prediction** |
| --- | --- | --- | --- | --- | --- | --- | --- |
| 1 | SGPB_0813 | 0 | 0 | polysaccharide biosynthesis protein | 3 |  | CytoplasmicMembrane |
| 1 | SGPB_0814 | 0 | 0 | aminotransferase family protein | 0 |  | Cytoplasmic |
| 1 | SGPB_0815 | 0 | 0 | bacterial sugar transferase | 1 |  | CytoplasmicMembrane |
| 1 | SGPB_0816 | 0 | 0 | glycosyl transferase family 2 | 0 |  | Unknown |
| 1 | SGPB_0817 | 0 | 0 | glycosyltransferase | 0 |  | Cytoplasmic |
| 1 | SGPB_0818 | 0 | 0 | glycosyl transferase family 1 | 1 |  | Unknown |
| 1 | SGPB_0819 | 0 | 0 | hypothetical protein | 0 |  | Cytoplasmic |
| 1 | SGPB_0820 | 0 | 0 | glycosyl transferase family 2 | 0 |  | Cytoplasmic |
| 1 | SGPB_0821 | 0 | 0 | polysaccharide polymerase | 10 |  | CytoplasmicMembrane |
| 1 | SGPB_0822 | 0 | 0 | polysaccharide flippase transporter | 14 |  | CytoplasmicMembrane |
| 1 | SGPB_0823 | 0 | 0 | UDPglucose 6-dehydrogenase | 0 |  | Unknown |
| 1 | SGPB_0824 | 94 | 0 | transcription antiterminator | 0 |  | CytoplasmicMembrane |
| 1 | SGPB_0825 | 75 | 0 | PTS system, fructose-specific IIA component | 0 |  | Cytoplasmic |
| 1 | SGPB_0826 | 96 | 0 | PTS system, fructose-specific IIB component | 0 |  | Cytoplasmic |
| 1 | SGPB_0827 | 99 | 0 | PTS system, fructose-specific IIC component | 9 |  | CytoplasmicMembrane |
| 1 | SGPB_0828 | 99 | 0 | alpha-mannosidase | 0 |  | Cytoplasmic |
| 1 | SGPB_0829 | 96 | 96 | conserved hypothetical protein | 0 |  | Cytoplasmic |
| 2 | SGPB_0937 | 0 | 92 | predicted membrane protein | 1 |  | Extracellular |
| 2 | SGPB_0940 | 99 | 99 | glucose inhibited division protein Gid | 0 |  | Cytoplasmic |
| 2 | SGPB_0941 | 95 | 94 | predicted membrane protein | 5 |  | CytoplasmicMembrane |
| 2 | SGPB_0942 | 0 | 0 | secretion and acid tolerance protein SatE | 3 |  | Unknown |
| 2 | SGPB_0943 | 97 | 97 | secretion and acid tolerance protein SatD | 0 |  | Cytoplasmic |
| 2 | SGPB_0946 | 98 | 98 | late competence protein; DNA processing/uptake | 0 |  | Cytoplasmic |
| 2 | SGPB_0947 | 98 | 98 | maltose O-acetyltransferase | 0 |  | Cytoplasmic |
| 2 | SGPB_0948 | 98 | 98 | ribonuclease HII | 0 |  | Cytoplasmic |
| 2 | SGPB_0949 | 98 | 98 | ribosomal biogenesis GTPase | 0 |  | Cytoplasmic |
| 2 | SGPB_0951 | 0 | 0 | lantibiotic ABC transporter permease protein | 7 |  | CytoplasmicMembrane |
| 2 | SGPB_0952 | 51 | 51 | lantibiotic ABC transporter ATP-binding protein | 0 |  | CytoplasmicMembrane |
| 2 | SGPB_0953 | 34 | 34 | beta-N-acetylhexosaminidase | 0 |  | Cytoplasmic |
| 2 | SGPB_0954 | 34 | 34 | beta-N-acetylhexosaminidase | 0 |  | Cytoplasmic |
| 2 | SGPB_0955 | 0 | 0 | phosphoglycolate phosphatase | 0 |  | Unknown |
| 2 | SGPB_0956 | 0 | 0 | conserved hypothetical protein | 0 |  | Cytoplasmic |
| 2 | SGPB_0957 | 0 | 0 | mannonate dehydrogenase | 0 |  | Cytoplasmic |
| 2 | SGPB_0958 | 0 | 0 | mannonate dehydratase | 0 |  | Cytoplasmic |
| 2 | SGPB_0959 | 0 | 0 | glucuronate isomerase | 0 |  | Cytoplasmic |
| 2 | SGPB_0960 | 39 | 38 | bifunctional keto-hydroxyglutarate-aldolase/keto-deoxy-phosphogluconate aldolase | 0 |  | Cytoplasmic |
| 2 | SGPB_0961 | 0 | 0 | GntR family regulatory protein | 0 |  | Cytoplasmic |
| 2 | SGPB_0962 | 0 | 0 | beta-glucuronidase | 0 |  | Cytoplasmic |
| 2 | SGPB_0963 | 0 | 0 | 2-dehydro-3-deoxygluconokinase | 0 |  | Cytoplasmic |
| 2 | SGPB_0964 | 0 | 0 | glucuronide transporter | 9 |  | CytoplasmicMembrane |
| 2 | SGPB_0965 | 99 | 99 | ABC transporter, ATP-binding protein | 0 |  | CytoplasmicMembrane |
| 2 | SGPB_0966 | 97 | 97 | predicted membrane protein | 5 |  | CytoplasmicMembrane |
| 2 | SGPB_0967 | 97 | 97 | pyridoxine kinase | 0 |  | MultipleSites |
| 2 | SGPB_0968 | 98 | 98 | GntR family regulatory protein | 0 |  | Cytoplasmic |
| 2 | SGPB_0969 | 100 | 100 | dihydrodipicolinate synthase | 0 |  | Cytoplasmic |
| 2 | SGPB_0970 | 100 | 100 | aspartate-semialdehyde dehydrogenase | 0 |  | Cytoplasmic |
| 2 | SGPB_0971 | 98 | 98 | ATP-binding cassette, subfamily B, bacterial | 3 |  | CytoplasmicMembrane |
| 2 | SGPB_0972 | 0 | 0 | predicted membrane protein | 6 |  | CytoplasmicMembrane |
| 2 | SGPB_0973 | 32 | 32 | ABC transport system ATP-binding protein | 0 |  | CytoplasmicMembrane |
| 2 | SGPB_0974 | 0 | 0 | predicted membrane protein | 6 |  | CytoplasmicMembrane |
| 2 | SGPB_0975 | 0 | 0 | ECF subfamily RNA polymerase sigma-24 factor | 0 |  | Cytoplasmic |
| 2 | SGPB_0976 | 99 | 99 | formate--tetrahydrofolate ligase | 0 |  | Cytoplasmic |
| 2 | SGPB_0977 | 0 | 0 | signal peptide containing protein | 0 | Signal peptide | Unknown |
| 3 | SGPB_1095 | 0 | 97 | putative extracellular protein | 2 |  | Extracellular |
| 3 | SGPB_1097 | 0 | 0 | Tn5276 integrase | 0 |  | Cytoplasmic |
| 3 | SGPB_1098 | 0 | 0 | conserved hypothetical protein | 0 |  | Cytoplasmic |
| 3 | SGPB_1099 | 0 | 0 | conserved hypothetical protein | 0 |  | Unknown |
| 3 | SGPB_1100 | 0 | 0 | nisin immunity protein | 1 | Lipoprotein signal peptide | Unknown |
| 3 | SGPB_1101 | 0 | 0 | lanthionine synthetase C-like protein | 0 |  | Cytoplasmic |
| 3 | SGPB_1104 | 0 | 0 | lantibiotic dehydratase | 0 |  | Cytoplasmic |
| 3 | SGPB_1105 | 0 | 0 | nisin U lantibiotic | 0 |  | Unknown |
| 3 | SGPB_1106 | 42 | 42 | Immunity membrane protein | 6 |  | CytoplasmicMembrane |
| 3 | SGPB_1107 | 52 | 52 | lantibiotic protection ABC transporter permease protein | 6 | Signal peptide | CytoplasmicMembrane |
| 3 | SGPB_1108 | 68 | 68 | lantibiotic protection ABC transporter ATP-binding protein | 0 |  | CytoplasmicMembrane |
| 3 | SGPB_1109 | 44 | 44 | two-component system, sensor histidine kinase, lantibiotic associated | 2 |  | MultipleSites |
| 3 | SGPB_1110 | 65 | 65 | two-component system, response regulator, lantibiotic associated | 0 |  | Cytoplasmic |
| 3 | SGPB_1111 | 0 | 0 | lantibiotic leader peptide processing serine protease | 1 | Signal peptide | Extracellular |
| 3 | SGPB_1112 | 0 | 0 | Tn5252 ORF4 relaxase | 0 |  | Cytoplasmic |
| 3 | SGPB_1113 | 0 | 0 | Tn5252 ORF9 | 0 |  | Cytoplasmic |
| 3 | SGPB_1114 | 0 | 0 | Tn5252 ORF10 | 0 |  | Cytoplasmic |
| 3 | SGPB_1115 | 0 | 0 | ICESt1 ORFD ATP/GTP-binding protein (Tn916 ORF16 related) | 0 |  | Cytoplasmic |
| 3 | SGPB_1116 | 0 | 0 | ICESt1 ORFV2 | 0 |  | Cytoplasmic |
| 3 | SGPB_1117 | 0 | 0 | PezT Zeta toxin | 0 |  | Cytoplasmic |
| 3 | SGPB_1118 | 0 | 0 | PezA epsilon antitoxin | 0 |  | Cytoplasmic |
| 3 | SGPB_1119 | 0 | 0 | glutamate-cysteine ligas | 0 |  | Cytoplasmic |
| 3 | SGPB_1120 | 0 | 0 | conserved hypothetical protein | 0 |  | Unknown |
| 3 | SGPB_1121 | 0 | 0 | DNA primase | 0 |  | Cytoplasmic |
| 3 | SGPB_1122 | 0 | 0 | predicted membrane protein | 1 | Signal peptide | Unknown |
| 3 | SGPB_1123 | 0 | 0 | TetR family transcriptional regulator | 0 |  | Cytoplasmic |
| 3 | SGPB_1124 | 0 | 0 | conserved hypothetical protein | 0 |  | Cytoplasmic |
| 3 | SGPB_1125 | 0 | 0 | conserved hypothetical protein | 0 |  | Cytoplasmic |
| 3 | SGPB_1127 | 0 | 0 | reverse transcriptase (RNA-dependent DNA polymerase) | 0 |  | Unknown |
| 3 | SGPB_1128 | 0 | 0 | SNF2/helicase family protein | 0 |  | Cytoplasmic |
| 3 | SGPB_1129 | 0 | 0 | conserved hypothetical protein | 0 |  | Cytoplasmic |
| 3 | SGPB_1130 | 0 | 0 | calcium-binding protein | 0 |  | Unknown |
| 3 | SGPB_1131 | 0 | 0 | glucan-binding protein C family protein (LPXTG motif) | 1 |  | Cellwall |
| 3 | SGPB_1132 | 0 | 0 | abortive infection system Abi protein | 0 |  | Cytoplasmic |
| 3 | SGPB_1133 | 0 | 0 | IS116/IS110/IS902 family transposase | 0 |  | Cytoplasmic |
| 3 | SGPB_1134 | 0 | 0 | abortive infection system Abi protein | 0 |  | Unknown |
| 3 | SGPB_1135 | 35 | 0 | Tn5252 Orf28 | 1 |  | Unknown |
| 3 | SGPB_1136 | 37 | 0 | Tn5252 Orf26 | 0 |  | Unknown |
| 3 | SGPB_1137 | 0 | 0 | Tn5252 Orf25 | 2 |  | Unknown |
| 3 | SGPB_1138 | 0 | 0 | Tn5252 Orf23 | 6 |  | CytoplasmicMembrane |
| 3 | SGPB_1139 | 0 | 0 | predicted membrane protein | 2 |  | Unknown |
| 3 | SGPB_1140 | 37 | 0 | Tn5252 Orf21 type IV secretion system protein VirD4 | 3 |  | CytoplasmicMembrane |
| 3 | SGPB_1141 | 0 | 0 | conserved hypothetical protein | 0 |  | Cytoplasmic |
| 3 | SGPB_1142 | 0 | 0 | CAAX amino terminal protease family protein | 6 |  | CytoplasmicMembrane |
| 3 | SGPB_1143 | 0 | 0 | conserved hypothetical protein | 0 |  | Unknown |
| 3 | SGPB_1144 | 0 | 0 | conserved hypothetical protein | 0 |  | Cytoplasmic |
| 3 | SGPB_1145 | 0 | 59 | DNA (cytosine-5-)-methyltransferase | 0 |  | Cytoplasmic |
| 3 | SGPB_1146 | 0 | 0 | replication initiator protein A | 0 |  | Unknown |
| 3 | SGPB_1147 | 0 | 0 | conserved hypothetical protein | 0 |  | Cytoplasmic |
| 3 | SGPB_1148 | 0 | 0 | conserved hypothetical protein | 0 |  | Unknown |
| 3 | SGPB_1149 | 0 | 0 | Tn5276 integrase | 0 |  | Cytoplasmic |
| 3 | SGPB_1150 | 0 | 0 | conserved hypothetical protein | 0 |  | Cytoplasmic |
| 3 | SGPB_1152 | 48 | 48 | predicted membrane protein | 1 | Signal peptide | Cytoplasmic |
| 3 | SGPB_1153 | 85 | 85 | FtsK/SpoIIIE family protein | 0 |  | Unknown |
| 3 | SGPB_1154 | 87 | 87 | conserved hypothetical protein | 0 |  | Unknown |
| 3 | SGPB_1156 | 0 | 0 | predicted lipoprotein | 0 | Lipoprotein signal peptide | Unknown |
| 3 | SGPB_1157 | 0 | 0 | putative transcriptional regulator | 0 |  | Cytoplasmic |
| 4 | SGPB_1229 | 46 | 45 | conserved hypothetical protein | 0 |  | Unknown |
| 4 | SGPB_1230 | 0 | 0 | filamentation induced by cAMP protein Fic | 0 |  | Cytoplasmic |
| 4 | SGPB_1231 | 0 | 0 | conserved hypothetical protein | 0 |  | Unknown |
| 4 | SGPB_1232 | 0 | 37 | chromosome/plasmid partitioning protein | 0 |  | Unknown |
| 4 | SGPB_1233 | 0 | 0 | hypothetical protein | 0 |  | Cytoplasmic |
| 4 | SGPB_1234 | 0 | 0 | resolvase | 0 |  | Cytoplasmic |
| 4 | SGPB_1235 | 0 | 0 | predicted membrane protein | 2 |  | Unknown |
| 4 | SGPB_1239 | 91 | 46 | DNA topoisomerase | 0 |  | Cytoplasmic |
| 4 | SGPB_1240 | 0 | 0 | hypothetical protein | 0 |  | Unknown |
| 4 | SGPB_1241 | 79 | 0 | Tn5252 Orf21 DNA-binding protein | 2 |  | Unknown |
| 4 | SGPB_1242 | 82 | 0 | conserved hypothetical protein | 0 |  | Cytoplasmic |
| 4 | SGPB_1243 | 57 | 0 | predicted membrane protein | 1 |  | Unknown |
| 4 | SGPB_1244 | 70 | 0 | conserved hypothetical protein | 0 |  | Unknown |
| 4 | SGPB_1245 | 38 | 38 | single-strand DNA-binding protein | 0 |  | Unknown |
| 4 | SGPB_1246 | 0 | 0 | predicted membrane protein | 1 |  | Unknown |
| 4 | SGPB_1249 | 0 | 0 | predicted membrane protein | 1 |  | Extracellular |
| 5 | SGPB_1520 | 42 | 42 | ROK family glucokinase | 0 |  | Cytoplasmic |
| 5 | SGPB_1521 | 57 | 57 | beta-glucosidase | 0 |  | Cytoplasmic |
| 5 | SGPB_1522 | 0 | 0 | predicted membrane protein | 6 |  | CytoplasmicMembrane |
| 5 | SGPB_1523 | 0 | 0 | endo-beta-N-acetylglucosaminidase | 0 | Lipoprotein signal peptide | Unknown |
| 5 | SGPB_1524 | 56 | 56 | glucan 1,6-alpha-glucosidase | 0 |  | Cytoplasmic |
| 5 | SGPB_1525 | 0 | 0 | multiple sugar transport system substrate-binding protein | 0 | Lipoprotein signal peptide | Unknown |
| 5 | SGPB_1526 | 30 | 30 | multiple sugar transport system permease protein | 6 | Signal peptide | CytoplasmicMembrane |
| 5 | SGPB_1527 | 0 | 0 | multiple sugar transport system permease protein | 6 | Signal peptide | CytoplasmicMembrane |
| 5 | SGPB_1528 | 0 | 0 | alpha-mannosidase | 0 |  | Cytoplasmic |
| 5 | SGPB_1530 | 0 | 0 | conserved hypothetical protein | 0 |  | Cytoplasmic |
| 5 | SGPB_1531 | 0 | 0 | GntR family transcriptional regulator | 0 |  | Cytoplasmic |
| 5 | SGPB_1532 | 0 | 0 | alpha-1,2-mannosidase | 0 |  | Unknown |
| 5 | SGPB_1533 | 0 | 0 | hypothetical protein | 0 |  | Unknown |

1. Denotes the percentage identity between aligned ATCC 43144 and UCN34 proteins. Alignments with percentage identity below 30 were considered highly dissimilar and were regarded as proteins with no BLAST hits and given a value of zero.
2. Denotes the percentage identity between aligned ATCC 43144 and ATCC 43143 proteins. Alignments with percentage identity below 30 were considered highly dissimilar and were regarded as proteins with no BLAST hits and given a value of zero.
