# Supplementary material for: Sequencing and Comparative Genome Analysis of Two Pathogenic Streptococcus gallolyticus Subspecies: Genome Plasticity, Adaptation and Virulence
Source: PLoS One. 2011 May 25;6(5):e20519. doi: 10.1371/journal.pone.0020519 (PMC3102119; doi:10.1371/journal.pone.0020519)
Supplement: Table S7 — Protein conservation of the cps loci of S. gallolyticus ATCC 43143, S. pasteurianus ATCC 43144 and S. gallolyticus UCN34. A summary table showing the BLASTP results of the genes in the cps loci. (DOC) [file pone.0020519.s010.doc]

**Table S7. Protein conservation of the *cps* loci of *S. gallolyticus* ATCC 43143, *S. pasteurianus* ATCC 43144 and *S. gallolyticus* UCN34.** A summary table showing the BLASTP results of the genes in the *cps* loci.

| **Locus ID ^a)^** | **Gene Name** | **Description** | **Most Conserved Species ^b)^** | **% Identity** | **% Query Coverage** | **% Hit Coverage** |
| --- | --- | --- | --- | --- | --- | --- |
| GALLO_0942 | *cpsX* | transcription regulator LytR | *S. gallolyticus* |  |  |  |
| GALLO_0943 | *cpsY* | transcription regulator LysR | *S. gallolyticus* |  |  |  |
| GALLO_0944 | *cpsA* | transcriptional activator in exopolysaccharide biosynthesis | *S. gallolyticus* |  |  |  |
| GALLO_0945 | *cpsB* | protein-tyrosine phosphatase | *S. gallolyticus* |  |  |  |
| GALLO_0946 | *cpsC* | chain length determinant protein | *S. gallolyticus* |  |  |  |
| GALLO_0947 | *cpsD* | protein-tyrosine phosphatase | *S. gallolyticus* |  |  |  |
| GALLO_0948 | *cpsE* | glycosyltransferase in exopolysaccharide biosynthesis | *S. gallolyticus* |  |  |  |
| GALLO_0949 | *cpsF* | rhamnosyl transferase | *S. suis* | 78.90% | 99.20% | 99.70% |
| GALLO_0950 | *cpsH* | polysaccharide polymerase | *S. pneumoniae* | 62.10% | 100.00% | 99.70% |
| GALLO_0951 | *cpsI* | glycosyl transferase | *S. pneumoniae* | 66.50% | 100.00% | 100.00% |
| GALLO_0952 | *cpsJ* | sugar transferase | *S. pneumoniae* | 59.90% | 99.30% | 100.00% |
| GALLO_0953 | *cpsK* | polysaccharide flippase transporter | *S. pneumoniae* | 57.50% | 96.60% | 98.70% |
| GALLO_0954 | *cpsL* | CDP-glycerol:polyglycerol phosphate glycero-phosphotransferase | *S. pneumoniae* | 51.20% | 98.20% | 99.20% |
| GALLO_0955 | *cpsM* | glycerol-3-phosphate cytidylyltransferase | *S. mitis* | 47.30% | 99.20% | 99.50% |
| SGGB_0926 | *cpsX* | transcription regulator LytR | *S. gallolyticus* |  |  |  |
| SGGB_0927 | *cpsY* | transcription regulator LysR | *S. gallolyticus* |  |  |  |
| SGGB_0928 | *cpsA* | transcriptional activator in exopolysaccharide biosynthesis | *S. gallolyticus* |  |  |  |
| SGGB_0929 | *cpsB* | protein-tyrosine phosphatase | *S. gallolyticus* |  |  |  |
| SGGB_0930 | *cpsC* | chain length determinant protein | *S. gallolyticus* |  |  |  |
| SGGB_0931 | *cpsD* | protein-tyrosine phosphatase | *S. gallolyticus* |  |  |  |
| SGGB_0932 | *cpsE* | glycosyltransferase in exopolysaccharide biosynthesis | *S. gallolyticus* |  |  |  |
| SGGB_0933 | *cpsF* | glycosyltransferase in exopolysaccharide biosynthesis | *S. thermophilus* | 93.30% | 100.00% | 100.00% |
| SGGB_0934 | *cpsG* | glycosyltransferase in exopolysaccharide biosynthesis | *S. thermophilus* | 72.00% | 99.40% | 96.40% |
| SGGB_0935 | *cpsH* | glycosyl transferase | *S. bovis* | 50.10% | 96.50% | 95.90% |
| SGGB_0936 | *cpsI* | glycosyl transferase family 1 | *Lactobacillus salivarius* | 35.70% | 98.90% | 98.10% |
| SGGB_0937 | *cpsJ* | glycosyl transferase family 2 | *Lactobacillus amylovorus* | 39.00% | 98.30% | 97.70% |
| SGGB_0938 | pseudogene | truncated polysaccharide polymerase | *Clostridium nexile* | 40.80% | 50.30% | 17.70% |
| SGGB_0939 | pseudogene | truncated polysaccharide polymerase | *Clostridium nexile* | 31.10% | 86.00% | 46.60% |
| SGGB_0940 | *cpsM* | glycosyltransferase in exopolysaccharide biosynthesis | *Fusobacterium varium* | 40.90% | 92.10% | 89.30% |
| SGGB_0941 | *wzx* | polysaccharide flippase transporter | *Bryantella formatexigens* | 54.40% | 99.80% | 97.50% |
| SGGB_0942 | *cpsO* | polysaccharide pyruvyl transferase | *Lactobacillus paracasei* | 31.70% | 96.30% | 97.30% |
| SGGB_0943 | *cpsP* | glycosyl transferase family 2 | *Clostridium botulinum* | 59.60% | 99.40% | 97.90% |
| SGGB_0944 | *cpsQ* | short-chain dehydrogenase/reductase SDR | *Clostridium botulinum* | 44.50% | 100.00% | 100.00% |
| SGPB_0807 | *cpsX* | transcription regulator LytR | *S. gallolyticus* |  |  |  |
| SGPB_0808 | *cpsY* | transcription regulator LysR | *S. gallolyticus* |  |  |  |
| SGPB_0809 | *cpsA* | transcriptional activator in exopolysaccharide biosynthesis | *S. gallolyticus* |  |  |  |
| SGPB_0810 | *cpsB* | protein-tyrosine phosphatase | *S. gallolyticus* |  |  |  |
| SGPB_0811 | *cpsC* | chain length determinant protein | *S. gallolyticus* |  |  |  |
| SGPB_0812 | *cpsD* | protein-tyrosine phosphatase | *S. gallolyticus* |  |  |  |
| SGPB_0813 | *cpsE* | polysaccharide biosynthesis protein | *S. uberis* | 64.30% | 98.90% | 93.70% |
| SGPB_0814 | *cpsF* | aminotransferase family protein | *Mollicutes bacterium* | 70.40% | 99.80% | 99.30% |
| SGPB_0815 | *cpsG* | bacterial sugar transferase | *Desulfotomaculum reducens* | 65.50% | 94.10% | 91.80% |
| SGPB_0816 | *cpsH* | glycosyl transferase family 2 | *Ruminococcus flavefaciens* | 68.80% | 98.40% | 100.00% |
| SGPB_0817 | *cpsI* | glycosyltransferase | *Blautia hydrogenotrophica* | 50.00% | 87.30% | 91.00% |
| SGPB_0818 | *cpsJ* | glycosyl transferase family 1 | *Clostridium thermocellum* | 40.10% | 99.40% | 99.10% |
| SGPB_0819 | *cpsK* | hypothetical protein | *-* | - | - | - |
| SGPB_0820 | *cpsL* | glycosyl transferase family 2 | *S. pneumoniae* | 46.40% | 100.00% | 99.00% |
| SGPB_0821 | *wzy* | polysaccharide polymerase | *S. pneumoniae* | 36.70% | 91.20% | 89.00% |
| SGPB_0822 | *wzx* | polysaccharide flippase transporter | *Lactobacillus crispatus* | 40.10% | 85.50% | 96.90% |
| SGPB_0823 | *cpsO* | UDPglucose 6-dehydrogenase | *Coprococcus sp.* | 75.10% | 100.00% | 100.00% |

1. ID starts with “GALLO_” are genes from *S. gallolyticus* UCN34, “SGGB_” are from *S. gallolyticus* ATCC 43143 and “SGPB_” are from *S. pasteurianus* ATCC 43144.
2. The most conserved bacterial species during BLASTP alignment with the NCBI ‘nr’ database.
