# Supplementary material for: Sequencing and Comparative Genome Analysis of Two Pathogenic Streptococcus gallolyticus Subspecies: Genome Plasticity, Adaptation and Virulence
Source: PLoS One. 2011 May 25;6(5):e20519. doi: 10.1371/journal.pone.0020519 (PMC3102119; doi:10.1371/journal.pone.0020519)
Supplement: Table S8 — List of proteins contain the LPXTG anchoring motifs in S. gallolyticus ATCC 43143 and S. pasteurianus ATCC 43144. (DOC) [file pone.0020519.s011.doc]

**Table S8. List of proteins contain the LPXTG anchoring motifs in *S. gallolyticus* ATCC 43143 and *S. pasteurianus* ATCC 43144.**

| **ATCC 43143 ^a), b)^** | **Descriptions** | **ATCC 43144 ^a)^** |
| --- | --- | --- |
| SGGB_0110 * | fructan beta-fructosidase (exo-inulinase) | NA |
| SGGB_0154 | cell wall surface protein | NA |
| SGGB_0180 | cell wall surface protein | NA |
| SGGB_0376 | PTS system, mannose-specific IID component | SGPB_0301 |
| SGGB_0443 | phospho-N-acetylmuramoyl-pentapeptide-transferase | SGPB_0369 |
| SGGB_0453 ^#^ | penicillin binding protein 1A | SGPB_0380 |
| SGGB_0544 * | Cna protein B-type domain-containing protein | NA |
| SGGB_0670 | cell wall surface protein | NA |
| SGGB_0730 | lactocepin | SGPB_0626 * (pseudogene) |
| NA | cell wall surface protein | SGPB_0680 * |
| SGGB_0837 | putative ABC transport system permease protein | SGPB_0722 |
| NA | major facilitator superfamily permease | SGPB_0884 |
| SGGB_1047 * | glucan-binding protein C, GbpC | NA |
| SGGB_1138 ^#^ | phosphate transport system permease protein | SGPB_1006 |
| NA | glucan-binding protein C family protein | SGPB_1131 * |
| SGGB_1458 * | type II secretory pathway, pullulanase PulA and related glycosidases | SGPB_1362 * |
| SGGB_1567 * | fimbrial subunit B protein FszB | NA |
| SGGB_1568 * | Cna protein B-type domain-containing protein | NA |
| SGGB_1602 * | cell wall surface protein | NA |
| SGGB_1650 | major facilitator superfamily protein | SGPB_1507 |
| NA | Cna protein B-type domain-containing protein | SGPB_1661 * |
| SGGB_1664 | cell wall surface protein | NA |
| SGGB_1687 | cell wall surface protein | NA |
| SGGB_1964 | PTS system, galactitol-specific IIC component | NA |
| SGGB_2003 | putative bacteriocin | NA |
| SGGB_2016 * | Cna protein B-type domain-containing protein | NA |
| SGGB_2021 * | Cna protein B-type domain-containing protein | SGPB_1846 * |
| SGGB_2022 * | cell wall ribonucleases G and E | SGPB_1847 * |
| SGGB_2170 * | cell wall surface protein | NA |
| SGGB_2171 * | cell wall surface protein | NA |
| SGGB_2210 * | Cna protein B-type domain-containing protein | NA |
| SGGB_2211 * | collagen binding domain-containing protein | NA |
| SGGB_2263 | cell wall surface protein | SGPB_1989 |

1. ATCC43143 and ATCC 43144 ORFs that were also detected to contain TIGRFAM LPXTG-motif (HMM model ID: TIGR01167) were marked with asterisk (*).
2. ATCC43143 ORFs that was not detected to contain any LPXTG motif but is homologous to ATCC 43144 were marked with hash (#).
