# Supplementary material for: Sequencing and Comparative Genome Analysis of Two Pathogenic Streptococcus gallolyticus Subspecies: Genome Plasticity, Adaptation and Virulence
Source: PLoS One. 2011 May 25;6(5):e20519. doi: 10.1371/journal.pone.0020519 (PMC3102119; doi:10.1371/journal.pone.0020519)
Supplement: Table S9 — List of predicted lipoproteins in S. gallolyticus ATCC 43143 and S. pasteurianus ATCC 43144. (DOC) [file pone.0020519.s012.doc]

**Table S9. List of predicted lipoproteins in *S. gallolyticus* ATCC 43143 and *S. pasteurianus*** ATCC 43144.

| **ATCC 43143** | **Descriptions** | **ATCC 43144** |
| --- | --- | --- |
| SGGB_0114 | multiple sugar transport system substrate-binding protein | NA |
| SGGB_0120 | ABC transporter substrate binding lipoprotein | SGPB_0116 |
| SGGB_0139 | predicted lipoprotein | NA |
| SGGB_0179 | predicted lipoprotein | NA |
| SGGB_0207 | extracellular solute-binding protein | SGPB_0150 |
| SGGB_0302 | predicted lipoprotein | NA |
| NA | multiple sugar transport system substrate-binding protein | SGPB_0179 |
| SGGB_0352 | peptide/nickel transport system substrate-binding protein | SGPB_0276 |
| SGGB_0386 | predicted lipoprotein | SGPB_0316 |
| SGGB_0445 | polar amino acid transport system substrate-binding protein | SGPB_0371 |
| SGGB_0508 | predicted lipoprotein | NA |
| SGGB_0599 | predicted lipoprotein | NA |
| SGGB_0601 | L,D-transpeptidase lipoprotein | NA |
| SGGB_0677 | predicted lipoprotein | NA |
| SGGB_0721 | N-acetylmuramoyl-L-alanine amidase | SGPB_0611 |
| SGGB_0734 | predicted lipoprotein | NA |
| SGGB_0859 | iron complex transport system substrate-binding protein | NA |
| NA | predicted lipoprotein | SGPB_0751 |
| SGGB_0868 | predicted lipoprotein | SGPB_0756 |
| SGGB_0917 | extracellular tannase, alpha/beta superfamily hydrolase | NA |
| SGGB_0989 | bacterial extracellular solute-binding lipoprotein, family 3 | SGPB_0861 |
| SGGB_1030 | predicted lipoprotein | SGPB_0902 |
| SGGB_1107 | predicted lipoprotein | NA |
| SGGB_1126 | simple sugar transport system substrate-binding protein | SGPB_0995 |
| SGGB_1141 | phosphate transport system substrate-binding protein | NA |
| SGGB_1228 | putative glutamine transport system substrate-binding protein | NA |
| SGGB_1240 | predicted lipoprotein | NA |
| NA | phosphate transport system substrate-binding protein | SGPB_1008 |
| NA | predicted lipoprotein | SGPB_1073 |
| NA | carboxylesterase type B | SGPB_1074 |
| NA | nisin immunity protein | SGPB_1100 |
| NA | predicted lipoprotein | SGPB_1156 |
| NA | GNAT family acetyltransferase | SGPB_1197 |
| SGGB_1361 | predicted lipoprotein | SGPB_1288 |
| SGGB_1367 | predicted lipoprotein | SGPB_1293 |
| SGGB_1386 | polar amino acid transport system substrate-binding protein | SGPB_1307 |
| SGGB_1388 | polar amino acid transport system substrate-binding protein | NA |
| SGGB_1389 | polar amino acid transport system substrate-binding protein | SGPB_1310 |
| SGGB_1393 | maltose/maltodextrin transport system substrate-binding protein | SGPB_1314 |
| SGGB_1406 | peptide/nickel transport system substrate-binding protein | SGPB_1328 |
| SGGB_1407 | peptide/nickel transport system substrate-binding protein | NA |
| SGGB_1585 | predicted lipoprotein | NA |
| SGGB_1616 | predicted lipoprotein | NA |
| SGGB_1665 | predicted lipoprotein | NA |
| SGGB_1698 | predicted lipoprotein | NA |
| SGGB_1704 | peptidyl-prolyl cis-trans isomerase A (cyclophilin A) | SGPB_1517 |
| NA | endo-beta-N-acetylglucosaminidase | SGPB_1523 |
| NA | multiple sugar transport system substrate-binding protein | SGPB_1525 |
| SGGB_1747 | branched-chain amino acid transport system substrate-binding protein | SGPB_1570 |
| SGGB_1759 | iron complex transport system substrate-binding protein | SGPB_1580 |
| SGGB_1765 | predicted lipoprotein | NA |
| SGGB_1801 | preprotein translocase YidC subunit | SGPB_1631 |
| SGGB_1831 | D-methionine transport system substrate-binding protein | NA |
| SGGB_1832 | polar amino acid transport system substrate-binding protein | SGPB_1681 |
| SGGB_1891 | predicted lipoprotein | SGPB_1736 |
| SGGB_1915 | predicted lipoprotein | SGPB_1756 |
| SGGB_1949 | phosphate transport system substrate-binding protein | SGPB_1798 |
| SGGB_2030 | iron/zinc/copper transport system substrate-binding protein | SGPB_1854 |
| SGGB_2060 | preprotein translocase YidC subunit | SGPB_1869 |
| SGGB_2113 | D-methionine transport system substrate-binding protein | NA |
| SGGB_2166 | predicted lipoprotein | NA |
| SGGB_2248 | predicted lipoprotein | NA |
